# Fast and accurate mutation detection in whole genome sequences of multiple isogenic samples with IsoMut

**DOI:** 10.1186/s12859-017-1492-4

**Published:** 2017-01-31

**Authors:** O. Pipek, D. Ribli, J. Molnár, Á. Póti, M. Krzystanek, A. Bodor, G. E. Tusnády, Z. Szallasi, I. Csabai, D. Szüts

**Affiliations:** 1 Department of Physics of Complex Systems, Eötvös Loránd University, H-1117 Budapest, Hungary; 2 Institute of Enzymology, Research Centre for Natural Sciences, Hungarian Academy of Sciences, H-1117 Budapest, Hungary; 3 Center for Biological Sequence Analysis, Department of Systems Biology, Technical University of Denmark, DK-2800 Lyngby, Denmark; 4 Computational Health Informatics Program (CHIP), Boston Children’s Hospital, Boston, USA; 5 Harvard Medical School, Boston, MA 02215 USA; 6 MTA-SE-NAP, Brain Metastasis Research Group, 2nd Department of Pathology, Semmelweis University, H-1091 Budapest, Hungary

**Keywords:** Next generation sequencing, Mutagenesis, Somatic mutation detection, Multiple isogenic samples, Low false positive rate, Demonstrative algorithm

## Abstract

**Background:**

Detection of somatic mutations is one of the main goals of next generation DNA sequencing. A wide range of experimental systems are available for the study of spontaneous or environmentally induced mutagenic processes. However, most of the routinely used mutation calling algorithms are not optimised for the simultaneous analysis of multiple samples, or for non-human experimental model systems with no reliable databases of common genetic variations. Most standard tools either require numerous in-house post filtering steps with scarce documentation or take an unpractically long time to run. To overcome these problems, we designed the streamlined IsoMut tool which can be readily adapted to experimental scenarios where the goal is the identification of experimentally induced mutations in multiple isogenic samples.

**Methods:**

Using 30 isogenic samples, reliable cohorts of validated mutations were created for testing purposes. Optimal values of the filtering parameters of IsoMut were determined in a thorough and strict optimization procedure based on these test sets.

**Results:**

We show that IsoMut, when tuned correctly, decreases the false positive rate compared to conventional tools in a 30 sample experimental setup; and detects not only single nucleotide variations, but short insertions and deletions as well. IsoMut can also be run more than a hundred times faster than the most precise state of art tool, due its straightforward and easily understandable filtering algorithm.

**Conclusions:**

IsoMut has already been successfully applied in multiple recent studies to find unique, treatment induced mutations in sets of isogenic samples with very low false positive rates. These types of studies provide an important contribution to determining the mutagenic effect of environmental agents or genetic defects, and IsoMut turned out to be an invaluable tool in the analysis of such data.

**Electronic supplementary material:**

The online version of this article (doi:10.1186/s12859-017-1492-4) contains supplementary material, which is available to authorized users.

## Background

Next generation sequencing offers a powerful tool to investigate genetic aberrations in a comprehensive manner on a wide scale, ranging from point mutations [1] to large-scale genomic rearrangements [2]. However, low complexity genomic regions, artefacts produced by the NGS pipeline, and regions of homology across diverse parts of the genome often make it difficult to produce a reliable call on a given somatic single nucleotide variation (SNV) [3–6]. SNV identification is further hampered when no information is available on common variations among individuals (single nucleotide polymorphisms – SNPs). A well-annotated reference genome, such as the human genome, and the use of appropriate controls, such as sequencing of matching germline DNA, can significantly reduce the effects of these problems. However, in many experimental setups such control reference genomes are not available. Also, even though NGS is a very effective way of genome analysis, it generates sequencing errors that may be falsely detected as mutations [7, 8].

While the human genome is relatively well-researched and extensive effort was put into retrieving information on variation among humans [9, 10] to reduce the detection of false positive mutations, the case of less commonly sequenced organisms and cell lines is different. Also, the practice of repeat masking of the genome is usually either unavailable or less reliable for non-human organisms. As most publicly available mutation detection software tools are optimised for human genomes and also for specific experimental scenarios such as cancer genome analysis, it may be expected that they do not perform satisfactorily in many other experimental designs.

One of the most common ways of overcoming these difficulties and adjusting already existing software to the special needs of a given experiment is running the tool with default settings and applying in-house scripts with little or no documentation to remove false positive calls. These methods are rarely tested or optimised and do not allow the straightforward reproduction of the results, which presents a great disadvantage when one attempts to compare scientific findings.

A specific, but relatively straightforward mutagenesis experimental set-up involves a population of essentially identical starting cells which over the course of the experiment individual cells are expected to acquire different treatment-induced sets of mutations. Such experiments are routinely used to survey the mutagenic effect of various drugs or environmental agents [11, 12], to detect mutations that contribute to the development of treatment resistance [13, 14], or to identify mutagenic processes dependent on various genetic backgrounds [15]. In the past, the read-out of mutagenesis assays was commonly obtained from the sequence of a single gene, but whole genome sequencing can provide a broader, unbiased mutation dataset. During our work on chicken DT40 cells, we found that whole genome sequencing data from mutagenesis experiments could not be processed with sufficient reliability by routinely used mutation detection software, such as VarScan 2 [16] or MuTect [17], even when tuning the appropriate control parameters of the tools. MuTect2, which was not yet available at the time of this study, is a more sophisticated version of MuTect and is able to detect indels (insertions and deletions) besides SNVs, however it would have taken unfeasibly long to run on our experimental data.

In this manuscript we describe a very fast method for accurate somatic mutation calling that is adequate when multiple, differently treated isogenic samples are investigated, by using information from many available samples to rule out false positives. Samples were derived from single cell clones, and we made use of the assumption that mutations are independent in each sample. Therefore, our method identifies SNVs and short indels present in a single sample only, filtering out SNPs, sequencing and alignment bias primarily on the basis that the false positive calls tend to be present at the same genomic location in multiple samples. This way, the need for a well-annotated reference genome or pre-existing databases of germline variants is eliminated. IsoMut applies a very simple strategy for filtering by using fixed thresholds for most of the filtering parameters which are in clear connection with the actual sequencing data, allowing the user to easily interpret the results without dwelling deep into statistical models. On the other hand, IsoMut also provides an additional filtering option which is based on the statistical Fisher’s exact test and can be used to finely tune the results to remove all false positive calls from control samples if such samples are available. We successfully used IsoMut to measure the mutagenic effect of common cancer chemotherapeutics [18] and to determine the effect of DNA repair gene defects on mutagenesis [19]. In this paper we present proof for the accuracy of mutation detection using IsoMut.

## Methods

### Dataset

Our method was optimised using a dataset of whole genome sequences, obtained from a panel of cell line clones used for assessing the effect of various chemical agents on mutagenesis. The DT40 chicken lymphoblastoma cell line [20] was used for the experiments; the wild type and *BRCA1* ^*-/-*^ cell clones used in this work were derived from a previous study [19]. Single cell clones were isolated and expanded before sample preparation. Instead of sequencing a mixture of genomes in a population, this arrangement allowed us to derive the sequences of the individual cloned cells, as any mutation arising during the clonal expansion would only be present in a small proportion of the sequence reads and thus filtered out during the analysis. The experimental setup results in an expected number of 50–5000 mutations in each sample, similar to or lower than the number of mutations found in cancer samples [21]. This relatively low overall mutation rate made it crucial to keep false positive mutations to an absolute minimum.

Altogether *N* = 30 samples were analysed, each of them identified by a unique ID (see Additional file 1 for detailed table). Samples differ from each other in both their genotype (‘WT’ (wild type) or ‘Mutant1’) and their treatment. Mutant1 samples carry a homozygous BRCA1 mutation that deletes exons 6–8 of the gene [19]. However, as the actual genotypes and treatments are irrelevant to the purposes of this paper, only general names are used below. The genome sequences of samples with same genotype and treatment are not identical, as they arose from distinct cell clones. The only identical samples in the dataset are two pairs (S12, S15 and S27, S30), which were acquired by sequencing the same DNA preparation twice. The availability of repeat samples allowed us to control for false positive mutations occurring due to sequencing and alignment error.

Whole genome sequence data was obtained by Illumina paired end sequencing with read sizes of 125 and 150 bases in two sequencing batches. The different read lengths and variations in other sequencing parameters among sample groups are not unusual in real studies and present the substantial challenge of reliably comparing genetic information that may have been influenced by various kinds of instrumental and computational artefacts.

Sequencing data generated for this study have been submitted to the European Nucleotide Archive (ENA; http://www.ebi.ac.uk/ena/) under study accession number ERP014915.

### Preparation of input files

As IsoMut uses BAM files as its input, and optimisation steps described below were carried out on pileup files generated from these, reads were first aligned to the chicken (Gallus gallus) reference sequence Galgal4.73, which was downloaded from Ensembl [22]. The alignment was made using the Burrows-Wheeler Alignment Tool (BWA, version 0.7.5a-r405). The reference sequence was indexed with the BWT-SW algorithm [23], which is recommended in the case of large genomes. The alignments of paired-end reads were generated with the bwa-mem algorithm [24]. Duplicated reads were removed using the samblaster program [25]. Additionally the aligned reads were realigned near indels by the GATK IndelRealigner [26]. After the generation of BAM files, a joint and filtered pileup file of all investigated samples was created (using the samtools [27] mpileup command with options ‘-B’ and ‘-Q 30’) for time management purposes, as we needed to have access to this information repeatedly. Further details on the generation of pileup files can be found in Additional file 2 and Additional file 3.

### Mutation detection method based on multi-sample noise filtering

**Fig. 1:**
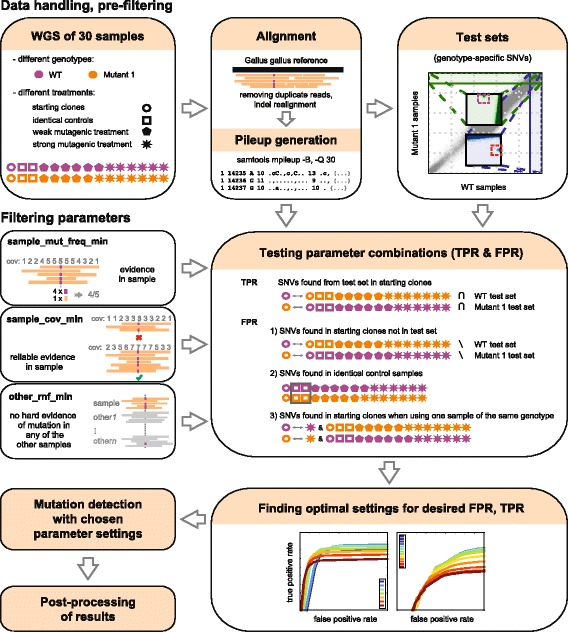
An overview of the testing and optimisation of the mutation detection method

As the naïve approach of using commonly applied mutation detection tools with the suggested default settings failed to produce satisfactory results or could not detect small indels (for details see the Discussion section), we designed a filtering method that combines information from all available samples and gives robust SNV and indel calling with low false positive rate. A general overview of the method can be seen on Fig. 1. The method looks for heterozygous mutations with respect to the reference genome, and filters out positions where other samples also differ from the reference. This approach ensures that ‘germline’ variations, present in multiple samples, are not called as false ‘somatic’ mutations even in the absence of an available SNP database. The other common source of false positive mutation calls are alignment errors. Typically they occur at the same genome positions in multiple samples, so with multi-sample filtering they are easily eliminated.

Results were evaluated using a set of validated genotype-specific SNVs (‘test sets’), the generation of which is described below. The calculated true positive and false positive rates (TPR/FPR) were used as indicators of the goodness of the filtering and optimisation was carried out based on these values.

### Establishing SNV test sets

To measure false negatives and validate the SNV detection results, we established two different high-confidence reference SNV sets from within our dataset. The test sets consist altogether of around 4000 positions, which is a sufficiently large number to calculate reliable estimates of TPR and FPR values.

The cell clone whole genome sequence panel used contains several isogenic samples of two different genotypes (WT and Mutant 1) that underwent various mutagenic treatments. Cell populations were grown separately for some time, accumulating mutations, before the isolation of single clones for genome sequencing. Therefore, we expected to find two types of SNVs within our dataset. There should be treatment-induced, primarily heterozygous SNVs present in individual samples only. In addition, there would be SNVs arising from the genetic differences of the starting WT and Mutant 1 cell clones, which could be either heterozygous or homozygous. Heterozygous SNVs of the latter category were used as test set positions.

**Fig. 2:**
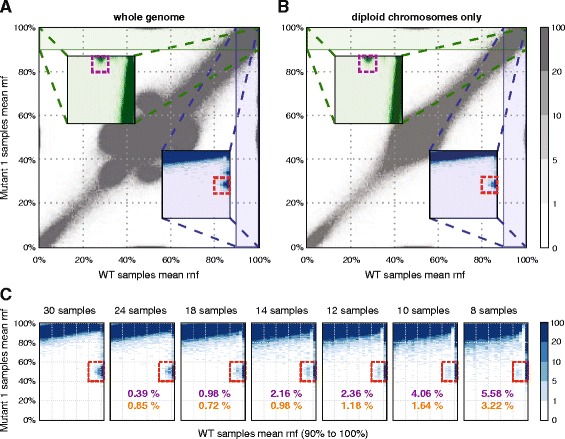
Test set detection for WT and Mutant 1 samples. **a**, **b** Plots of mean reference nucleotide frequency values in the samples of the two geno-types; **a** whole genome, **b** diploid chromosomes only. Insets are zoomed-in regions of the underlying plot. Dashed rectangles mark the clusters identified as test cohorts. **c** Generating the same figures for different sample numbers. Percentages in purple show the ratio of lost test set positions, while percentages in orange represent the ratio of gained positions in the area in the dashed rectangle

A plot of the mean reference nucleotide frequency (*rnf*) of all WT samples against the mean *rnf* of all Mutant 1 samples readily identifies heterozygous SNVs present in the Mutant 1 genotype at the [100, 50%] position. Mutations present in the WT samples but not in the Mutant 1 samples are clustered around [50, 100%] (see Fig. 2a). Additional clusters around [100, 70] and [70, 100%] are due to genomic regions with ploidy > 2 in the experimental cell line. To verify this statement, we replotted the previous figure using only disomic chromosomes (Fig. 2b) and used the clusters on this latter graph as test cohorts, as the resulting clusters have very clear outlines and show no overlap with the rest of the data. Thus all optimisation procedures were limited to disomic chromosomes only, which proved to be sufficient for the relatively stable DT40 genome [20]. In cases when ploidy varies greatly in the investigated genome, a ploidy-specific optimisation should be carried out. Also, it is impossible to separate loss of heterozygosity (LOH) events from real germline mutations during test set generation. This is not a problem, as LOH regions can be included in the reference sets for testing purposes, increasing the number of positions which can be used for statistical analysis.

The described method for generating test sets is applicable whenever two sample groups of related but separate genetic origin are available. Details of the very similar indel test set generation can be found in Additional file 2. In-depth workflow of the test set generation from pileup files for both SNVs and indels is in Additional file 4.

### Testing of different filtering methods

Regardless of the SNV detection method selected, the above test sets can be used to determine their accuracy. False positive rates (FPR) were calculated by running IsoMut on all samples, and counting independent mutations in pre-treatment starting cell clones and in the repeatedly sequenced identical control samples, in which no independent mutations may be present. To obtain estimates for the true positive rate (TPR), we ran the algorithm using the starting clone of one genotype and all clones of the second genotype. This way, the genotype-specific SNVs were detected as unique in the selected starting clone, and the proportion of the respective genotype-specific test set detected in this individual sample was used as TPR. Further details can be found in Additional file 2.

### Defining filtering parameters to handle SNPs and alignment noise

IsoMut detects somatic mutations by considering all input samples and applying filtering criteria at each genomic position. To effectively discard germline mutations and false positives arising from alignment noise, three basic filtering parameters were introduced: a minimal threshold for the ratio of the most common type of non-reference reads in the investigated sample (*sample_mut_freq_min*), a minimal threshold for the ratio of reference reads in the noisiest non-selected (‘other’) sample (*other_rnf_min*) and a coverage limit for the selected sample (*sample_cov_min*). For detailed verification of using such filters, see Additional file 5. These parameters were optimised for the desired values of TPR and FPR using the above described testing method.

This thorough optimisation procedure requires a very specific experimental setup and is often not feasible with the available set of sequencing data. Thus a more rapid and convenient method is desired to adopt IsoMut to specific needs. To provide a much simpler tuning option, IsoMut calculates the *S* score value of each candidate mutation, which is inversely related to the probability of falsely categorising a position as a unique mutation, thus high-confidence mutations have higher *S* values than unlikely ones. More precisely, *S* is calculated as the negative logarithm of the probability *p*, that given the assumption that the distribution of bases in the two noisiest samples (containing the most non-reference reads) at the genomic position is the same, we would observe the actual sequencing data. Thus a low *p* (high *S*) value suggests that it is unlikely that the two investigated samples have the same base-distribution, making it likely that the noisiest sample indeed has a unique mutation in the given position. The probability *p* is determined by the Fisher’s exact statistical test. For more details, see Additional file 2.

The availability of negative controls allows for a simple, yet effective tuning of IsoMut by setting an *S* score threshold for more rapid results. In this case, a separate optimisation can be carried out on SNVs, insertions and deletions.

### Post-processing

SNV and indel candidates were subjected to different post-processing steps, the details of which can be found in Additional file 2.

## Results and discussion

### Optimal threshold values

**Fig. 3:**
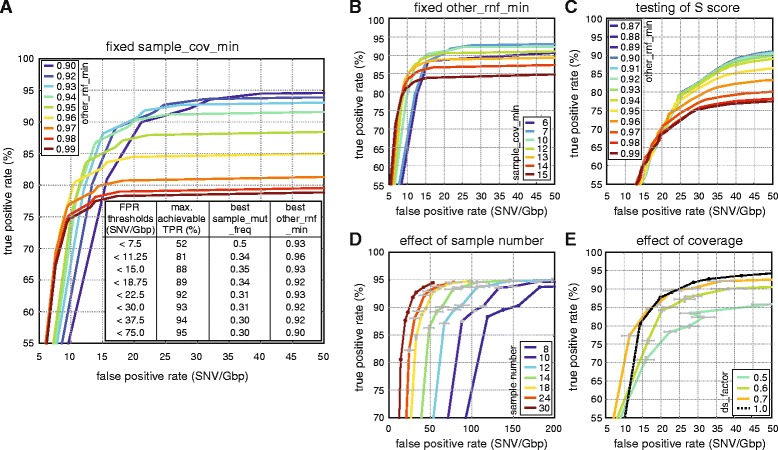
Quality components resulting from different parameter settings and different datasets. **a** Effects of varying *other_rnf_min* (*different curves*) and *sample_mut_freq_min* (*along the curves*) with constant *sample_cov_min* = 7. The inset contains maximal achievable TPRs for given FPR thresholds with the optimal parameter settings. **b** Effects of changing *sample_cov_min* (different curves) and *sample_mut_freq_min* (*along the curves*) with fixed *other_rnf_min* = 0.93. **c** Effects of varying *other_rnf_min* (*different curves*) and the *S* score parameter (along the curves) with *sample_cov_min* = 5 and *sample_mut_freq_min* = 0.21. **d** Effects of varying the size of the dataset. Measurement points correspond to the parameter settings of the inset of (**a**). Mean values and standard deviation of three randomly chosen datasets are shown (see Additional file 2). **e** Effects of decreased sample coverage. Measurement points correspond to the parameter settings of the inset of (**a**). Mean values and standard deviation of three randomly down-sampled measurements are shown (see Additional file 2)

We tested IsoMut on whole genome sequences of mutagen-treated cultured cells. In the tests described below, worst case scenarios were considered. One test for determining the FPR was the inclusion of only two samples of a given genotype, which makes the testing procedure as strict as possible. (For more details, see Additional file 2.) If samples are distributed more evenly among genotypes, IsoMut can achieve even better results than the ones presented below, simply by adjusting the *S* score parameter. (For an example, see Additional file 6.) Filter threshold values were selected based on TPR and FPR requirements. We tested the effect on TPR and FPR of varying the *sample_mut_freq_min* filter at different values of *sample_cov_min* coverage requirement and a fixed *other_rnf_min* = 0.93 (Fig. 3b). As the depth of the sequencing data limits coverage thresholds, we chose an intermediate, fixed *sample_cov_min* = 7 value for further optimising. At this value, we investigated how varying *sample_mut_freq_min* at different values of the *other_rnf_min* filtering parameter influences the relation of TPR and FPR (Fig. 3a). Depending on the number of expected mutations in the investigated samples, different FPR values can be tolerated. The inset on Fig. 3a shows the corresponding maximal achievable TPRs and optimal filtering parameters to arbitrarily chosen FPR values. For the low FPR requirement of the test sample set, a fixed parameter set of *sample_mut_freq_min* = 0.31, *other_rnf_min* = 0.93, *sample_cov_min* = 7 provided the best TPR of 92%. Further parameter settings with the respective TPR and FPR values can be found in Additional file 7.

### Effects of sample number and sequencing depth

To assess the effects of having smaller datasets with fewer samples, different *n* sized subsets of the original 30 samples were investigated. Details on this technique can be found in Additional file 2. When using fewer samples, the outline of mutation clusters used for establishing a test set gets progressively more blurred (Fig. 2c), but the number of lost and gained positions remains relatively small (less than 6 and 4% of the original set, respectively) even for only 10 samples. The dominant effect of reducing the number of available samples is an increased FPR (Fig. 3d). For very strict parameter settings the number of true positives also increases when using smaller datasets. In case of the samples used for demonstration here, to keep FPRs below 50 per genome (~1 GB) while maintaining a TPR of at least 85%, no fewer than 14 samples are required for analysis.

To demonstrate how a decreased coverage in one of the samples effects the results, we down-sampled the sequence read data of the Mutant 1 starting clone using different down-sampling factors (*ds_factors*) of 0.7, 0.6 and 0.5 and recalculated TPRs and FPRs for the parameter settings shown in the inset of Fig. 3a. Further details are included in Additional file 2. We found that having 70% of the original coverage had minimal impact on mutation detection, but further decreasing the sequencing depth produced lower TPR and higher FPR values. As the Mutant 1 starting clone had a mean coverage of 21, we advise using samples with a mean coverage of at least 15.

### IsoMut software implementation – guidelines for different experimental setups

We created an open-source C implementation of the somatic mutation detection steps of the above algorithm with a python wrapper for parallelisation (downloadable from https://github.com/genomicshu/isomut). The tool expects BAM files as its input and returns a list of detected mutations (both SNVs and indels) by applying predefined filtering parameters and a post-processing step different for SNVs and indels (see Additional file 2) in each genomic position. Thus an appropriate reference genome is necessary for running IsoMut for alignment purposes, but mutations are not detected based on differences of the samples and the reference genome, but on differences between investigated samples.

IsoMut can be applied whenever multiple isogenic samples are available and unique mutations are sought. Negative control samples should be used when possible. These can be either pre-experiment starting clones or DNA preparations sequenced multiple times, neither of which should contain experiment-induced unique mutations. With the availability of negative controls and a positive control test mutation set, best results are achieved by optimising the three IsoMut filtering parameters as demonstrated above.

However, the availability of negative controls also allows for the tuning of the *S* score value for more rapid results, skipping the generation of positive test sets.

An example run of the IsoMut tool is shown in Additional file 6. In the following we present the main steps of the analysis. The generation of BAM alignment files is not included and should be carried out separately, prior to running IsoMut.

1. Downloading and compiling IsoMut.
2. Modifying user-specific data in the example script (file names, paths, filtering parameter values).
3. Running IsoMut.
4. Tuning of the *S* score threshold value to minimise false positives in negative control samples.

The first three steps are necessary, the fourth one is optional and requires the availability of negative control sample(s). Whenever possible, this last fine-tuning step is strongly encouraged and yields better results than using the predefined filtering parameters only. For this procedure we suggest choosing less strict values for the *sample_mut_freq_min* and *sample_cov_min* filters, and further filtering the results based on the *S* score (see Additional file 6). The effects of tuning the *S* score value and the *other_rnf_min* parameter with fixed *sample_mut_freq_min* = 0.21 and *sample_cov_min* = 5 is shown in Fig. 3c. According to the figure, whenever a very low (< 2 · 10^−8^) FPR is desired, we suggest choosing a strict *other_rnf_min* value of 0.96 (or even larger for lower FPR). When the FPR can exceed 30 per Gbp, less strict filtering is advised, *other_rnf_min* can be decreased to around 0.9. IsoMut default values are *sample_mut_freq_min* = 0.21, *sample_cov_min* = 5 and *other_rnf_min* = 0.93.

**Fig. 4:**
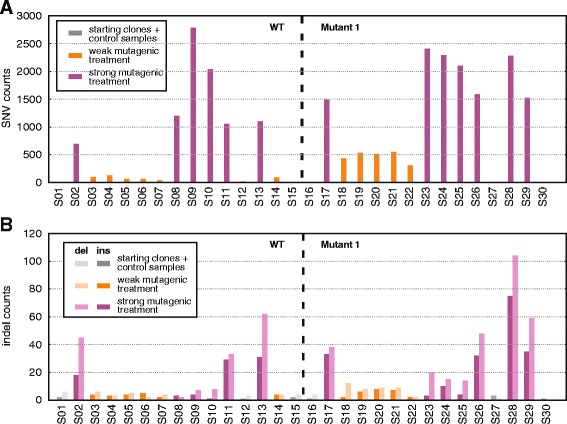
Results of running IsoMut without tuning the *S* score value. **a** SNV counts for each sample, grouped by genotype. Colours indicate the treatment of the given sample. **b** Indel counts for each sample, grouped by genotype. Colours indicate the treatment of a given sample, darker bars representing insertions, lighter ones deletions

In the absence of negative controls, step (4) should be skipped and we advise using filtering values from the inset of Fig. 3a fitting the desired FPR. In this case SNVs and indels are detected with the same filtering thresholds. More details on the performance of our method in such cases can be found in Additional file 2. An example run without using an *S* score threshold, with parameter settings *sample_mut_freq_min* = 0.31, *other_rnf_min* = 0.93, *sample_cov_min* = 7 can be found on Fig. 4 for our dataset. This resulted in an average of only 6 mutations in starting clones or identical sample pairs (FPR ~ 6∙10^−9^), even though the DT40 genome differs from the chicken reference genome in 6.3 million SNPs [20]. On the other hand, differently treated samples have up to 2790 mutations, proving that the lack of these in untreated starting clones is not due to overly strict filtering.

### Advantages of a straightforward filtering algorithm

Although setting fixed thresholds for the above described simple filtering parameters might appear unsophisticated, the approach has a general advantage over complex statistical models besides being just as effective. In spite of recent developments of bioinformatics software and mutation calling algorithms, the unspoken consensus remains that ambiguous mutation calls are best verified by checking the raw sequencing data either in a genome viewer (for example IGV [28]) or a pileup file. The above filtering parameters are directly related to the number of different bases detected at each genomic position, making the evaluation of mutations very straightforward, without the need for decoding the meaning of different *p*-values.

### Performance comparison with standard tools

We developed IsoMut because we had found that standard tools could not detect both SNVs and indels in the above described samples with the precision required for biological interpretation without heavy additional in-house filtering. Here we present a comparison with two very popular software tools, VarScan 2 [16] and MuTect [17].

VarScan 2 was run in its tumor-normal comparison mode for the two pairs of identical samples in our dataset (see Additional file 8). (Twice for both pairs, switching the roles of ‘tumor’ and ‘normal’ samples each time.) This way all mutations found by VarScan 2 are false positives. Filtering parameters and additional filtering steps were applied according to the instructions provided in [29]. The analysis resulted in 368, 410, 1264 and 922 mutations in samples S12, S15, S27 and S30 respectively. On the other hand, the numbers of false positives using IsoMut were 3, 1, 3 and 5 for the same samples. This difference in performance is probably due to the fact that VarScan 2 relies largely on filtering methods which have proved to be successful in case of human genomes, but are not available for our current dataset (dbSNP, repeat masking).

MuTect is not capable of detecting indels (however, its recently released version, MuTect2 is, but was unavailable for download at the time of this study), but we selected it for testing because besides normal-tumor sample pairs, MuTect can also use a panel of normal samples. With default settings, MuTect did not perform efficiently for our dataset, but with our control samples we were able to optimise MuTect’s LOD parameter threshold (Additional file 9), and obtained good results. Compared to MuTect IsoMut has similar characteristics at very low false positive rates (0.7/Mbp mutations detected at 0.5/Gbp FPR in our dataset), and it has higher sensitivity when we allow for higher false positive rates (1/Mbp mutations detected by IsoMut and 0.75/Mbp mutations detected by MuTect at 1/Gbp FPR, Additional file 10). Additionally we found that IsoMut adapts significantly better to lower sample numbers (Additional file 10).

To get an estimate of the runtimes of different software, we ran IsoMut, MuTect, MuTect2 and VarScan 2 on the short chicken chromosome 28 (4.7 Mbp) using the 30 samples described above. We used a modest computer with a memory of 23 GB and 12 cores, with a performance achievable in a high-end desktop computer. VarScan 2 was run in somatic mode by comparing each sample with its appropriate ‘normal’ pair, resulting in 30 comparisons. Both for MuTect and MuTect2, the general guidelines provided online were followed. First a unique panel of normal samples was created for each sample by combining the results of the artefact detection runs of all other samples. After this preliminary step, mutations were detected by comparing each sample with its ‘normal’ pair using the previously generated panel of normals. For further details on the used pipelines and scripts see Additional file 11.

Using all resources of the above described computer, IsoMut turned out to be around 170 times faster than MuTect2, more than 40 times faster than MuTect and more than 10 times faster than VarScan 2 (see Table 1). Extrapolating to the whole chicken genome, mutation analysis on the set of 30 samples using this 12-core computer would take 5 h with IsoMut, but over 35 days with Mutect2. The number of possible MuTect2, MuTect and VarScan 2 processes that can be run in parallel is limited by the finite memory of the computer, as all these software use java tools which require several java virtual machines when run in parallel. On the other hand, the parallelisation of IsoMut is only limited by the number cores on the computer and the runtime appears to be mainly I/O bound. The performance of the three java applications can be significantly improved by using a high-performance computer with a memory of 100–200 GB. However, high-end computer clusters have limited availability, and IsoMut presents a great speed advantage when using modest resources. Even though it is not realistic to run any of the above tools on a single core without parallelisation, for a more straightforward comparison the results of such a scenario are also presented in Table 1, demonstrating similar performance advantages for IsoMut.

**Table 1:** Comparison of runtimes of different tools with all available resources

Tool	12 cores				Single core	
	Number of parallel processes	Runtime	Equivalent runtime on 1 Gb genome	Runtime relative to IsoMut	Runtime	Runtime relative to IsoMut
IsoMut	12	1 min 24 s	4 h 56 min	1	7 min	1
VarScan 2	5–6	16 min	2 days 8 h	11	1 h 20 min	11
MuTect	6–7	1 h 7 min	9 days 20 h	48	4 h 55 min	42
MuTect2	4–5	4 h	35 days 5 h	171	21 h 6 min	178

Table of the runtime comparison of different mutation detection software using a computer with 23 GB memory and 12 cores or a single core only. The tools were run on the 4.735 Mb chicken chromosome 28 using the 30-sample dataset used throughout this study

## Conclusion

The described SNV identification method requires no prior knowledge of genomic nucleotide polymorphisms (SNPs). As these are expected to be present in all the isogenic samples, they are filtered out based on their difference from the reference genome. The availability of a non-mutated reference sample is also not necessary if the mutated samples contain independently formed mutations.

Using the experimental dataset to establish reference test sets also presents a great advantage to currently used alternative approaches, which usually use some independent procedure to validate a small number of well-chosen SNVs [30, 31]. As this is usually done experimentally at a great cost of time and money, it is desirable to generate test sets in a more efficient manner. Using these test sets we demonstrated the optimisation of filtering parameters for diploid chromosomes. This way we were able to present filtering parameter settings suitable for different desired FPRs that can be used on datasets with no mutation-free control samples.

We designed IsoMut to be used in cases when multiple isogenic samples are available and unique mutations are sought. It is easily adapted to cases when the independence of mutations in certain sample subsets is not guaranteed; in these cases all but one of these sample subsets should be excluded from the analysis, while including several truly independent samples. Based on down-sampling an experimental dataset, we can recommend a minimum sample number of 14 and a minimum short-read sequence coverage of 15.

We strongly recommend sequencing negative control samples, and designed an adjustable approach that can be conveniently and quickly optimised for any specific dataset with such controls. This optimisation procedure can also be applied to non-diploid regions, where each level of ploidy should be treated separately.

Mutation analysis is widely used in the study of the DNA damaging effect of environmental substances and metabolism, DNA repair, cancer, and evolution. IsoMut can aid these studies by providing a solution for the accurate identification of SNVs and indels from pure isogenic samples such as cell clones or animal progeny regardless of the species and the available data on genomic polymorphisms.

## Additional files

Additional file 1: Table of samples. List of samples used in the study. Half the samples had wild type (‘WT’) and the other half’Mutant 1′ genotype. Samples underwent different types of mutagenic treatments, which are also indicated in the table. (PDF 221 kb)
Additional file 2: Detailed methods. A detailed description of methods for testing and mutation detection. (PDF 598 kb)
Additional file 3: Generating pileup files. Scripts and pipeline for pileup file generation. (HTML 223 kb)
Additional file 4: Generating SNV test sets. Workflow for the generation of SNV test cohorts. (HTML 390 kb)
Additional file 5: Verification and description of filtering parameters. A detailed verification of the chosen filtering parameters. (PDF 391 kb)
Additional file 6: Example run and tuning of IsoMut. An example run of IsoMut on a reduced dataset for easy testing. (HTML 1353 kb)
Additional file 7: Table of tested parameter settings. List of tested parameter settings with the resulting TPR and FPR values. (PDF 307 kb)
Additional file 8: Running VarScan 2 on our dataset. Computational details and results of running VarScan 2 on the described dataset. (HTML 408 kb)
Additional file 9: Running MuTect on our dataset with default settings. Computational details and results of running MuTect on the described dataset without the tuning of the LOD parameter. (HTML 675 kb)
Additional file 10: Comparison of IsoMut and MuTect. Comparison of false positives rates when running IsoMut versus running MuTect with a finely tuned LOD parameter. (HTML 373 kb)
Additional file 11: Runtime comparison of standard tools and IsoMut. A list of scripts and functions used to test the speed of standard mutation detection tools and IsoMut, using all resources of the available computer and using a single core only. (HTML 311 kb)

## Abbreviations

*ds_factor*: Down-sampling factor
FPR: False positive rate
indel: Insertion and/or deletion
LOH: Loss of heterozygosity
NGS: Next generation sequencing
*other_rnf_min*: Minimal threshold for the ratio of reference reads in the noisiest non-selected sample
*rnf*: Reference nucleotide frequency
*sample_cov_min*: Minimal threshold for the coverage of the selected sample
*sample_mut_freq_min*: Minimal threshold for the ratio of the most common type of non-reference reads in the investigated sample
SNP: Single nucleotide polymorphism
SNV: Single nucleotide variation
TPR: True positive rate
WT: Wild type
