# Supplementary material for: Fast and accurate mutation detection in whole genome sequences of multiple isogenic samples with IsoMut
Source: BMC Bioinformatics. 2017 Jan 31;18:73. doi: 10.1186/s12859-017-1492-4 (PMC5282906; doi:10.1186/s12859-017-1492-4)
Supplement: Additional file 1: — Table of samples. List of samples used in the study. Half the samples had wild type (‘WT’) and the other half’Mutant 1′ genotype. Samples underwent different types of mutagenic treatments, which are also indicated in the table. (PDF 221 kb) [file 12859_2017_1492_MOESM1_ESM.pdf]

Additional file 1 - Table of samples

Fast and accurate mutation detection in whole genome sequences of multiple isogenic samples with IsoMut

O. Pipek, D. Ribli, J. Molnár, Á. Póti, M. Krzystanek, A. Bodor, G. E. Tusnády, Z. Szallasi, I. Csabai, and D. Szüts

**Mutant 1**		**WT**	
**sample**	**treatment**	**treatment**	**sample**
**S16**	**starting clone**	**starting clone**	**S01**
**S17**	**strong mutagenic treatment**	**strong mutagenic treatment**	**S02**
S18	strong mutagenic treatment	strong mutagenic treatment	S03
S19	strong mutagenic treatment	strong mutagenic treatment	S04
S20	weak mutagenic treatment	weak mutagenic treatment	S05
S21	weak mutagenic treatment	weak mutagenic treatment	S06
S22	weak mutagenic treatment	weak mutagenic treatment	S07
S23	strong mutagenic treatment	strong mutagenic treatment	S08
S24	strong mutagenic treatment	strong mutagenic treatment	S09
S25	strong mutagenic treatment	strong mutagenic treatment	S10
S26	strong mutagenic treatment	strong mutagenic treatment	S11
**S27**	**identical control**	**identical control**	**S12**
S28	strong mutagenic treatment	strong mutagenic treatment	S13
S29	strong mutagenic treatment	strong mutagenic treatment	S14
**S30**	**identical control**	**identical control**	**S15**

bold: samples necessary for testing
