# Supplementary material for: Fast and accurate mutation detection in whole genome sequences of multiple isogenic samples with IsoMut
Source: BMC Bioinformatics. 2017 Jan 31;18:73. doi: 10.1186/s12859-017-1492-4 (PMC5282906; doi:10.1186/s12859-017-1492-4)
Supplement: Additional file 2: — Detailed methods. A detailed description of methods for testing and mutation detection. (PDF 598 kb) [file 12859_2017_1492_MOESM2_ESM.pdf]

Additional file 2 – Detailed methods

Fast and accurate mutation detection in whole genome sequences of multiple isogenic samples with IsoMut

O. Pipek, D. Ribli, J. Molnár, Á. Póti, M. Krzystanek, A. Bodor, G. E. Tusnády, Z. Szallasi, I. Csabai, and D. Szüts

Generating pileup files of all samples

After the generation of BAM files, further analysis was performed in a highly parallelized manner to reduce computation time. To achieve this, the genome was separated into approximately 100 pieces on which all additional filtering could be carried out separately.

For the testing phase, a joint pileup file of all investigated samples was created (using the samtools [S1] mpileup command) for time management purposes, as we needed to have access to this information repeatedly. However, in the IsoMut application available online [S2], the output of this intermediate step is not stored.

During the pileup generation the '-B' option of samtools was invoked. This disables the default algorithm which automatically lowers base quality values based on the probability that the given read has been misaligned in the vicinity of small indels [S3]. We skipped this step, because it would have reasonably slowed down the process, but in the post-processing phase, we analysed the pileups of the investigated samples without the '-B' option as well.

The default settings of the samtools mpileup command employ a base quality filter set to 13 (Phred-scaled probability). To investigate the possible effects of such filtering, we determined both the number of reasonably well-covered (cov ≥ 10) positions in a given sample and the number of 'clean in all samples' (strictly reference) positions in a 10^5 base long portion of the genome for the Mutant 1 genotype. Figure S2.1 suggests that a Q = 30 threshold would reduce the number of positions where samples differ from the reference genome due to sequencing noise, while still maintaining satisfying coverage. Thus the base quality filter option of '-Q 30' was applied.

The resulting output files consisted of 3 + 2N columns, the first three being the chromosome, position and reference base, while the rest the coverage and bases of each sample in the

dataset. Even though conventional pileup format contains the base qualities as well, in our case these were discarded for space saving purposes.

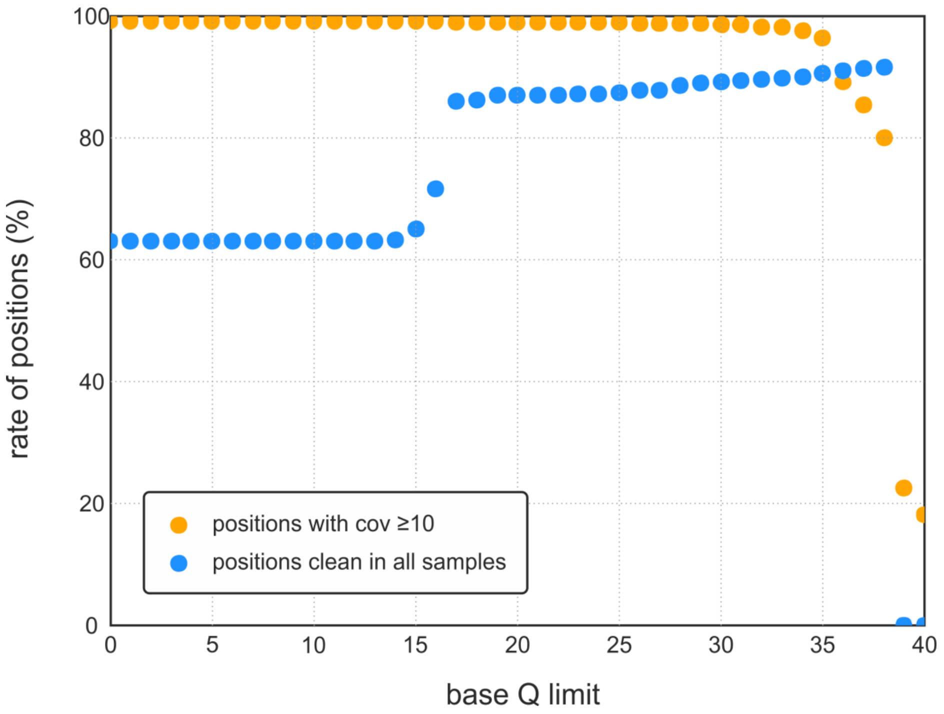

Figure S2.1. Validation of the '-Q 30' filter during pileup generation. The number of reasonably well-covered (cov ≥ 10) positions remains approximately constant, while the number of 'clean in all samples' (strictly reference) positions increases in the $0 < Q < 30$ region, but both rapidly decrease where $Q > 35$. Thus a $Q = 30$ threshold value was chosen for base quality filtering.

For SNV detection, all genomic positions were discarded where any indels appeared in at least one sample. Also, potentially uninteresting positions were skipped as well, that is where none of the samples differ from the reference genome. As mutations are identified with an alternate base frequency (*sample_mut_freq*) cut-off of around 0.3, determined in a later step, it is prudent to use a less strict pre-filter on its complementary variable, the reference nucleotide frequency (*rnf*) to reduce the pileup file size. Thus we discarded all positions where every sample shows the reference sequence in at least 90% of the reads ($rnf \geq 0.9$). This removes sequencing errors at coverage > 10 when they are present in one read only. With this step we were able to reduce output file sizes to only 1% of their original size, which allows for faster computation throughout the following stages.

‘Only indel’ pileup files were created similarly from the BAM files, which included those genomic positions only where in at least one sample either the frequency of deletions or insertions reached the 0.1 level.

For both types of pileup generation scripts, see Supplementary file 3.

Calculation of TRP and FPR values

Throughout the testing, true positive mutations (TP_X, where *X* denotes the genotype) are SNVs detected in the starting clones (when other samples of the same genotype are excluded) that are also present in the given heterozygous test set. To determine the value of TPR, these genotype-specific values were normalized by the total number of heterozygous test set positions (N_X^{het}) and then averaged between the genotypes.

$$TPR = mean\left(\frac{TP_{WT}}{N_{WT}^{het}}, \frac{TP_{Mutant\ 1}}{N_{Mutant\ 1}^{het}}\right)$$

False positive mutations have three different sources, one of which are SNVs found in the starting clones that are not in the respective test set (B_X). These are normalized with the number of true negatives that is equal to the total genomic length of the investigated chromosomes (L_{inv_chrom}) minus the number of positions in the heterozygous (N_X^{het}) test set. SNVs detected in any of the identical samples (C_{S12}, C_{S15} and C_{S27}, C_{S30}) are also false positives. (Any SNV found in a sample that has an identical twin is a false positive as these mutations should be ‘cancelled out’.) In this case, the number of true negatives is simply L_{inv_chrom}.

To assess the effect of having very few available samples from a specific cell line, that are not identical but still share mutations, the SNVs found in the Mutant 1 starting clone were counted ($D_{Mutant\ 1}$), while excluding all but one other Mutant 1 samples from the analysis. Similarly, D_{WT} was calculated for the WT starting clone. Both of these are false positives as well, as no treatment-induced mutations are expected to be present in any of the starting clones. These values were also divided by L_{inv_chrom}. The FPR was defined as the mean of the above eight numbers.

$$FPR =$$

$$= mean\left(\frac{B_{WT}}{L_{inv\_chrom} - N_{WT}^{het}}, \frac{B_{Mutant\ 1}}{L_{inv\_chrom} - N_{Mutant\ 1}^{het}}, \frac{C_{S12}}{L_{inv\_chrom}}, \frac{C_{S15}}{L_{inv\_chrom}}, \frac{C_{S27}}{L_{inv\_chrom}}, \frac{C_{S30}}{L_{inv\_chrom}}, \frac{D_{WT}}{L_{inv\_chrom}}, \frac{D_{Mutant\ 1}}{L_{inv\_chrom}}\right)$$

Assessing the effects on smaller datasets

To simultaneously change the number of samples in the two investigated genotypes, samples were always excluded in (WT, Mutant 1) pairs for the generation of testing groups. Both the test set generation and the testing procedure were carried out on these reduced datasets for the parameter settings of the inset in Figure 3A.

For $8 < n < 30$ each point on Figure 3D represents the mean value of TPRs and FPRs for three randomly chosen datasets with the given *n*, error bars showing the standard deviation of the data. As we had altogether 30 available samples, 8 of which was needed for the optimization process, the $n = 30$ and $n = 8$ groups could only be selected one way. Accordingly, the points belonging to these sets of samples are not averages, but single TPR and FPR values.

Assessing the effects of decreased sample coverage

For the generation of Figure 3E the Mutant 1 starting clone was manually down-sampled by *ds_factors* of 0.7, 0.6 and 0.5. For this, a (*ds_factor* · coverage) sized portion of the original bases was chosen randomly in each genomic position. If the remaining bases still met the filtering conditions set by *sample_cov_min* and *sample_mut_freq_min*, the position was categorized as an SNV. TPR and FPR were calculated similarly as described above, this time only using the SNVs found in the Mutant 1 starting clone that are also in the Mutant 1 test set as true positives. Likewise, false positive mutations were the ones found in the Mutant 1 starting clone that did not belong to the Mutant 1 test set and the ones found in the Mutant 1 starting clone while including another Mutant 1 sample in the analysis. As the quality values depend on the actual nucleotide bases that were kept in a given position, which were chosen randomly, we carried out the testing procedure for each *ds_factor* three times. Data points on Figure 3E are averages of these three values with the standard deviation as error bars.

Post-processing SNV and indel candidates

While during the joint pileup generation we applied the '–B' option of the samtools mpileup command, after a set of candidate SNVs has been established for a given sample, we reran the mpileup script locally for that sample only, now without the –B option and recalculated relevant parameters (*sample_mut_freq*, *sample_cov*). The BAQ recalibration used by samtools decreases the quality of the bases around an indel, which are likely to have been aligned falsely. Thus by invoking this default filtering for the investigated sample, we aim to get rid of false positive mutations in the vicinity of indels. This practice corresponds to the idea that in the mutated sample we wish to be very sure of the mutation (meaning that the number of supporting reads should be high enough even after filtering out noise), while in other samples all sources of noise should be included when invoking the *other_rnf_min* threshold.

For indels, a crucial post-processing step is to filter out any candidate mutations whenever they appear in the 'near vicinity' of another 'suspicious' position. A position was deemed suspicious if the indel frequency in one of the samples reached the 0.2 limit, suggesting (but not reliably proving) a possible mutation. The near vicinity of such a position was defined as a 10-base radius neighbourhood in both directions. This practice was necessitated by a common problem while aligning reads, namely that alignment tools tend to map short indels to slightly different positions in each sample. Wherever a germline indel is present, this would result in a range in the genome where individual indels seemingly appear clustered in multiple samples. Using a relatively small neighbourhood size this effect can be eliminated without discarding real, unique mutations.

Estimation of indel detection performance

To get an estimate of the TPR and FPR values for indels, first the indel pileup files were analysed for the establishment of indel test sets the same way as SNV ~~test~~ tests were created. The resulting mean indel frequency plots of the two genotypes can be seen on Figure S2.2. Indel test sets are significantly smaller than their SNV counterparts, having altogether less than 400 positions, that is about 10% of the genotype-specific SNV groups. Thorough optimization is thus problematic for indels, lacking a sufficiently large set of control positions for statistical analysis, thus we used the same testing procedure as described for SNVs, but

only on pre-defined parameter settings already tested for SNVs (see Figure 3A). The results can be seen on Table S2.1.

According to Table S2.1, using the same filtering parameters for indels as for SNVs works well for keeping FPRs low, but results in decreased TPRs. If the main objective is to strictly detect individual indels in the samples, applying the same settings for indel and SNV detection gives satisfactory results. Whenever the sensitivity of the indel detection is crucial, weaker filtering parameters should be chosen.

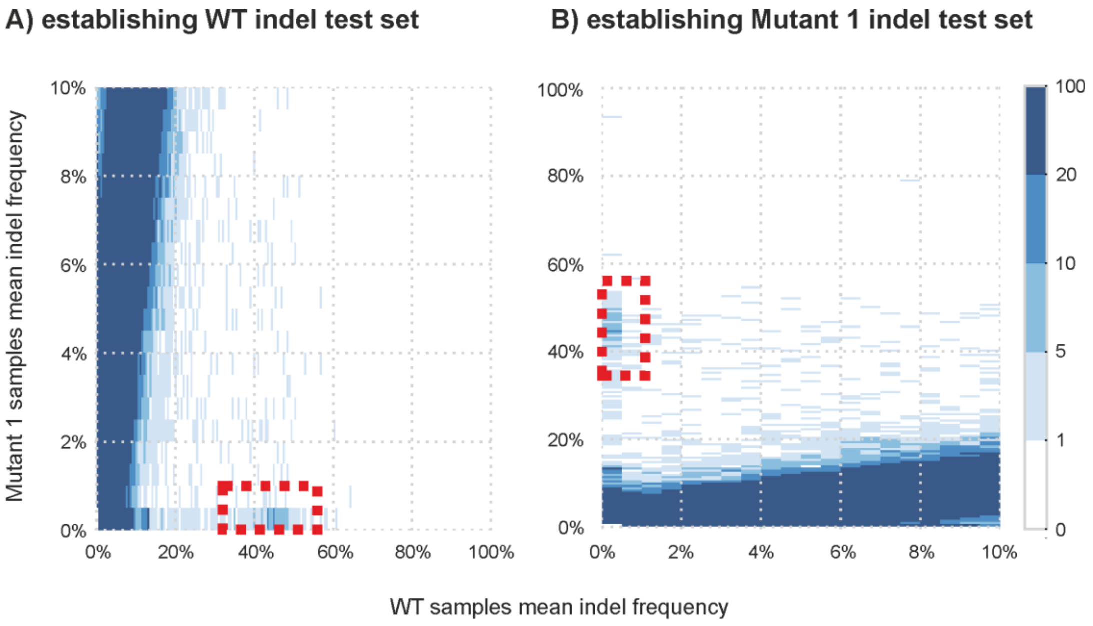

Figure S2.2. Genotype-specific indel test sets for WT and Mutant 1 samples.

sample_mut_freq_min	***other_rnf_min***	***sample_cov_min***	**FPR (10^{-9})**	**TPR (%)**
0.5	0.93	7.0	14.30	46.82
0.34	0.96	7.0	21.56	64.35
0.35	0.93	7.0	27.06	68.77
0.34	0.92	7.0	31.68	70.63
0.31	0.93	7.0	29.04	75.90
0.31	0.92	7.0	33.00	76.87
0.3	0.92	7.0	35.86	78.03
0.3	0.9	7.0	42.46	79.17

Table S2.1 Testing the settings included in the inset of Figure 3A for indel detection. For each setting, the calculated FPR and TPR values are shown.

Calculation of the S score value

The *S* value is related to the *p* probability of falsely categorizing a genomic position as a unique point mutation in the noisiest sample, given the short read sequences of the two noisiest samples. The probability *p* can be calculated using Fisher's exact test, for which IsoMut uses the script available online at [S4]. A two-by-two contingency table of the two noisiest samples (Table S2.2) can be generated by determining the number n_R of reference and the number n_{NR} of most common non-reference bases at the given position. Let the index 1 denote the noisiest and 2 the second noisiest sample.

	**1**	**2**
**reference**	${n_R}^1$	${n_R}^2$
**most common non-reference**	${n_{NR}}^1$	${n_{NR}}^2$

Table S2.2. The contingency table of the two noisiest samples in the given genomic position

Let us assume as a null hypothesis that the distribution of bases in the two samples is the same, thus there is no unique mutation in the noisiest sample. The probability *p* gives the likelihood of observing the above data in such a case and is calculated using the following formula:

$$p = \frac{(n_R^1 + n_R^2)!\,(n_{NR}^1 + n_{NR}^2)!\,(n_R^1 + n_{NR}^1)!\,(n_R^2 + n_{NR}^2)!}{n_R^1!\,n_R^2!\,n_{NR}^1!\,n_{NR}^2!\,(n_R^1 + n_R^2 + n_{NR}^1 + n_{NR}^2)!}$$

where ! denotes the factorial operator. Thus a low *p* value suggests that it is unlikely that the two investigated samples have the same base-distribution, making it likely that the noisiest sample indeed has a unique mutation in the given position. The *S* score value used in IsoMut is calculated as the negative logarithm of *p*, resulting in high *S* for likely and low *S* for unlikely mutations.

$$S = -\log p$$

References

[S1] Li H., Handsaker B., Wysoker A., Fennell T., Ruan J., Homer N., Marth G., Abecasis G., Durbin R. and 1000 Genome Project Data Processing Subgroup (2009) The Sequence alignment/map (SAM) format and SAMtools. Bioinformatics, 25, 2078-9. [PMID: 19505943]

[S2] https://github.com/riblidezso/isomut

[S3] Li H. Improving SNP discovery by base alignment quality. Bioinformatics. 2011;27(8):1157-1158. doi:10.1093/bioinformatics/btr076.

[S4] https://github.com/chrchang/stats
