# Supplementary material for: Fast and accurate mutation detection in whole genome sequences of multiple isogenic samples with IsoMut
Source: BMC Bioinformatics. 2017 Jan 31;18:73. doi: 10.1186/s12859-017-1492-4 (PMC5282906; doi:10.1186/s12859-017-1492-4)
Supplement: Additional file 3: — Generating pileup files. Scripts and pipeline for pileup file generation. (HTML 223 kb) [file 12859_2017_1492_MOESM3_ESM.html]

Pileup\_generation\_scripts


# Additional file 3 - Generation of pileup files¶

### Fast and accurate mutation detection in whole genome sequences of multiple isogenic samples with IsoMut¶

##### O. Pipek, D. Ribli, J. Molnár, Á. Póti, M. Krzystanek, A. Bodor, G. E. Tusnády, Z. Szallasi, I. Csabai, and D. Szüts¶

---

# Usage:¶

## Notes:¶

- The following ipython notebook uses the Slurm Workload Manager (http://slurm.schedmd.com/) to parallelly run scripts, thus the syntax is adjusted for this special case.
- Whenever a different workload manager is preferred, the cell marked with '¤¤¤' should be adjusted accordingly. The other parts of the code can be used without significant modifications.

- The following tools have to be installed:
  - Biopython (http://biopython.org/wiki/Main\_Page)
  - samtools (http://samtools.sourceforge.net/)
  - awk, grep, sed

## Method:¶

1. adjust input/output directories and sample info file
2. run each cell individually by hitting Shift+Enter

---

---

# Technical aspects:¶

### Parallelization¶

- the genome is cut into several smaller pieces (the approximate number of blocks can be predefined by the user)
- the resulting pieces are processed in a parallel manner

### Pileup generation¶

- samtools mpileup on all available samples

### Filtering¶

- intially with grep, awk, sed
- later steps are done in python

---

---

# Creating filtered mpileup files from the available BAM files¶

### Discarded positions in case of SNVs:¶

- insertion or deletion appears in the position
- reference nucleotide frequency > 0.9 for all samples (possibly due to noise)

### Discarded positions in case of indels:¶

- both insertion and deletion frequency below 0.1 in all samples

### Discarded bases:¶

- Q < 30 (base quality filtering option of *samtools mpileup*)

## Output format:¶

- modified mpileup format for all samples: base quality columns are discarded

### Notes:¶

- '-B' option reduces runtime of the script (no base quality recalculation in the vicinity of indels)

---

### Setting mutation type:¶

Please adjust accordingly.

In [9]:

```
SNV = True
indel = True
```

### Input and output directories and files:¶

#### Current directory:¶

In [10]:

```
import os
current_dir = os.getcwd()
```

#### Directories and paths of necessary tools:¶

Please adjust accordingly.

In [11]:

```
biopython_dir = '/home/ribli/tools/biopython-1.64/'
samtools_dir='/nagyvinyok/adat87/home/orsi/DT40/tools/samtools-1.2/'
```

#### Location of reference genome (fasta file):¶

Please adjust accordingly.

In [12]:

```
ref_fasta= '/nagyvinyok/adat87/home/ribli/input/index/gallus/Gallus_gallus.Galgal4.74.dna.toplevel.fa'
```

#### Directory of BAM files, extended name of BAM files:¶

Please adjust accordingly.

In [13]:

```
bam_dir = '/nagyvinyok/adat83/sotejedlik/orsi/bam_all_links_methodpaper/'
bam_name_extension = '_RMdup_picard_realign.bam'
```

#### Output directory for mpileup files:¶

Please adjust accordingly.

In [14]:

```
if (SNV):
    mpileup_output_SNV_dir = '/'.join(current_dir.split('/')[:-1]) + '/mpileup_SNV/'
if (indel):
    mpileup_output_indel_dir = '/'.join(current_dir.split('/')[:-1]) + '/mpileup_indel/'
```

#### Location of sample info file:¶

Please adjust both the file and location accordingly.

In [15]:

```
sample_info = '/'.join(current_dir.split('/')[:-1]) + '/sample_info/sample_info_file.txt'
```

#### Location of error files:¶

Please adjust accordingly.

In [16]:

```
slurm_dir = '/'.join(current_dir.split('/')[:-1]) + '/slurm_out/'
```

---

### Calculating the blocks for parallelization:¶

#### Approximate number of blocks:¶

(The actual number will be greater, please set a minimal value.)

In [17]:

```
BLOCKNO=100
```

#### Calculating blocks:¶

In [18]:

```
import sys
sys.path.append(biopython_dir)

from Bio import SeqIO
from Bio.Seq import Seq

##################################################################################################################
# determining genome length
##################################################################################################################

chromDict=dict()
fullLeng=0

# parsing fasta file
for seqin in SeqIO.parse(ref_fasta,"fasta"):
    # scaffolds and mitochondrium are not used for analysis
        if (len(seqin.id) < 3 and seqin.id!='MT' ):
            # save lengths
            chromDict[seqin.id]=len(seqin.seq)
            fullLeng+=len(seqin.seq)

##################################################################################################################
# setting the largest possible block size
##################################################################################################################
            
BLOCKSIZE=(fullLeng/BLOCKNO)

##################################################################################################################
# separating chromosomes into blocks
##################################################################################################################

blocks=[]
for chrom in chromDict.keys():
	pointer=0
	while (pointer < chromDict[chrom]):
		blockSize=min(BLOCKSIZE,chromDict[chrom]-pointer)
	        blocks.append([chrom,pointer,pointer+blockSize])
	        pointer += blockSize
```

---

### ¤¤¤ Running the mpileup generation script parallelly for the blocks in slurm¶

Please, adjust to the specific syntax of the used workload manager.

In [19]:

```
if (SNV):
    import subprocess
    for block in blocks:
        try:
            a = subprocess.call([ 'sbatch', '--mem',str(1000), '-C', 'jimgray88', '--output=' + slurm_dir + block[0]+'-'+str(block[1])+'-'+str(block[2]) + '_SNVpup_slurm-out.txt', './mpileup_generation_SNV.py', block[0],str(block[1]),str(block[2]), mpileup_output_SNV_dir, samtools_dir, ref_fasta, sample_info, bam_dir, bam_name_extension],stderr=subprocess.STDOUT),
        except subprocess.CalledProcessError, e:
            print e.output,
```

In [20]:

```
if (indel):
    import subprocess
    for block in blocks:
        try:
            a = subprocess.call([ 'sbatch', '--mem',str(1000), '-C', 'jimgray88', '--output=' + slurm_dir + block[0]+'-'+str(block[1])+'-'+str(block[2]) + '_indelpup_slurm-out.txt', './mpileup_generation_indel.py', block[0],str(block[1]),str(block[2]), mpileup_output_indel_dir, samtools_dir, ref_fasta, sample_info, bam_dir, bam_name_extension],stderr=subprocess.STDOUT),
        except subprocess.CalledProcessError, e:
            print e.output,
```

### Clearing error directory:¶

In [21]:

```
import shutil
for error_file in os.listdir(slurm_dir):
    file_path = os.path.join(slurm_dir, error_file)
    if os.path.isfile(file_path):
        os.unlink(file_path)
```

---


---

### Python codes for analysis:¶

In [1]:

```
%%writefile mpileup_generation_SNV.py
#!/usr/bin/python

##################################################################################################################
# importing modules
##################################################################################################################

import os
import sys
import subprocess
import fnmatch
import re
import numpy as np

##################################################################################################################
# data from commandline
##################################################################################################################

chrom=sys.argv[1]
posfrom=sys.argv[2]
posto='-'+sys.argv[3]
output_dir = sys.argv[4]
samtools = sys.argv[5] + 'samtools'
ref_fasta = sys.argv[6]
sample_info_file = sys.argv[7]
bam_dir = sys.argv[8]
bam_name_ext = sys.argv[9]

##################################################################################################################
# loading sample info file
##################################################################################################################

samples=[]
genotypes=[]
treatments=[]
si_file = open(sample_info_file)
for nextline in iter(si_file.readline, b''):
    samples.append(nextline.strip('\n').split('\t')[0])
    genotypes.append(nextline.strip('\n').split('\t')[1])
    treatments.append(nextline.strip('\n').split('\t')[2])
si_file.close()

##################################################################################################################
# setting samtools flags
##################################################################################################################

flags=" -Q30 -B "

##################################################################################################################
# output file name
##################################################################################################################

output_fname= str(chrom) + '-' + str(posfrom) +  str(posto) + '.pup'

##################################################################################################################
# generating samtools mpileup command
##################################################################################################################

cmd_mpileup = samtools + ' mpileup ' + flags + ' -f ' + ref_fasta
cmd_mpileup += ' -r ' + str(chrom) +':'+ str(posfrom) +  str(posto) + ' ' 
cmd_mpileup += ' '.join([bam_dir+x+bam_name_ext for x in samples]) + ' | '

##################################################################################################################
# filtering steps
##################################################################################################################

#### base qualiies are not stored 
cmd_q_filt= ' awk \' BEGIN{FS=\"\t\"} { print $1,$2,$3'
for i in xrange(len(samples)):
    cmd_q_filt+=',$'+str(4+i*3)+',$'+str(5+i*3)
cmd_q_filt+='} \' | '

#### discarding positions where insersions or deletions are present
cmd_del_ins_filt= " grep -v  [\*\+\-] | "

#### deleting "." mapping quality to avoid confusion
cmd_first_base_filt= " sed  's/\^.//g' " 

##################################################################################################################
# creating full command and executing with further filtering in python
##################################################################################################################

cmd_full= cmd_mpileup + cmd_q_filt + cmd_del_ins_filt
cmd_full += cmd_first_base_filt 

output_f=open(output_dir + output_fname,'w')
from subprocess import Popen, PIPE
p = Popen(cmd_full, stdout=PIPE, bufsize=1,executable='/bin/bash',shell=True)
with p.stdout:
    for line in iter(p.stdout.readline, b''):
        linelist=line.upper().split(' ')
        covs=np.array(map(int,linelist[3::2]),dtype=np.int32)
        bases=linelist[4::2]

        # counting reference bases for each sample
        ref_count=[]
        for i in xrange(len(bases)):
            ref_count.append(len(re.findall('[\.\,]',bases[i]))) 
        ref_count=np.array(ref_count,dtype=np.double)

        # calculating reference nucleotide frequency
        ref_freq=np.zeros(len(ref_count),dtype=np.double)
        ref_freq[covs !=0]=ref_count[covs !=0]/covs[covs != 0]
        ref_freq_id=np.argsort(ref_freq)

        # discarding relatively clean positions
        if(ref_freq[ref_freq_id[0]] >=  0.9):
            continue

        # print pileup
        output_f.write(line)
output_f.close()
```

```
Writing mpileup_generation_SNV.py
```

In [8]:

```
%%writefile mpileup_generation_indel.py
#!/usr/bin/python

##################################################################################################################
# importing modules
##################################################################################################################

import os
import sys
import subprocess
import fnmatch
import re
import numpy as np

##################################################################################################################
# data from commandline
##################################################################################################################

chrom=sys.argv[1]
posfrom=sys.argv[2]
posto='-'+sys.argv[3]
output_dir = sys.argv[4]
samtools = sys.argv[5] + 'samtools'
ref_fasta = sys.argv[6]
sample_info_file = sys.argv[7]
bam_dir = sys.argv[8]
bam_name_ext = sys.argv[9]

##################################################################################################################
# loading sample info file
##################################################################################################################

samples=[]
genotypes=[]
treatments=[]
si_file = open(sample_info_file)
for nextline in iter(si_file.readline, b''):
    samples.append(nextline.strip('\n').split('\t')[0])
    genotypes.append(nextline.strip('\n').split('\t')[1])
    treatments.append(nextline.strip('\n').split('\t')[2])
si_file.close()

##################################################################################################################
# setting samtools flags
##################################################################################################################

flags=" -Q30 -B "

##################################################################################################################
# output file name
##################################################################################################################

output_fname= str(chrom) + '-' + str(posfrom) +  str(posto) + '.pup'

##################################################################################################################
# generating samtools mpileup command
##################################################################################################################

cmd_mpileup = samtools + ' mpileup ' + flags + ' -f ' + ref_fasta
cmd_mpileup += ' -r ' + str(chrom) +':'+ str(posfrom) +  str(posto) + ' ' 
cmd_mpileup += ' '.join([bam_dir+x+bam_name_ext for x in samples]) + ' | '

##################################################################################################################
# filtering steps
##################################################################################################################

#### base qualiies are not stored 
cmd_q_filt= ' awk \' BEGIN{FS=\"\t\"} { print $1,$2,$3'
for i in xrange(len(samples)):
    cmd_q_filt+=',$'+str(4+i*3)+',$'+str(5+i*3)
cmd_q_filt+='} \' | '

#### discarding positions where insersions or deletions are not present at all
cmd_del_ins_filt= " grep [\+\-] | "

#### deleting "." mapping quality to avoid confusion
cmd_first_base_filt= " sed  's/\^.//g' " 

##################################################################################################################
# creating full command and executing with further filtering in python
##################################################################################################################

cmd_full= cmd_mpileup + cmd_q_filt + cmd_del_ins_filt
cmd_full += cmd_first_base_filt 

output_f=open(output_dir + output_fname,'w')
from subprocess import Popen, PIPE
p = Popen(cmd_full, stdout=PIPE, bufsize=1,executable='/bin/bash',shell=True)
with p.stdout:
    for line in iter(p.stdout.readline, b''):
        linelist=line.upper().split(' ')
        covs=np.array(map(int,linelist[3::2]),dtype=np.int32)
        bases=linelist[4::2]

        # counting insertions & deletions for each sample
        insertion=[]
        deletion=[]
        for i in xrange(len(bases)):
            insertion.append(len(re.findall('[\+]',bases[i])))
            deletion.append(len(re.findall('[\-]',bases[i])))
        insertion=np.array(insertion,dtype=np.double)
        deletion=np.array(deletion,dtype=np.double)

        # calculating insertions and deletion frequencies

        inFreq=np.zeros(len(insertion),dtype=np.double)
        delFreq=np.zeros(len(deletion),dtype=np.double)
        inFreq[covs !=0]=insertion[covs !=0]/covs[covs != 0]
        delFreq[covs !=0]=deletion[covs !=0]/covs[covs != 0]
        inFreq_id=np.argsort(inFreq)
        delFreq_id=np.argsort(delFreq)

        # discarding relatively clean positions
        if ( (inFreq[inFreq_id[-1]] <  0.1) and (delFreq[delFreq_id[-1]] <  0.1) ) :
            continue

        # print pileup
        output_f.write(line)
output_f.close()
```

```
Overwriting mpileup_generation_indel.py
```
