# Supplementary material for: Fast and accurate mutation detection in whole genome sequences of multiple isogenic samples with IsoMut
Source: BMC Bioinformatics. 2017 Jan 31;18:73. doi: 10.1186/s12859-017-1492-4 (PMC5282906; doi:10.1186/s12859-017-1492-4)
Supplement: Additional file 4: — Generating SNV test sets. Workflow for the generation of SNV test cohorts. (HTML 390 kb) [file 12859_2017_1492_MOESM4_ESM.html]

testset\_generation\_SNV


# Additional file 4 - Generation of SNV test sets¶

### Fast and accurate mutation detection in whole genome sequences of multiple isogenic samples with IsoMut¶

##### O. Pipek, D. Ribli, J. Molnár, Á. Póti, M. Krzystanek, A. Bodor, G. E. Tusnády, Z. Szallasi, I. Csabai, and D. Szüts¶

---

# Usage:¶

## Notes:¶

- The following ipython notebook uses the Slurm Workload Manager (http://slurm.schedmd.com/) to parallelly run scripts, thus the syntax is adjusted for this special case.
- Whenever a different workload manager is preferred, the cell marked with '¤¤¤' should be adjusted accordingly. The other parts of the code can be used without significant modifications.

**This notebook file uses the previously generated pileup files as its input. Please, run the "Pileup\_generation\_scripts.ipynb" notebook file first.**

## Method:¶

1. adjust source directory and sample info file
2. run each cell individually by hitting Shift+Enter
   - this notebook file uses pileup files as its input, please make sure to **run the Pileup\_generation\_scripts.ipynb file first**!

---

---

# Technical aspects:¶

### Parallelization¶

- pileup files were created on smaller blocks of the genome
- the processing of these pileup files can be done parallelly

---

---

# Establishing SNV test sets:¶

1. Calculating the mean reference nucleotide frequency (mean rnf) of samples in the two different genotypes (WT and Mutant 1).
2. Plotting a heatmap of the distribution of the positions based on the two mean rnfs calculated.
3. Identifying test sets:
   - WT test set: around [50%, 100%]
   - Mutant 1 test set: around [100%, 50%]
4. Collecting positions in the respective clusters.

---

### Input and output directories and files:¶

#### Current directory:¶

In [35]:

```
import os
current_dir = os.getcwd()
```

#### Directory of previously generated SNV pileup files:¶

Please adjust accordingly.

In [36]:

```
mpileup_SNV_input_dir = '/'.join(current_dir.split('/')[:-1]) + '/mpileup_SNV/'
```

#### Directory of heatmap output files:¶

In [37]:

```
heatmap_output_dir = '/'.join(current_dir.split('/')[:-1]) + '/test_sets/heatmap_temp/'
```

#### Output directory for lists of test set positions:¶

In [38]:

```
WT_test_set_output_dir = '/'.join(current_dir.split('/')[:-1]) + '/test_sets/WT_test_set/'
Mutant1_test_set_output_dir = '/'.join(current_dir.split('/')[:-1]) + '/test_sets/Mutant1_test_set/'
```

#### Location of sample info file:¶

Please adjust both the file and location accordingly.

In [39]:

```
sample_info = '/'.join(current_dir.split('/')[:-1]) + '/sample_info/sample_info_file.txt'
```

#### Location of error files:¶

In [40]:

```
slurm_dir = '/'.join(current_dir.split('/')[:-1]) + '/slurm_out/'
```

---

### Other input arguments:¶

#### List of excluded samples:¶

In [41]:

```
excluded_samples = []
```

#### List of excluded chromosomes:¶

Identifying test sets is most straightforward on diploid chromosomes only.

In [42]:

```
excluded_chromosomes = ['2', '21', '24', '32', 'W', 'Z', 'MT']
```

#### Would you like to save figures?¶

In [43]:

```
save_figures = False
```

#### If yes: output figure directory:¶

In [44]:

```
if (save_figures):
    figure_dir = '/'.join(current_dir.split('/')[:-1]) + '/test_sets/figures/'
    figure_format = 'svg'
```

---

### ¤¤¤ Running the heatmap data generation script parallelly for the blocks in slurm¶

Please, adjust to the specific syntax of the used workload manager.

In [45]:

```
import subprocess
import os

for filename in os.listdir(mpileup_SNV_input_dir):
	if (filename.split("-")[0] in excluded_chromosomes):
		continue
	try:
		a = subprocess.call([ 'sbatch', '--mem',str(1000), '--exclude=jimgray84', '--output=' + slurm_dir + filename.split(".")[0] + '_slurm_out.txt', './test_set_generation_SNV.py' , filename, mpileup_SNV_input_dir, heatmap_output_dir, sample_info, str(excluded_samples)],stderr=subprocess.STDOUT),
	except subprocess.CalledProcessError, e:
		print e.output,
```

---

### Plotting mean rnf distribution of genomic positions:¶

#### Setting borders of test set clusters:¶

In [46]:

```
# WT test set

wt_test_set_wt_lower = 0.4
wt_test_set_wt_upper = 0.6
wt_test_set_mutant1_lower = 0.98
wt_test_set_mutant1_upper = 1

# Mutant 1 test set

mutant1_test_set_wt_lower = 0.98
mutant1_test_set_wt_upper = 1
mutant1_test_set_mutant1_lower = 0.4
mutant1_test_set_mutant1_upper = 0.6
```

In [47]:

```
##################################################################################################################
# importing modules
##################################################################################################################

import os
import numpy as np
import time
from matplotlib.colors import LogNorm
import matplotlib as mpl
import pandas as pd 
import matplotlib.pyplot as plt
from pylab import *
%matplotlib inline

##################################################################################################################
# loading data from heatmap output files
##################################################################################################################

m=pd.read_csv(heatmap_output_dir+os.listdir(heatmap_output_dir)[0],sep=' ',header=None)
for filename in os.listdir(heatmap_output_dir):
    try:
        m+=pd.read_csv(heatmap_output_dir+filename,sep=' ',header=None)
    except:
        pass

##################################################################################################################
# plotting results
##################################################################################################################

fig,ax=plt.subplots()

###### set image size
fig.set_size_inches(16,16)

###### colormap settings
cmap = plt.cm.Greys
cmaplist = [cmap(i) for i in range(cmap.N)]
cmaplist[0] = (1.0,1.0,1.0,1.0)
cmap = cmap.from_list('Custom cmap', cmaplist, cmap.N)
bounds = [0,1,5,10,20,100]
norm = mpl.colors.BoundaryNorm(bounds, cmap.N)

###### showing the image
cax = ax.imshow(m,interpolation='none',norm=norm,cmap=cmap,alpha=0.45,origin='lower')

###### figure properties

# colorbar
cbar=fig.colorbar(cax,shrink=0.8)
cbar.outline.set_edgecolor('lightgrey')

# grid
ax.grid(True,c='lightgrey',lw=1,linestyle='dotted')
ax.set_frame_on(False)

# tics
tics=ax.xaxis.set_ticks(np.linspace(0,200,6))
labs=ax.set_xticklabels(['0%','20%','40%','60%','80%','100%'], rotation='horizontal')
tics=ax.yaxis.set_ticks(np.linspace(0,200,6))
labs=ax.set_yticklabels(['0%','20%','40%','60%','80%','100%'], rotation='horizontal')
ax.xaxis.set_tick_params(size=0)
ax.yaxis.set_tick_params(size=0)

# limits
ax.set_xlim(-1,201)
ax.set_ylim(-1,201)

# font size
mpl.rcParams['font.size']=14.0

# axis labels
ax.set_xlabel('\nWT samples mean rnf')
ax.set_ylabel('Mutant 1 samples mean rnf\n')


##################################################################################################################
# Mutant 1 test set inset
##################################################################################################################

###### colormap settings
cmap = plt.cm.Blues
cmaplist = [cmap(i) for i in range(cmap.N)]
cmaplist[0] = (1.0,1.0,1.0,1.0)
cmap = cmap.from_list('Custom cmap', cmaplist, cmap.N)
bounds = [0,1,5,10,20,100]
norm = mpl.colors.BoundaryNorm(bounds, cmap.N)

###### showing inset image
ax_inset=fig.add_axes([0.33,0.25,0.2,0.2])
ax_inset.imshow(np.array(m)[:,180:],interpolation='none',extent=[180,200,0,200], aspect=0.1,alpha=1,origin='lower',cmap=cmap,norm=norm)

###### inset properties

# grid
ax_inset.grid(True,c='lightgrey',lw=1,linestyle='dotted')
ax_inset.set_frame_on(False)

# tics
ax_inset.xaxis.set_ticks(np.linspace(180,200,6))
ax_inset.yaxis.set_ticks(np.linspace(0,200,6))
ax_inset.set_yticklabels(['0%','20%','40%','60%','80%','100%'], rotation='horizontal', size=9)
ax_inset.set_xticklabels(['90%','92%','94%','96%','98%','100%'], rotation='horizontal', size=9)
ax_inset.xaxis.set_tick_params(size=0)
ax_inset.yaxis.set_tick_params(size=0)

# limits
ax_inset.set_xlim(180,201)
ax_inset.set_ylim(0,205)

# cluster borders
rect=plt.Rectangle((mutant1_test_set_wt_lower*200,mutant1_test_set_mutant1_lower*200),(mutant1_test_set_wt_upper-mutant1_test_set_wt_lower)*200,(mutant1_test_set_mutant1_upper-mutant1_test_set_mutant1_lower)*200, fc='none',ec='r',lw=3.5, linestyle='dashed')
ax_inset.add_patch(rect)

# inset border
rect=plt.Rectangle((180,1),20,199, ec='k', fc='none', lw=1)
ax_inset.add_patch(rect)

# inset background
rect=plt.Rectangle((180,1),20,199, fc='#002591', alpha=0.1, lw=1)
ax_inset.add_patch(rect)

##################################################################################################################
# WT 1 test set inset
##################################################################################################################

###### colormap settings
cmap = plt.cm.Greens
cmaplist = [cmap(i) for i in range(cmap.N)]
cmaplist[0] = (1.0,1.0,1.0,1.0)
cmap = cmap.from_list('Custom cmap', cmaplist, cmap.N)
bounds = [0,1,5,10,20,100]
norm = mpl.colors.BoundaryNorm(bounds, cmap.N)

###### showing inset image
ax_inset2=fig.add_axes([0.33,0.5,0.2,0.2])
ax_inset2.imshow(np.array(m)[180:,:],interpolation='none',extent=[0,200,180,200], aspect=10,alpha=1,origin='lower',cmap=cmap,norm=norm)

###### inset properies

# grid
ax_inset2.grid(True,c='lightgrey',lw=1,linestyle='dotted')
ax_inset2.set_frame_on(False)

# tics
ax_inset2.yaxis.set_ticks(np.linspace(180,200,6))
ax_inset2.xaxis.set_ticks(np.linspace(0,200,6))
ax_inset2.set_xticklabels(['0%','20%','40%','60%','80%','100%'], rotation='horizontal', size=9)
ax_inset2.set_yticklabels(['90%','92%','94%','96%','98%','100%'], rotation='horizontal', size=9)
ax_inset2.xaxis.set_tick_params(size=0)
ax_inset2.yaxis.set_tick_params(size=0)

# limits
ax_inset2.set_ylim(180,201)
ax_inset2.set_xlim(0,205)

# cluster borders
rect=plt.Rectangle((wt_test_set_wt_lower*200,wt_test_set_mutant1_lower*200),(wt_test_set_wt_upper-wt_test_set_wt_lower)*200,(wt_test_set_mutant1_upper-wt_test_set_mutant1_lower)*200, fc='none',ec='#8C0B6C',lw=3.5, linestyle='dashed')
ax_inset2.add_patch(rect)

# inset border
rect=plt.Rectangle((1,180.1),199,20, ec='k', fc='none', lw=1)
ax_inset2.add_patch(rect)

# inset background
rect=plt.Rectangle((1,180),199,20, fc='#216E02', alpha=0.1, lw=1)
ax_inset2.add_patch(rect)


##################################################################################################################
# Zoom effect
##################################################################################################################

# on original figure
rect=plt.Rectangle((180,0),20.5,200.5, fc='#002591', alpha=0.08, lw=1)
ax.add_patch(rect)
rect=plt.Rectangle((180,0),20.5,200.5, ec='#002591', fc='none', lw=1)
ax.add_patch(rect)
rect=plt.Rectangle((0,180),200.5,20.5, fc='#216E02', alpha=0.08, lw=1)
ax.add_patch(rect)
rect=plt.Rectangle((0,180),200.5,20.5, ec='#216E02', fc='none', lw=1)
ax.add_patch(rect)

# Mutant 1 inset connecting lines
l = Line2D([128,200],[15.5,-0.1], color='#002591',linestyle='dashed',lw=1.4)
ax.add_line(l)
l = Line2D([65.5,180],[15.5,-0.1], color='#002591',linestyle='dashed',lw=1.4)
ax.add_line(l)
l = Line2D([128,200],[76.5,200], color='#002591',linestyle='dashed',lw=1.4)
ax.add_line(l)
l = Line2D([65.5,180],[76.5,200], color='#002591',linestyle='dashed',lw=1.4)
ax.add_line(l)

# WT inset connecting lines
l = Line2D([128,200.5],[96,180], color='#216E02',linestyle='dashed',lw=1.6)
ax.add_line(l)
l = Line2D([128,200.5],[158,200], color='#216E02',linestyle='dashed',lw=1.6)
ax.add_line(l)
l = Line2D([67.1,0],[96,180], color='#216E02',linestyle='dashed',lw=1.6)
ax.add_line(l)
l = Line2D([67.1,0],[158,200], color='#216E02',linestyle='dashed',lw=1.6)
ax.add_line(l)

##################################################################################################################
# Saving figure
##################################################################################################################

if (save_figures):
    figure_ID = (time.strftime("%m%d_%H%M%S"))
    plt.savefig(figure_dir + 'Mutant1_vs_WT_test_set_figure_ID_' + str(figure_ID) + '.' + figure_format, bbox_inches='tight', format=figure_format)
```

---

### Clearing error and heatmap directories:¶

In [48]:

```
import shutil
for error_file in os.listdir(slurm_dir):
    file_path = os.path.join(slurm_dir, error_file)
    if os.path.isfile(file_path):
        os.unlink(file_path)
for heatmap_file in os.listdir(heatmap_output_dir):
    file_path = os.path.join(heatmap_output_dir, heatmap_file)
    if os.path.isfile(file_path):
        os.unlink(file_path)
```

### Collecting test set positions in the above clusters:¶

In [51]:

```
import subprocess
import os

for filename in os.listdir(mpileup_SNV_input_dir):
	if (filename.split("-")[0] in excluded_chromosomes):
		continue
	try:
		a = subprocess.call([ 'sbatch', '--mem',str(1000), '--exclude=jimgray84', '--output=' + slurm_dir + filename.split(".")[0] + '_slurm_out.txt', './test_set_collection_SNV.py' , filename, mpileup_SNV_input_dir, WT_test_set_output_dir, Mutant1_test_set_output_dir, sample_info, str(excluded_samples), str(wt_test_set_wt_lower), str(wt_test_set_wt_upper), str(wt_test_set_mutant1_lower), str(wt_test_set_mutant1_upper), str(mutant1_test_set_wt_lower), str(mutant1_test_set_wt_upper), str(mutant1_test_set_mutant1_lower), str(mutant1_test_set_mutant1_upper)],stderr=subprocess.STDOUT),
	except subprocess.CalledProcessError, e:
		print e.output,
```

### Cleaning error directory:¶

In [52]:

```
import shutil
for error_file in os.listdir(slurm_dir):
    file_path = os.path.join(slurm_dir, error_file)
    if os.path.isfile(file_path):
        os.unlink(file_path)
```

---


---

### Python codes for analysis:¶

In [53]:

```
%%writefile test_set_generation_SNV.py
#!/usr/bin/python

##################################################################################################################
# importing modules
##################################################################################################################

import sys
import re
import numpy as np

##################################################################################################################
# data from commandline
##################################################################################################################

filename = sys.argv[1]
input_dir = sys.argv[2]
output_dir = sys.argv[3]
sample_info_file = sys.argv[4]
excluded_samples = sys.argv[5][2:-2].split('\', \'')

##################################################################################################################
# loading sample info file
##################################################################################################################

samples=[]
genotypes=[]
treatments=[]
si_file = open(sample_info_file)
for nextline in iter(si_file.readline, b''):
	samples.append(nextline.strip('\n').split('\t')[0])
	genotypes.append(nextline.strip('\n').split('\t')[1])
	treatments.append(nextline.strip('\n').split('\t')[2])
si_file.close()

##################################################################################################################
# collecting included samples
##################################################################################################################

included_samples_bool = []
for i in xrange(len(samples)):
	included_samples_bool.append(not (samples[i] in excluded_samples))
included_samples_bool = np.array(included_samples_bool)

##################################################################################################################
# defining sample groups
##################################################################################################################

mutant1_bool = []
wt_bool = []
for sample in samples:
	mutant1_bool.append(genotypes[samples.index(sample)] == 'Mutant 1' and included_samples_bool[samples.index(sample)])
	wt_bool.append(genotypes[samples.index(sample)] == 'WT' and included_samples_bool[samples.index(sample)])
mutant1_bool = np.array(mutant1_bool)
wt_bool = np.array(wt_bool)

##################################################################################################################
# heatmap resolution
##################################################################################################################

resolution = 200
hist_matrix = np.zeros((resolution+1,resolution+1),dtype=np.int32)

##################################################################################################################
# processing positions in pileup files
##################################################################################################################

f = open(input_dir + filename)
for nextline in iter(f.readline, b''):
	line = nextline.strip('\n').upper().split(' ')
	if (line[3] == "N" or line[0]=="#"):
		continue
	covs=np.array(map(int,line[3::2]),dtype=np.int32)
	bases=line[4::2]
    
	# only consider positions where all samples are covered
	if (min(covs[mutant1_bool]) == 0 or min(covs[wt_bool]) == 0):
		continue
    
	ref_db = np.zeros(len(bases))
	for i in xrange(len(bases)):
		ref_db[i] = len(re.findall('[\.\,]',bases[i]))
    
	mutant1_mean_rnf = np.mean(ref_db[mutant1_bool]/covs[mutant1_bool])
	wt_mean_rnf = np.mean(ref_db[wt_bool]/covs[wt_bool])

	hist_matrix[int(resolution*mutant1_mean_rnf),int(resolution*wt_mean_rnf)]+=1
    
f.close()

##################################################################################################################
# saving heatmap to output file
##################################################################################################################

np.savetxt(output_dir + filename.split("/")[-1].split(".")[0] + '.hist_matrix',hist_matrix,fmt='%d')
```

```
Overwriting test_set_generation_SNV.py
```

In [54]:

```
%%writefile test_set_collection_SNV.py
#!/usr/bin/python

##################################################################################################################
# importing modules
##################################################################################################################

import sys
import re
import numpy as np

##################################################################################################################
# data from commandline
##################################################################################################################

filename = sys.argv[1]
input_dir = sys.argv[2]
WT_test_set_output_dir = sys.argv[3]
Mutant1_test_set_output_dir = sys.argv[4]
sample_info_file = sys.argv[5]
excluded_samples = sys.argv[6][2:-2].split('\', \'')
wt_test_set_wt_lower = np.float(sys.argv[7])
wt_test_set_wt_upper = np.float(sys.argv[8])
wt_test_set_mutant1_lower = np.float(sys.argv[9])
wt_test_set_mutant1_upper = np.float(sys.argv[10])
mutant1_test_set_wt_lower = np.float(sys.argv[11])
mutant1_test_set_wt_upper = np.float(sys.argv[12])
mutant1_test_set_mutant1_lower = np.float(sys.argv[13])
mutant1_test_set_mutant1_upper = np.float(sys.argv[14])

##################################################################################################################
# loading sample info file
##################################################################################################################

samples=[]
genotypes=[]
treatments=[]
si_file = open(sample_info_file)
for nextline in iter(si_file.readline, b''):
	samples.append(nextline.strip('\n').split('\t')[0])
	genotypes.append(nextline.strip('\n').split('\t')[1])
	treatments.append(nextline.strip('\n').split('\t')[2])
si_file.close()

##################################################################################################################
# collecting included samples
##################################################################################################################

included_samples_bool = []
for i in xrange(len(samples)):
	included_samples_bool.append(not (samples[i] in excluded_samples))
included_samples_bool = np.array(included_samples_bool)

##################################################################################################################
# defining sample groups
##################################################################################################################

mutant1_bool = []
wt_bool = []
for sample in samples:
	mutant1_bool.append(genotypes[samples.index(sample)] == 'Mutant 1' and included_samples_bool[samples.index(sample)])
	wt_bool.append(genotypes[samples.index(sample)] == 'WT' and included_samples_bool[samples.index(sample)])
mutant1_bool = np.array(mutant1_bool)
wt_bool = np.array(wt_bool)

##################################################################################################################
# opening output files
##################################################################################################################

f_out_mutant1 = open(Mutant1_test_set_output_dir + filename.split("/")[-1], 'a')
f_out_wt = open(WT_test_set_output_dir + filename.split("/")[-1], 'a')

##################################################################################################################
# processing pileup files
##################################################################################################################

f = open(input_dir + filename)
for nextline in iter(f.readline, b''):
	line = nextline.strip('\n').upper().split(' ')
	if (line[3] == "N" or line[0]=="#"):
		continue
	covs=np.array(map(int,line[3::2]),dtype=np.int32)
	bases=line[4::2]
    
	# skip positions where any of the included samples have zero coverage
	if (min(covs[included_samples_bool]) == 0):
		continue

	ref_db = np.zeros(len(bases))
	for i in xrange(len(bases)):
		ref_db[i] = len(re.findall('[\.\,]',bases[i]))  
    
	mutant1_mean_rnf = np.mean(ref_db[mutant1_bool]/covs[mutant1_bool])
	wt_mean_rnf = np.mean(ref_db[wt_bool]/covs[wt_bool])
    
	if (mutant1_mean_rnf >= mutant1_test_set_mutant1_lower and mutant1_mean_rnf <= mutant1_test_set_mutant1_upper and wt_mean_rnf >= mutant1_test_set_wt_lower and wt_mean_rnf <= mutant1_test_set_wt_upper):
		f_out_mutant1.write(nextline)
	if (mutant1_mean_rnf >= wt_test_set_mutant1_lower and mutant1_mean_rnf <= wt_test_set_mutant1_upper and wt_mean_rnf >= wt_test_set_wt_lower and wt_mean_rnf <= wt_test_set_wt_upper):
		f_out_wt.write(nextline)
    
f.close()

f_out_mutant1.close()
f_out_wt.close()
```

```
Overwriting test_set_collection_SNV.py
```
