# Supplementary material for: Fast and accurate mutation detection in whole genome sequences of multiple isogenic samples with IsoMut
Source: BMC Bioinformatics. 2017 Jan 31;18:73. doi: 10.1186/s12859-017-1492-4 (PMC5282906; doi:10.1186/s12859-017-1492-4)
Supplement: Additional file 5: — Verification and description of filtering parameters. A detailed verification of the chosen filtering parameters. (PDF 391 kb) [file 12859_2017_1492_MOESM5_ESM.pdf]

Additional file 5 – Verification of applied filtering parameters

Fast and accurate mutation detection in whole genome sequences of multiple isogenic samples with IsoMut

O. Pipek, D. Ribli, J. Molnár, Á. Póti, M. Krzystanek, A. Bodor, G. E. Tusnády, Z. Szallasi, I. Csabai, and D. Szüts

The basic intuition for SNV detection is to find positions in a sample where sequenced reads 'significantly differ' from the reference nucleotide. To measure this difference, we define the value of mutation frequency (*sample_mut_freq*), which is determined as the number of the most common non-reference reads in a given sample, divided by the sample coverage. Low non-zero values (< 0.1) of *sample_mut_freq* are more likely due to noise than an actual mutation, thus the acceptance threshold of *sample_mut_freq_min* was set as the first filtering parameter. Figure S5A shows the distribution of *sample_mut_freq* values for a Mutant 1 starting clone sample for both test set and non-test set positions. It appears that most test set positions (thus 'real mutations') have mutational frequencies around 0.5. On the other hand, non-test set positions (false positives) have peaks at both very low and very high frequencies, implying that a cut-off based on this variable is sensible to eliminate the noise-induced peak at *sample_mut_freq* ~ 0.

Despite being intuitive, the above approach alone leaves numerous false SNVs (non-test set positions) among the candidates. The three main sources of false somatic SNVs appearing throughout analysis are errors during both sequencing and alignment [S1], and also polymorphisms (SNPs) between the starting genotypes. The elimination of the effects of mostly random sequencing errors is addressed with the filtering of base quality to values not lower than 30 as described in Supplementary file 2.

Alignment errors and polymorphisms, however, are not expected to occur randomly, but rather to be present in multiple samples at the same genomic position. Problematic regions for alignment are usually ones where the reference genome is highly repetitive [S2], and SNPs appear before the separation of starting clones and their respective treatment afterward, resulting in shared mutations in differently treated samples. This presents the opportunity to filter out false SNVs based on the idea that in these positions, multiple samples should be heterozygous or at least 'noisy'. To control the maximal noise level that we deem acceptable in the currently non-selected ('other') samples, the *other_rnf_min* filtering parameter was

introduced. In practice, this results in discarding all SNV candidates in a given sample, where in the same genomic position at least one other sample has an *rnf* that falls below the *other_rnf_min* limit.

To demonstrate the validity of setting the *other_rnf_min* limit, we considered all non-test set positions where the mutational frequency is high enough in the Mutant 1 starting clone to indicate a possible mutation ($0.25 < sample\_mut\_freq \leq 1$). These candidates would evidently not be filtered out using only the *sample_mut_freq_min* limit, leaving a large set of false positives among the candidates. We generated a histogram based on the smallest value of *other_rnf* in the given position for all non-Mutant 1 samples. The results are shown on Figure S5B. Non-test set positions with high mutational frequency in the investigated sample tend to greatly differ from the reference genome in other samples as well. This implies that these mutations are not unique to only Mutant 1 samples but are also present in some other ones as well.

The peak at *other_rnf* ~ 0 consists of positions where the Mutant 1 starting clone is heterozygous, while some non-Mutant 1 samples (that is, WT samples) are homozygous non-reference. This feature characterizes positions where an LOH event occurred in the WT genotype. The smaller peak around *other_rnf* ~ 0.5 is caused by positions where both the Mutant 1 starting clone and some of the WT samples are heterozygous. Both these kinds of positions should be filtered out when looking for unique SNVs. By applying an *other_rnf_min* filtering parameter, as suggested, these SNV candidates are discarded on the basis of being present or noisy in multiple samples.

Furthermore, nucleotide frequencies are affected by the local sequence coverage, lower coverage resulting in more positions with higher sample_mut_freq values. On Figure S5C we plotted both test set and non-test set positions based on *sample_mut_freq* and sample coverage (*sample_cov*). Non-test set positions (that were not discarded by the *other_rnf_min* filter) usually fall in the $sample\_mut\_freq \geq 0.3$ range only when the coverage is low. We, therefore, considered filtering the positions based on minimum requirements for the coverage of the selected sample (*sample_cov_min* filter). An implicit coverage filter was also applied to other samples by ensuring that all investigated samples were covered with at least one read at every included position.

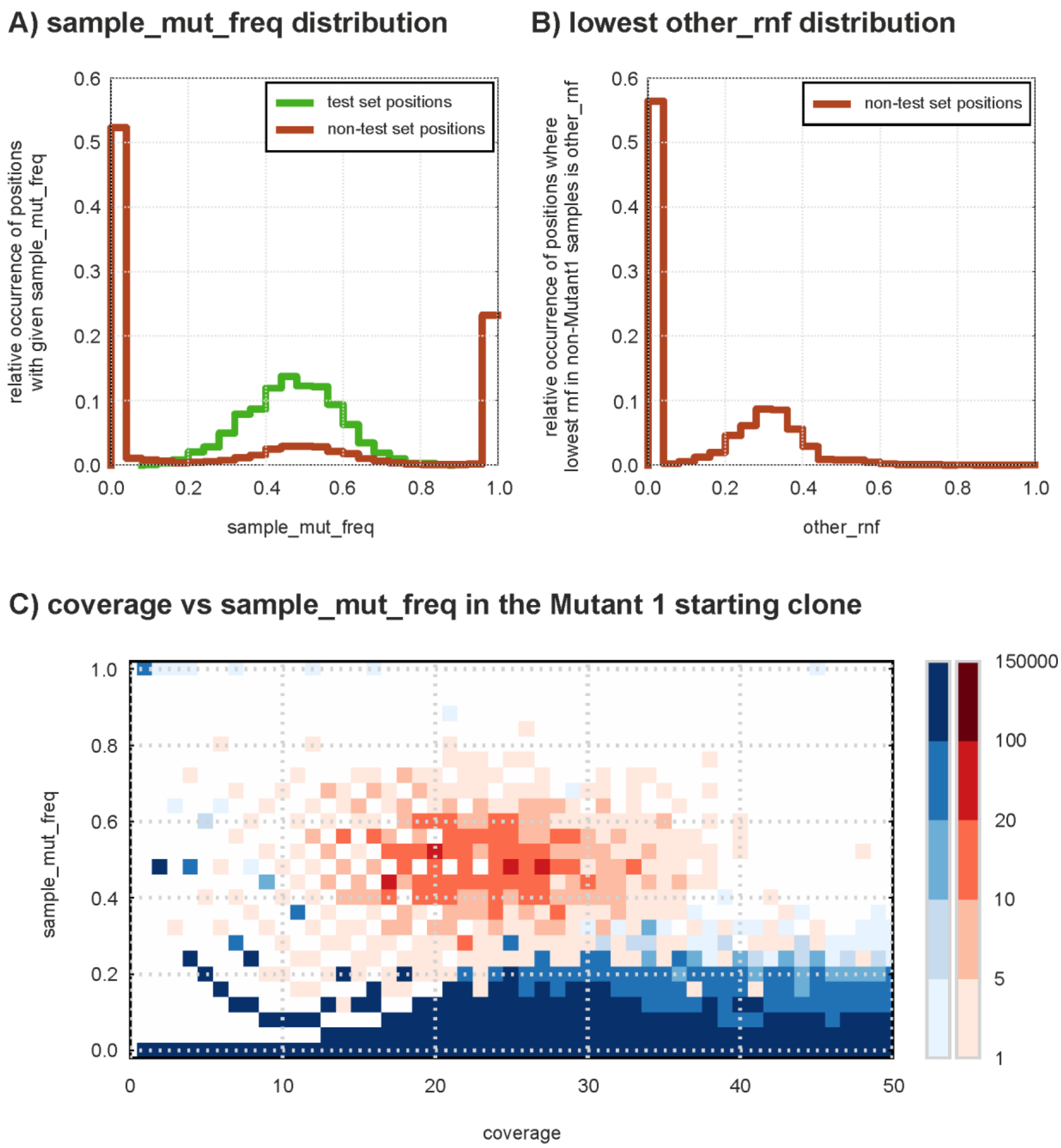

Figure S5. A) Distribution of sample_mut_freq values in test set and non-test set positions for a Mutant 1 starting clone. Test set positions have sample_mut_freq values around 0.5, while non-test set positions often have a non-zero but still very low mutation frequency (< 0.1). To filter out such 'noisy' positions from potential SNV candidates a threshold of *sample_mut_freq_min* was set. B) Distribution of the lowest other_rnf values of non-Mutant 1 samples, in non-test set positions where 0.25 < sample_mut_freq ≤ 1 in the Mutant 1 starting clone. C) Plotting sample_cov vs sample_mut_freq (in the Mutant 1 starting clone sample) for test set (red) and non-test set (blue) positions. According to previous considerations, positions where at least one non-Mutant 1 sample had rnf < 0.7 were categorized as possible genotype-specific mutations and thus discarded from this plot. This is a fairly weak filtering used only for demonstration purposes.

References

[S1] Hugo Y K Lam, Michael J Clark, Rui Chen, Rong Chen, Georges Natsoulis, Maeve O'Huallachain, Frederick E Dewey, Lukas Habegger, Euan A Ashley, Mark B Gerstein, Atul J Butte, Hanlee P Ji & Michael Snyder. Performance comparison of whole-genome sequencing platforms. Nature Biotechnology 30, 78–82 (2012) doi:10.1038/nbt.2065

[S2] Heng Li. Toward better understanding of artifacts in variant calling from high-coverage samples. Bioinformatics (2014) 30 (20): 2843-2851. doi:10.1093/bioinformatics/btu356
