# Supplementary material for: Fast and accurate mutation detection in whole genome sequences of multiple isogenic samples with IsoMut
Source: BMC Bioinformatics. 2017 Jan 31;18:73. doi: 10.1186/s12859-017-1492-4 (PMC5282906; doi:10.1186/s12859-017-1492-4)
Supplement: Additional file 6: — Example run and tuning of IsoMut. An example run of IsoMut on a reduced dataset for easy testing. (HTML 1353 kb) [file 12859_2017_1492_MOESM6_ESM.html]

IsoMut\_example\_run\_30\_samples


# Additional file 6 - IsoMut run on 30 samples¶

### Fast and accurate mutation detection in whole genome sequences of multiple isogenic samples with IsoMut¶

##### O. Pipek, D. Ribli, J. Molnár, Á. Póti, M. Krzystanek, A. Bodor, G. E. Tusnády, Z. Szallasi, I. Csabai, and D. Szüts¶

---

## Overview:¶

- The ipython notebook below shows how IsoMut was run on the samples described in the article. After slight modifications to input and output directories, it can be run on any data, whenever multiple isogenic samples are available.
- Please note that the following ipython notebook uses the Slurm Workload Manager (http://slurm.schedmd.com/) to run scripts in parallel, thus the syntax is adjusted for this special case. Whenever a different workload manager is preferred, the notebook should be adjusted accordingly. The other parts of the code can be used without significant modifications.
- This notebook also demonstrates the tuning of the mutation quality score (score), when control samples are available. In these samples no unique, treatment-induced mutations should be present, thus the score can be tuned by minimizing the number of detected mutations in these samples, while maintaining satisfyingly high numbers in samples that underwent strong mutagenic treatments. The tuning procedure can be individually carried out for SNVs, insertions and deletions, achieving optimized results for all types of mutations.
- The value of the score is related to the probability of correctly categorizing a candidate mutation as a true positive. More precisely, the probability p of incorrectly detecting a difference in the genotypes of the two noisiest samples in the given position can be calculated by Fisher’s exact test on the two samples. This gives the probability of being wrong to state that the reads found in the two noisiest samples are from different nucleotide distributions. The score is calculated as the negative logarithm of p. Thus a higher score value gives a more probable mutation.
- The notebook contains shell and python commands combined.

Note For a rapid testing of the IsoMut tool with data files included, see http://www.genomics.hu/tools/isomut/isomut\_howto.html

## Steps:¶

- 1, Download IsoMut from github
- 2, Compile it
- 3, Modify the example script (input/output directories, sample info) and save it
- 4, Submit job to queue using the preferred workload manager
- 5, Analyze results

---

## Preparations¶

#### Prepare the application¶

- download IsoMut from github
- compile it
- make output directory

In [1]:

```
%%bash 

########################################
# Download from git
git clone https://github.com/genomicshu/isomut.git
cd isomut

# compile
gcc -c -O3 isomut_lib.c fisher.c  -W -Wall
gcc -O3 -o isomut isomut.c isomut_lib.o  fisher.o -lm -W -Wall
```

In [2]:

```
import os
os.chdir('isomut/')
```

## Modifying example script and saving it¶

#### Please adjust:¶

- location of the reference genome (fasta file)
- input directory of bam files
- output directory to save results
- sample names and bam file names (example: If one has bam files of similar structure like ‘Sample1\_data.bam’, ‘Sample1’ would be the sample name and ‘\_data.bam’ the file name. File name is constant for all samples.)
- *Other parameters can also be modified, but it is not necessary for running the application.*

In [3]:

```
%%writefile pp_weak_sample_strong_noise.py
#!/usr/bin/env python
#################################################
# importing the wrapper
#################################################
#add path for isomput_parallel.py if its not here
import sys,os,subprocess
sys.path.append(os.getcwd())
#load the parallel wraooer function
from isomut_wrappers import run_isomut_with_pp

#################################################
# defining administrative parameters
#################################################
# using parameter dictionary
params=dict()
# minimum number of blocks to run
# usually there will be 10-20 more blocks
params['n_min_block']=100
# number of concurrent processes to run
params['n_conc_blocks']=8
# genome
params['ref_fasta']="/home/ribli/input/index/gallus/Gallus_gallus.Galgal4.74.dna.toplevel.fa"
# input dir output dir
params['input_dir']='/nagyvinyok/adat83/sotejedlik/orsi/bam_all_links_methodpaper/'
params['output_dir']='output/'
# the bam files used
samples=['S01','S02','S03','S04','S05','S06','S07','S08',
         'S09','S10','S11','S12','S13','S14','S15',
         'S16','S17','S18','S19','S20','S21','S22','S23',
         'S24','S25','S26','S27','S28','S29','S30']
params['bam_filenames']=[sample+'_RMdup_picard_realign.bam' for sample in samples ]

# limit chromosomes (for references with many scaffolds)
# comment/delete this line if you want to analyze all contigs in the ref genome
params['chromosomes']=map(str,range(1,29))+ ['32','W','Z']

#################################################
# defining mutation calling parameters
#    default values here ...
#################################################
params['min_sample_freq']=0.2
params['min_other_ref_freq']=0.93
params['cov_limit']=5
params['base_quality_limit']=30
params['min_gap_dist_snv']=0
params['min_gap_dist_indel']=20

#################################################
# running mutation calling algorithm
#################################################
run_isomut_with_pp(params)
```

```
Overwriting pp_weak_sample_strong_noise.py
```

## Submit job to queue using slurm workload manager¶

- Please adjust according to the syntax of the preferred workload manager.

In [5]:

```
%%bash
sbatch -c 24 --mem 15G -C jimgray83 pp_weak_sample_strong_noise.py
```

```
Submitted batch job 340322
```

---


---

## Analyzing results¶

#### Loading necessary python modules¶

In [1]:

```
import pandas as pd
import numpy as np
import subprocess

import matplotlib.pyplot as plt
%matplotlib inline
```

#### Collecting results into one file¶

In [8]:

```
%%bash

head -n1 output/all_SNVs.isomut    > all_output.csv
cat output/all_SNVs.isomut | grep -v sample >> all_output.csv
cat output/all_indels.isomut | grep -v sample >> all_output.csv
```

#### Loading data¶

In [11]:

```
output=pd.read_csv('all_output.csv',sep='\t',header=0)
```

### Plotting the number of mutations found in each sample for different score thresholds¶

In [20]:

```
##define sample groups
control_idx=[0,4,13]+ [15,26,29]
weak_idx=[5,6,7]+[19,20,21]
strong_idx=[1,2,3,8,9,10,11,12,14]+[16,17,18,22,23,24,25,27,28]

def plot_tuning_curve(output,ymax):
    #set cols
    cols=['lightgreen' for i in xrange(30)]
    for i in control_idx:
        cols[i]='dodgerblue' 
    for i in weak_idx:
        cols[i]='salmon' 

    fig,ax=plt.subplots()
    fig.set_size_inches(12,9)
    for i,col in zip(range(30),cols):
        score=output[(output['#sample']==i)].sort(['score'])['score']
        ax.plot(score,len(score)-np.arange(len(score)),c=col,lw=4,label='')
    ax.set_xlabel(r'score threshold',fontsize=16)
    ax.set_ylabel(r'Mutations found',fontsize=16)
    ax.set_ylim(1,ymax)
    ax.set_xlim(0,4)
    ax.grid()
    dump=ax.legend(loc='upper right',fancybox='true',fontsize=16)


    fig,ax=plt.subplots()
    fig.set_size_inches(12,9)
    for i,col in zip(range(30),cols):
        score=output[(output['#sample']==i)].sort(['score'])['score']
        ax.plot(score,len(score)-np.arange(len(score)),c=col,lw=4,label='')

    ax.set_xlabel(r'score threshold',fontsize=16)
    ax.set_ylabel(r'Mutations found',fontsize=16)
    ax.set_ylim(1,ymax)
    ax.set_xlim(0,4)
    ax.set_yscale('log')
    ax.grid()
    #dump=ax.legend(loc='upper right',fancybox='true',fontsize=16)
```

#### Plotting SNV counts¶

- It can be seen that by setting the score threshold to ~ 2.5 there are fewer than 10 mutations in the control samples, while the treated samples still have thousands of mutations. Thus with this optimization step, control and treated samples are separated by a factor of 100 in the number of their mutations.

In [21]:

```
plot_tuning_curve(output[output['type']=='SNV'],4e3)
```

#### Plotting insertion counts¶

- The appropriate score threshold for insertions is around 1.5. This way there are almost no mutations in control samples, while treated samples have around a hundred.

In [22]:

```
plot_tuning_curve(output[output['type']=='INS'],100)
```

#### Plotting deletion counts¶

- The appropriate score threshold for deletions is also around 1.5. This way there are almost no mutations in control samples, while treated samples have around a hundred.

In [23]:

```
plot_tuning_curve(output[output['type']=='DEL'],300)
```

## Plotting a quasi-ROC curve for score optimization¶

- False positive rate: the ratio of mutations found in control samples to the length of the genome
- For a fast and convenient optimization process, true positives were estimated as the mutations found in all treated samples. Some of these may in fact be false positives, but as the mutation rate in treated samples are higher by a factor of 100-1000 than in control samples, this estimation still gives reasonable results.
- The ROC curves below show how the change in the selected score threshold affects the number of false and true positive results. For our purposes, very low false positive rates were necessary, thus we chose fairly strict filtering values to achieve this.

In [17]:

```
def plot_roc(output,score0,score_sel):

    fig,ax=plt.subplots()
    fig.set_size_inches(12,9)
    fp, tp = [0  for i in xrange(100) ],[0  for i in xrange(100) ]
    for score_lim,j in zip(np.linspace(score0,10,100),range(100)):
        muts=[]
        for i in xrange(30):
            filt_idx =  output['#sample'] ==  i
            filt_idx = filt_idx & ((output['score']>score_lim))
            muts.append(len(output[filt_idx]))
        muts=np.array(muts)
        fp[j] ,tp[j]=1e-3*np.mean(muts[control_idx]),1e-3*np.mean(muts[weak_idx+strong_idx])
    ax.step(fp,tp,c='magenta',lw=4,label='quasy ROC, scannning the tuning parameter')
     
    muts=[]
    for i in xrange(30):
        filt_idx =  output['#sample'] ==  i
        filt_idx = filt_idx & ((output['score']>score_sel))
        muts.append(len(output[filt_idx]))
    muts=np.array(muts)

    ax.plot(1e-3*np.mean(muts[control_idx]),1e-3*np.mean(muts[weak_idx+strong_idx]),
            'o',mec='dodgerblue',mfc='dodgerblue',ms=15,mew=3,label='selected parameter')
    ax.legend(fancybox=True,loc='lower right',fontsize=16)

    #ax.set_xlim(0,30)
    ax.set_ylim(ymin=0)
    ax.set_xlim(xmin=-0.0001)
    ax.set_xlabel('false positive rate 1/Mbp ',fontsize=16)
    dump=ax.set_ylabel('mutation rate  1/Mbp ',fontsize=16)
```

### SNVs¶

In [18]:

```
plot_roc(output[output['type']=='SNV'],score0=1.5,score_sel=2.4)
```

### Insertions¶

- Please note, that the selected score threshold for insertions/deletions is very different than the score threshold chosen for SNVs, because these types of mutations appear both with different mutation and different noise frequencies

In [19]:

```
plot_roc(output[output['type']=='INS'],score0=1,score_sel=1.5)
```

### Deletions¶

In [20]:

```
plot_roc(output[output['type']=='DEL'],score0=1,score_sel=1.8)
```

---

## Plotting the final results with optimized score thresholds¶

---

In [26]:

```
#define plotting func
def plot_mutres(table):
    #group mutations per samples
    sample_counts=table.groupby(['#sample'],as_index=False).count()[['#sample','mut']]
    sample_counts.columns=['sample','count']

    #add zeroes if a sample is missing
    N_samples=30
    for i in xrange(N_samples):
        if (not i in set(sample_counts['sample'])):
            sample_counts=pd.concat([sample_counts,pd.DataFrame({'sample':[i], 'count' : [0]})])
            sample_counts=sample_counts.sort('sample').reset_index()[['sample','count']]

    #plot
    fig,ax=plt.subplots()
    fig.set_size_inches(12,9)

    #starting clones and controls
    ax.bar(control_idx,[sample_counts.loc[i,'count'] for i in control_idx],
           facecolor='dodgerblue',edgecolor='none',label='starting clone and controls')
    #weak treatment
    ax.bar(weak_idx,[sample_counts.loc[i,'count'] for i in weak_idx],
           facecolor='salmon',edgecolor='none',label='weak mutagenic treatment')
    #strong treatment
    ax.bar(strong_idx,[sample_counts.loc[i,'count'] for i in strong_idx],
           facecolor='lightgreen',edgecolor='none',label='strong mutagenic treatment')

    #samples labels
    names=['S01','S02','S03','S04','S05','S06','S07',
         'S08','S09','S10','S11','S12','S13','S14','S15',
         'S16','S17','S18','S19','S20','S21','S22','S23',
         'S24','S25','S26','S27','S28','S29','S30']

    ax.set_xticks(0.4+np.arange(len(names)))
    ax.set_xticklabels(names,rotation='vertical',fontsize=14)
    #axis, and legend
    ax.set_xlabel(r'samples',fontsize=18)
    ax.set_ylabel(r'Mutations detected',fontsize=18)
    dump=ax.legend(loc='best',fancybox='true',fontsize=16)
    
    #print the table
    sample_counts['code']=names
    return sample_counts[['code','count']]
```

### SNVs¶

In [27]:

```
plot_mutres(output[(output['type']=='SNV' ) & (output['score']> 2.4)] )
```

Out[27]:

|  | code | count |
| --- | --- | --- |
| 0 | S01 | 0 |
| 1 | S02 | 668 |
| 2 | S03 | 100 |
| 3 | S04 | 128 |
| 4 | S12 | 3 |
| 5 | S05 | 60 |
| 6 | S06 | 115 |
| 7 | S07 | 36 |
| 8 | S08 | 1247 |
| 9 | S09 | 2816 |
| 10 | S10 | 2136 |
| 11 | S11 | 1057 |
| 12 | S13 | 1101 |
| 13 | S15 | 1 |
| 14 | S14 | 96 |
| 15 | S16 | 1 |
| 16 | S17 | 1448 |
| 17 | S18 | 418 |
| 18 | S19 | 519 |
| 19 | S20 | 522 |
| 20 | S21 | 555 |
| 21 | S22 | 317 |
| 22 | S23 | 2460 |
| 23 | S24 | 2309 |
| 24 | S25 | 2055 |
| 25 | S26 | 1643 |
| 26 | S27 | 3 |
| 27 | S28 | 2331 |
| 28 | S29 | 1550 |
| 29 | S30 | 5 |

### Insertions¶

In [28]:

```
plot_mutres(output[(output['type']=='INS' ) & (output['score']> 1.5)] )
```

Out[28]:

|  | code | count |
| --- | --- | --- |
| 0 | S01 | 0 |
| 1 | S02 | 22 |
| 2 | S03 | 3 |
| 3 | S04 | 4 |
| 4 | S12 | 0 |
| 5 | S05 | 5 |
| 6 | S06 | 1 |
| 7 | S07 | 6 |
| 8 | S08 | 1 |
| 9 | S09 | 2 |
| 10 | S10 | 2 |
| 11 | S11 | 37 |
| 12 | S13 | 50 |
| 13 | S15 | 1 |
| 14 | S14 | 4 |
| 15 | S16 | 0 |
| 16 | S17 | 42 |
| 17 | S18 | 1 |
| 18 | S19 | 6 |
| 19 | S20 | 8 |
| 20 | S21 | 9 |
| 21 | S22 | 9 |
| 22 | S23 | 9 |
| 23 | S24 | 14 |
| 24 | S25 | 5 |
| 25 | S26 | 30 |
| 26 | S27 | 2 |
| 27 | S28 | 79 |
| 28 | S29 | 49 |
| 29 | S30 | 0 |

### Deletions¶

In [29]:

```
plot_mutres(output[(output['type']=='DEL' ) & (output['score']>1.8)] )
```

Out[29]:

|  | code | count |
| --- | --- | --- |
| 0 | S01 | 0 |
| 1 | S02 | 63 |
| 2 | S03 | 4 |
| 3 | S04 | 6 |
| 4 | S12 | 1 |
| 5 | S05 | 0 |
| 6 | S06 | 3 |
| 7 | S07 | 1 |
| 8 | S08 | 6 |
| 9 | S09 | 12 |
| 10 | S10 | 10 |
| 11 | S11 | 52 |
| 12 | S13 | 82 |
| 13 | S15 | 0 |
| 14 | S14 | 2 |
| 15 | S16 | 0 |
| 16 | S17 | 64 |
| 17 | S18 | 11 |
| 18 | S19 | 15 |
| 19 | S20 | 17 |
| 20 | S21 | 8 |
| 21 | S22 | 10 |
| 22 | S23 | 26 |
| 23 | S24 | 20 |
| 24 | S25 | 31 |
| 25 | S26 | 61 |
| 26 | S27 | 1 |
| 27 | S28 | 138 |
| 28 | S29 | 81 |
| 29 | S30 | 0 |

---

#### Note: It finished in 7 hours¶

---
