# Supplementary material for: Fast and accurate mutation detection in whole genome sequences of multiple isogenic samples with IsoMut
Source: BMC Bioinformatics. 2017 Jan 31;18:73. doi: 10.1186/s12859-017-1492-4 (PMC5282906; doi:10.1186/s12859-017-1492-4)
Supplement: Additional file 7: — Table of tested parameter settings. List of tested parameter settings with the resulting TPR and FPR values. (PDF 307 kb) [file 12859_2017_1492_MOESM7_ESM.pdf]

Additional file 7 - Table of tested parameter settings

Fast and accurate mutation detection in whole genome sequences of multiple isogenic samples with IsoMut

O. Pipek, D. Ribli, J. Molnár, Á. Póti, M. Krzystanek, A. Bodor, G. E. Tusnády, Z. Szallasi, I. Csabai, and D. Szüts

sample_mut_freq_min	other_rnf_min	sample_cov_min	FPR	TPR
0.28	0.9	1.0	2.28342592087e-06	0.970179133905
0.28	0.9	5.0	2.29486759408e-07	0.966138514563
0.28	0.9	7.0	1.52917854682e-07	0.961501949918
0.28	0.9	10.0	5.1045999947e-08	0.9485671327
0.28	0.9	12.0	4.15848951617e-08	0.934006397099
0.28	0.9	15.0	2.92634520868e-08	0.875198429145
0.28	0.92	1.0	2.15867139455e-06	0.963458446486
0.28	0.92	5.0	1.9340254292e-07	0.959851854922
0.28	0.92	7.0	1.2607471443e-07	0.955215290277
0.28	0.92	10.0	3.91646059706e-08	0.942497486948
0.28	0.92	12.0	3.05836042029e-08	0.928153765236
0.28	0.92	15.0	2.33227547337e-08	0.870375770362
0.28	0.93	1.0	2.10718539919e-06	0.956004072855
0.28	0.93	5.0	1.77120644164e-07	0.95239748129
0.28	0.93	7.0	1.15073429141e-07	0.947760916646
0.28	0.93	10.0	3.32239086175e-08	0.935043113316
0.28	0.93	12.0	2.57430352167e-08	0.920699391605
0.28	0.93	15.0	2.04624178407e-08	0.863572438397
0.28	0.94	1.0	2.06780081804e-06	0.943806313418
0.28	0.94	5.0	1.66339389389e-07	0.940199721854
0.28	0.94	7.0	1.07372532517e-07	0.935563157209
0.28	0.94	10.0	3.0803627061e-08	0.92284535388
0.28	0.94	12.0	2.39828309638e-08	0.908908111764
0.28	0.94	15.0	1.98023412458e-08	0.853517269668
0.28	0.95	1.0	2.02885628543e-06	0.917240655657
0.28	0.95	5.0	1.55778155689e-07	0.913634064092
0.28	0.95	7.0	9.87915286024e-08	0.908997499447
0.28	0.95	10.0	2.64031089556e-08	0.896279696118
0.28	0.95	12.0	2.04624149848e-08	0.882748933598
0.28	0.95	15.0	1.7162028102e-08	0.829662599733
0.3	0.9	1.0	2.18353428719e-06	0.962263184313
0.3	0.9	5.0	1.29595125729e-07	0.958222564971
0.3	0.9	7.0	5.30262210024e-08	0.953586000326
0.3	0.9	10.0	4.22449621363e-08	0.941085210886
0.3	0.9	12.0	3.2783857351e-08	0.926524475285
0.3	0.9	15.0	2.39828256168e-08	0.869180508189
0.3	0.92	1.0	2.07440158623e-06	0.955759510784
0.3	0.92	5.0	1.09132734602e-07	0.952152919219
0.3	0.92	7.0	4.18049061118e-08	0.947516354575
0.3	0.92	10.0	3.25638266757e-08	0.935232579023
0.3	0.92	12.0	2.39828249081e-08	0.920888857311
0.3	0.92	15.0	1.89222321725e-08	0.864574863295
0.3	0.93	1.0	2.02863625719e-06	0.948305137152

0.3	0.93	5.0	9.85715021587e-08	0.944698545588
0.3	0.93	7.0	3.65242871359e-08	0.940061980943
0.3	0.93	10.0	2.83833360667e-08	0.927778205391
0.3	0.93	12.0	2.09024626658e-08	0.91343448368
0.3	0.93	15.0	1.69419991882e-08	0.85777153133
0.3	0.94	1.0	1.99409223933e-06	0.936107377716
0.3	0.94	5.0	9.26308106789e-08	0.932500786151
0.3	0.94	7.0	3.36639538075e-08	0.927864221506
0.3	0.94	10.0	2.59630545102e-08	0.915580445955
0.3	0.94	12.0	1.91422584129e-08	0.901643203839
0.3	0.94	15.0	1.62819225934e-08	0.847716362601
0.3	0.95	1.0	1.95976824609e-06	0.909731185661
0.3	0.95	5.0	8.66901163432e-08	0.906124594096
0.3	0.95	7.0	2.97034892563e-08	0.901488029451
0.3	0.95	10.0	2.26626665539e-08	0.8892042539
0.3	0.95	12.0	1.6721972583e-08	0.875673491379
0.3	0.95	15.0	1.45217140671e-08	0.824051158372
0.31	0.9	1.0	2.17319307868e-06	0.954753714317
0.31	0.9	5.0	1.19253917217e-07	0.950713094975
0.31	0.9	7.0	4.26850124907e-08	0.94607653033
0.31	0.9	10.0	3.19037536246e-08	0.93357574089
0.31	0.9	12.0	2.92634431543e-08	0.920072526552
0.31	0.9	15.0	2.22226179817e-08	0.864871150162
0.31	0.92	1.0	2.06560055715e-06	0.948250040788
0.31	0.92	5.0	1.0033170552e-07	0.944643449223
0.31	0.92	7.0	3.30038770302e-08	0.940006884579
0.31	0.92	10.0	2.37627975941e-08	0.927723109027
0.31	0.92	12.0	2.13425135466e-08	0.914436908578
0.31	0.92	15.0	1.7382050069e-08	0.860265505268
0.31	0.93	1.0	2.02093535736e-06	0.940795667156
0.31	0.93	5.0	9.0870602335e-08	0.937189075592
0.31	0.93	7.0	2.88233873123e-08	0.932552510947
0.31	0.93	10.0	2.0682436243e-08	0.920268735395
0.31	0.93	12.0	1.84821777271e-08	0.906982534946
0.31	0.93	15.0	1.54018170847e-08	0.853462173303
0.31	0.94	1.0	1.98727144234e-06	0.928814921609
0.31	0.94	5.0	8.58100136904e-08	0.925208330044
0.31	0.94	7.0	2.6843156819e-08	0.920571765399
0.31	0.94	10.0	1.91422575217e-08	0.908287989848
0.31	0.94	12.0	1.69419990058e-08	0.895408268994
0.31	0.94	15.0	1.47417404899e-08	0.843624018462
0.31	0.95	1.0	1.95426760389e-06	0.902438729553
0.31	0.95	5.0	8.11894741444e-08	0.898832137989
0.31	0.95	7.0	2.42028470575e-08	0.894195573344
0.31	0.95	10.0	1.7162024355e-08	0.881911797793
0.31	0.95	12.0	1.51817913707e-08	0.869438556534
0.31	0.95	15.0	1.3421583918e-08	0.819958814234
0.32	0.9	1.0	2.16989268876e-06	0.945590777757
0.32	0.9	5.0	1.15953527296e-07	0.941550158414
0.32	0.9	7.0	3.93846225697e-08	0.93691359377

0.32	0.9	10.0	2.86033637036e-08	0.924412804329
0.32	0.9	12.0	2.59630532333e-08	0.910909589991
0.32	0.9	15.0	1.89222280606e-08	0.855708213602
0.32	0.92	1.0	2.0636203222e-06	0.939087104227
0.32	0.92	5.0	9.83514705714e-08	0.935480512663
0.32	0.92	7.0	3.10236420812e-08	0.930843948018
0.32	0.92	10.0	2.17825626451e-08	0.918560172466
0.32	0.92	12.0	1.93622785975e-08	0.905273972017
0.32	0.92	15.0	1.540181512e-08	0.851102568708
0.32	0.93	1.0	2.01939517437e-06	0.931849744484
0.32	0.93	5.0	8.93304193404e-08	0.92824315292
0.32	0.93	7.0	2.72832043176e-08	0.923606588275
0.32	0.93	10.0	1.91422532483e-08	0.911322812724
0.32	0.93	12.0	1.69419947324e-08	0.898036612274
0.32	0.93	15.0	1.386163409e-08	0.844516250632
0.32	0.94	1.0	1.98573125934e-06	0.919868998937
0.32	0.94	5.0	8.42698306958e-08	0.916262407372
0.32	0.94	7.0	2.53029738244e-08	0.911625842728
0.32	0.94	10.0	1.76020745271e-08	0.899342067176
0.32	0.94	12.0	1.54018160111e-08	0.886462346322
0.32	0.94	15.0	1.32015574952e-08	0.834678095791
0.32	0.95	1.0	1.95272742089e-06	0.893709820771
0.32	0.95	5.0	7.96492911497e-08	0.890103229206
0.32	0.95	7.0	2.26626640628e-08	0.885466664561
0.32	0.95	10.0	1.56218413603e-08	0.87318288901
0.32	0.95	12.0	1.3641608376e-08	0.860709647752
0.32	0.95	15.0	1.18814009233e-08	0.811229905451
0.33	0.9	1.0	2.1687925595e-06	0.942907338112
0.33	0.9	5.0	1.14853398038e-07	0.93886671877
0.33	0.9	7.0	3.82844933117e-08	0.934230154125
0.33	0.9	10.0	2.75032344456e-08	0.921729364685
0.33	0.9	12.0	2.48629239753e-08	0.908226150346
0.33	0.9	15.0	1.78220988027e-08	0.853024773957
0.33	0.92	1.0	2.06274021847e-06	0.936403664583
0.33	0.92	5.0	9.7471366845e-08	0.932797073018
0.33	0.92	7.0	3.01435383548e-08	0.928160508373
0.33	0.92	10.0	2.09024589187e-08	0.915876732822
0.33	0.92	12.0	1.84821748712e-08	0.902590532372
0.33	0.92	15.0	1.45217113936e-08	0.848419129063
0.33	0.93	1.0	2.01851507064e-06	0.92916630484
0.33	0.93	5.0	8.8450315614e-08	0.925559713275
0.33	0.93	7.0	2.64031005913e-08	0.920923148631
0.33	0.93	10.0	1.8262149522e-08	0.908639373079
0.33	0.93	12.0	1.60618910061e-08	0.89535317263
0.33	0.93	15.0	1.29815303637e-08	0.841832810987
0.33	0.94	1.0	1.98485115562e-06	0.917592038888
0.33	0.94	5.0	8.33897269694e-08	0.913985447323
0.33	0.94	7.0	2.4422870098e-08	0.909348882679
0.33	0.94	10.0	1.67219708007e-08	0.897065107127
0.33	0.94	12.0	1.45217122848e-08	0.884185386273

0.33	0.94	15.0	1.23214537688e-08	0.832401135742
0.33	0.95	1.0	1.95184731717e-06	0.891432860722
0.33	0.95	5.0	7.87691874233e-08	0.887826269157
0.33	0.95	7.0	2.17825603364e-08	0.883189704512
0.33	0.95	10.0	1.4741737634e-08	0.870905928961
0.33	0.95	12.0	1.27615046496e-08	0.858432687703
0.33	0.95	15.0	1.10012971969e-08	0.808952945402
0.34	0.9	1.0	6.98141326226e-07	0.923579040093
0.34	0.9	5.0	5.52264596108e-08	0.920351379942
0.34	0.9	7.0	2.81633112229e-08	0.916555322671
0.34	0.9	10.0	2.28826927733e-08	0.905329068381
0.34	0.9	12.0	2.0242382303e-08	0.891825854043
0.34	0.9	15.0	1.47417365604e-08	0.838550054471
0.34	0.92	1.0	6.60956996823e-07	0.917075366564
0.34	0.92	5.0	4.64254300028e-08	0.91428173419
0.34	0.92	7.0	2.28826919031e-08	0.910485676919
0.34	0.92	10.0	1.87022018203e-08	0.899476436518
0.34	0.92	12.0	1.62819177727e-08	0.886190236069
0.34	0.92	15.0	1.32015564216e-08	0.833944409577
0.34	0.93	1.0	6.45335177861e-07	0.909838006821
0.34	0.93	5.0	4.20249163532e-08	0.907044374447
0.34	0.93	7.0	2.02423825063e-08	0.903248317176
0.34	0.93	10.0	1.62819179552e-08	0.892239076776
0.34	0.93	12.0	1.40816594392e-08	0.878952876326
0.34	0.93	15.0	1.16613753917e-08	0.827358091501
0.34	0.94	1.0	6.35874078585e-07	0.899104248242
0.34	0.94	5.0	3.93846092651e-08	0.896310615869
0.34	0.94	7.0	1.8702203785e-08	0.892514558598
0.34	0.94	10.0	1.47417392339e-08	0.881505318197
0.34	0.94	12.0	1.25414807179e-08	0.868625597343
0.34	0.94	15.0	1.10012987968e-08	0.818549909741
0.34	0.95	1.0	6.24872798622e-07	0.873813125632
0.34	0.95	5.0	3.71843505667e-08	0.871019493258
0.34	0.95	7.0	1.67219706183e-08	0.867223435987
0.34	0.95	10.0	1.29815315987e-08	0.856214195587
0.34	0.95	12.0	1.10012986144e-08	0.843740954328
0.34	0.95	15.0	9.68114222495e-09	0.795969774957
0.35	0.9	1.0	6.97701274272e-07	0.919242133884
0.35	0.9	5.0	5.47864076564e-08	0.916014473733
0.35	0.9	7.0	2.77232592685e-08	0.912218416461
0.35	0.9	10.0	2.24426408189e-08	0.900992162172
0.35	0.9	12.0	1.98023303486e-08	0.887488947834
0.35	0.9	15.0	1.4301684606e-08	0.834213148262
0.35	0.92	1.0	6.60516944869e-07	0.912738460354
0.35	0.92	5.0	4.59853780485e-08	0.909944827981
0.35	0.92	7.0	2.24426399487e-08	0.90614877071
0.35	0.92	10.0	1.82621498659e-08	0.895139530309
0.35	0.92	12.0	1.58418658184e-08	0.88185332986
0.35	0.92	15.0	1.27615044672e-08	0.829607503368
0.35	0.93	1.0	6.44895125907e-07	0.905501100612

0.35	0.93	5.0	4.15848643988e-08	0.902707468238
0.35	0.93	7.0	1.98023305519e-08	0.898911410967
0.35	0.93	10.0	1.58418660008e-08	0.887902170567
0.35	0.93	12.0	1.36416074848e-08	0.874615970117
0.35	0.93	15.0	1.12213234373e-08	0.823021185292
0.35	0.94	1.0	6.3543402663e-07	0.894767342033
0.35	0.94	5.0	3.89445573107e-08	0.89197370966
0.35	0.94	7.0	1.82621518306e-08	0.888177652388
0.35	0.94	10.0	1.43016872795e-08	0.877168411988
0.35	0.94	12.0	1.21014287636e-08	0.864288691134
0.35	0.94	15.0	1.05612468425e-08	0.814213003531
0.35	0.95	1.0	6.24432746667e-07	0.869476219422
0.35	0.95	5.0	3.67442986124e-08	0.866682587049
0.35	0.95	7.0	1.62819186639e-08	0.862886529778
0.35	0.95	10.0	1.25414796444e-08	0.851877289377
0.35	0.95	12.0	1.056124666e-08	0.839404048119
0.35	0.95	15.0	9.24109027057e-09	0.791632868748
