# Supplementary material for: Fast and accurate mutation detection in whole genome sequences of multiple isogenic samples with IsoMut
Source: BMC Bioinformatics. 2017 Jan 31;18:73. doi: 10.1186/s12859-017-1492-4 (PMC5282906; doi:10.1186/s12859-017-1492-4)
Supplement: Additional file 8: — Running VarScan 2 on our dataset. Computational details and results of running VarScan 2 on the described dataset. (HTML 408 kb) [file 12859_2017_1492_MOESM8_ESM.html]

VarScan\_best\_practice


# Additional file 8 - Running VarScan on identical control samples¶

### Fast and accurate mutation detection in whole genome sequences of multiple isogenic samples with IsoMut¶

##### O. Pipek, D. Ribli, J. Molnár, Á. Póti, M. Krzystanek, A. Bodor, G. E. Tusnády, Z. Szallasi, I. Csabai, and D. Szüts¶

---

### Assessing the specificity (false positive rate) of VarScan¶

VarScan expects a ‘normal’ and a ‘tumor’ sample pair as its input and detects somatic mutations in the tumor sample that are not present in the normal one using Fisher’s exact test. As in our dataset, two sample pairs with identical DNA sequences were available, we used one sample from a pair as the ‘tumor’ and the other one as the ‘normal’ sample (and vice versa). This way, all detected mutations are false positives as these pairs were created by sequencing the same DNA preparation twice.

VarScan 2 was run based on the best practices described here :

- http://www.ncbi.nlm.nih.gov/pmc/articles/PMC4278659/?tool=pmcentrez

In a later step, results were further filtered by tuning the somatic-p-value parameter.

### The pipeline (according to the article above)¶

#### Perform Somatic Mutation Calling¶

**1.** Run SAMtools mpileup on the BAM files for normal and tumor samples:

```
    samtools mpileup –B –q 1 –f reference.fasta normal.bam tumor.bam >normal-tumor.mpileup
```

**2.** Run VarScan in somatic mode, providing the mpileup file (normal-tumor.mpileup) and a basename for output files (output.basename):

```
   java –jar VarScan.jar somatic normal-tumor.mpileup
   output.basename –min-coverage 10 –min-var-freq 0.08 –somatic-
   p-value 0.05
```

The above recommended values of VarScan parameters were used throughout the run below. The above command will generate two output files, one for SNVs (output.basename.snp) and one for indels (output.basename.indel).

**3.** Run the processSomatic subcommand to divide the output into separate files based on somatic status and confidence:

```
   java –jar VarScan.jar processSomatic output.basename.snp
   java –jar VarScan.jar processSomatic output.basename.indel
```

This command will generate six files per input file. For SNVs, the output files will be:

```
- output.basename.snp.Somatic – all somatic mutations 
- output.basename.snp.Somatic.hc – high-confidence somatic mutations 
- output.basename.snp.LOH – all LOH events 
- output.basename.snp.LOH.hc – high-confidence LOH events 
- output.basename.snp.Germline – all germline variants 
- output.basename.snp.Germline.hc – high-confidence germline variants
```

The subset of high-confidence variants is determined using a few empirically-derived criteria. For example, high-confidence somatic mutations have tumor VAF>15%, normal VAF<5%, and a somatic p-value of <0.03. These are user-adjustable.

**4.** Run an additional filter on the somatic mutations

```
   java –jar VarScan.jar somaticFilter
   output.basename.snp.Somatic.hc –indel-file
   output.basename.indel –output-file
   output.basename.snp.Somatic.hc.filter
```

The above command identifies and removes somatic mutations that are likely false positives due to alignment problems near indels. After this step, candidate somatic mutations should also be filtered to remove other artifacts, as described in Support Protocol 1.

#### Support protocol¶

Run the False Positive Filter:

**+1.** Obtain metrics for the list of variants:

```
              bam-readcount –q 1 –b 20 –f reference.fasta –l
              varScan.variants BAM_FILE >varScan.variants.readcounts
```

**+2.** Run the FPfilter accessory script:

```
            perl fpfilter.pl varScan.variants varScan.variants.readcounts
              –output-basename varScan.variants.filter
```

The above command would create two output files. Variants passing the filter are found in varScan.variants.filter.pass while variants that fail are printed to varScan.variants.filter.fail along with the reason for the failure. Filtering parameters in the fpfilter.pl script are set to recommended values for Illumina paired-end (2×100 bp) reads, but can be modified by the user in the script if desired.

---

### Preparations¶

In [1]:

```
#load modules
import os
import subprocess
import time

#go to working directory
work_dir='/nagyvinyok/adat84/sotejedlik/ribli/dt40/method/varscan_best_practice'
subprocess.call(['mkdir',work_dir])
os.chdir(work_dir)

#gallus reference
galref="/home/ribli/input/index/gallus/complete/Gallus_gallus.Galgal4.74.dna.toplevel.fa"
input_dir='/nagyvinyok/adat84/sotejedlik/ribli/dt40/ident_bams/'
output_dir=work_dir
```

---

### Running samtools mpileup and VarScan in somatic mode (steps 1 and 2)¶

In [2]:

```
def run_samt_mp_varscan_som(tum_sample,norm_sample,input_dir,output_dir,ref_genome):
    #input files
    norm_bam=input_dir+norm_sample+'.bam'
    tum_bam=input_dir+tum_sample+'.bam'

    #create pileup commands
    cmd_mpileup=' <(samtools mpileup -B -q 1 -f '+ ref_genome +  ' ' + norm_bam+')'
    cmd_mpileup+=' <(samtools mpileup -B -q 1 -f '+ ref_genome +  ' '+ tum_bam +')'

    #varscan params
    pval=' 0.9 '
    
    #output file
    output=output_dir+'/'+tum_sample+'_'+norm_sample+'.vsc'

    #varscan command
    cmd='time java -jar VarScan.v2.3.7.jar somatic '+ cmd_mpileup + ' '+ output
    cmd+=' --min-coverage 10 --min-var-freq 0.08 --somatic-p-value 0.05 '
    print cmd,'\n'

    #write scriptfile for sbatch
    script_fn=tum_sample+'_'+norm_sample+'.sh'
    with open(script_fn,'w') as f:
        f.write('#!/bin/bash\n'+cmd+'\n')

    #submit script to sbatch
    print subprocess.check_output(['sbatch','-C','jimgray84','--mem','10000',script_fn],
                                  stderr=subprocess.STDOUT),'\n\n'
```

In [3]:

```
pairs={'S12': 'S15','S27':'S30'}
for tum,norm in pairs.iteritems():
    run_samt_mp_varscan_som(tum,norm,input_dir,output_dir,ref_genome=galref)
    run_samt_mp_varscan_som(norm,tum,input_dir,output_dir,ref_genome=galref)
```

---

### Running VarScan processSomatic (step 3)¶

In [4]:

```
def run_varscan_procsom(tum_sample,norm_sample,output_dir,ref_genome):
    #output file
    input_base=output_dir+'/'+tum_sample+'_'+norm_sample+'.vsc'

    #snp
    #varscan command
    cmd='time java -jar VarScan.v2.3.7.jar processSomatic '+ input_base +'.snp'
    print cmd,'\n'

    #write scriptfile for sbatch
    script_fn=tum_sample+'_'+norm_sample+'_ps_snp.sh'
    with open(script_fn,'w') as f:
        f.write('#!/bin/bash\n'+cmd+'\n')
    
    #submit script to sbatch
    print subprocess.check_output(['sbatch','-C','jimgray84','--mem','10000',script_fn],
                                  stderr=subprocess.STDOUT),'\n\n'
        
    #indel
    #varscan command
    cmd='time java -jar VarScan.v2.3.7.jar processSomatic '+ input_base +'.indel'
    print cmd,'\n'

    #write scriptfile for sbatch
    script_fn=tum_sample+'_'+norm_sample+'_ps_indel.sh'
    with open(script_fn,'w') as f:
        f.write('#!/bin/bash\n'+cmd+'\n')
    
    #submit script to sbatch
    print subprocess.check_output(['sbatch','-C','jimgray84','--mem','10000',script_fn],
                                  stderr=subprocess.STDOUT),'\n\n'
```

In [5]:

```
for tum,norm in pairs.iteritems():
    run_varscan_procsom(tum,norm,output_dir,ref_genome=galref)
    run_varscan_procsom(norm,tum,output_dir,ref_genome=galref)
```

---

### Running somaticFilter (step 4)¶

In [6]:

```
def run_varscan_somfilt(tum_sample,norm_sample,output_dir,ref_genome):
    #output file
    input_base=tum_sample+'_'+norm_sample+'.vsc'

    #varscan command
    cmd='time java -jar VarScan.v2.3.7.jar somaticFilter '
    cmd+=input_base+'.snp.Somatic.hc --indel-file ' + input_base+'.indel'
    cmd+=' --output-file ' + input_base+'.snp.Somatic.hc.filter'
    print cmd,'\n'

    #write scriptfile for sbatch
    script_fn=tum_sample+'_'+norm_sample+'_somfilt.sh'
    with open(script_fn,'w') as f:
        f.write('#!/bin/bash\n'+cmd+'\n')
    
    #submit script to sbatch
    print subprocess.check_output(['sbatch','-C','jimgray84','--mem','10000',script_fn],
                                  stderr=subprocess.STDOUT),'\n\n'
```

In [7]:

```
for tum,norm in pairs.iteritems():
    run_varscan_somfilt(tum,norm,output_dir,ref_genome=galref)
    run_varscan_somfilt(norm,tum,output_dir,ref_genome=galref)
```

---

### Running bamcount (step +1)¶

- github: https://github.com/genome/bam-readcount/blob/master/README.textile

#### Creating bed files for positions¶

In [8]:

```
def create_beds(tum_sample,norm_sample):
    variant_file=tum_sample+'_'+norm_sample+'.vsc.snp.Somatic.hc.filter'
    
    cmd='tail -n+2 '+variant_file+' | awk \'{print $1"\t"$2"\t"$2}\' > '
    cmd+=variant_file+'.bed'
    
    print subprocess.check_output(cmd,shell=True),
    
for tum,norm in pairs.iteritems():
    create_beds(tum,norm)
    create_beds(norm,tum)
```

```

```

#### Running bamcount¶

In [9]:

```
def run_bamcount(tum_sample,norm_sample,input_dir,ref_genome):
    #output file
    variant_file=tum_sample+'_'+norm_sample+'.vsc.snp.Somatic.hc.filter.bed'
    bam_file=input_dir+tum_sample+'.bam'

    #command
    cmd='~/tools/bam-readcount_build/bin/bam-readcount -q 1 -b 20'
    cmd+=' -f ' + ref_genome + ' -l ' + variant_file +' '
    cmd+=  bam_file + ' > ' +variant_file+'.readcounts'
    print cmd,'\n'

    #write scriptfile for sbatch
    script_fn=tum_sample+'_'+norm_sample+'_bamcount.sh'
    with open(script_fn,'w') as f:
        f.write('#!/bin/bash\n'+cmd+'\n')
    
    #submit script to sbatch
    print subprocess.check_output(['sbatch','-C','jimgray84','--mem','2000',script_fn],
                                  stderr=subprocess.STDOUT),'\n\n'
```

In [10]:

```
for tum,norm in pairs.iteritems():
    run_bamcount(tum,norm,input_dir,ref_genome=galref)
    run_bamcount(norm,tum,input_dir,ref_genome=galref)
```

---

### Running false positive filter (step +2)¶

In [11]:

```
def run_fpfilter(tum_sample,norm_sample):
    #output file
    variant_file=tum_sample+'_'+norm_sample+'.vsc.snp.Somatic.hc.filter'

    #command
    cmd='perl ~/tools/VarScan/fpfilter.pl '+ variant_file +' '
    cmd+= variant_file+'.bed.readcounts '
    cmd+=' -output-basename '+variant_file+'.fpfilter '
    print cmd,'\n'

    #write scriptfile for sbatch
    script_fn=tum_sample+'_'+norm_sample+'_fpfilter.sh'
    with open(script_fn,'w') as f:
        f.write('#!/bin/bash\n'+cmd+'\n')
    
    #submit script to sbatch
    print subprocess.check_output(['sbatch','-C','jimgray84','--mem','2000',script_fn],
                                  stderr=subprocess.STDOUT),'\n\n'
```

In [12]:

```
for tum,norm in pairs.iteritems():
    run_fpfilter(tum,norm)
    run_fpfilter(norm,tum)
```

---

## Interpreting the results¶

In [13]:

```
import pandas as pd
import numpy as np
import matplotlib.pyplot as plt
%matplotlib inline
```

#### Loading results¶

In [14]:

```
header=pd.read_csv('S27_S30.vsc.snp.Somatic.hc.filter',sep='\t').columns

df_dict=dict()
for tum,norm in pairs.iteritems():
    df_dict[tum]=pd.read_csv(tum+'_'+norm+'.vsc.snp.Somatic.hc.filter.fpfilter.pass',
                             sep='\t',header=None)
    df_dict[tum].columns=header
    df_dict[norm]=pd.read_csv(norm+'_'+tum+'.vsc.snp.Somatic.hc.filter.fpfilter.pass',
                              sep='\t',header=None)
    df_dict[norm].columns=header
```

#### Filtering out any scaffolds¶

In [15]:

```
chroms=set(map(str,range(1,28)+[32]) + ['W','Z'])

for key,table in df_dict.iteritems():
    df_dict[key]=table[np.array([x in chroms for x in table['chrom']])]
```

### Final mutation counts in identical samples (false positives)¶

In [16]:

```
for key,table in df_dict.iteritems():
    print key,len(table)
```

```
S12 368
S27 1264
S15 410
S30 922
```

#### A few examples of falsely identified somatic mutations¶

In [17]:

```
df_dict['S27'].head()
```

Out[17]:

|  | chrom | position | ref | var | normal\_reads1 | normal\_reads2 | normal\_var\_freq | normal\_gt | tumor\_reads1 | tumor\_reads2 | ... | variant\_p\_value | somatic\_p\_value | tumor\_reads1\_plus | tumor\_reads1\_minus | tumor\_reads2\_plus | tumor\_reads2\_minus | normal\_reads1\_plus | normal\_reads1\_minus | normal\_reads2\_plus | normal\_reads2\_minus |
| --- | --- | --- | --- | --- | --- | --- | --- | --- | --- | --- | --- | --- | --- | --- | --- | --- | --- | --- | --- | --- | --- |
| 0 | 1 | 109 | T | C | 37 | 0 | 0% | T | 21 | 8 | ... | 1 | 7.472954e-04 | 21 | 0 | 8 | 0 | 37 | 0 | 0 | 0 |
| 1 | 1 | 29243 | A | G | 25 | 0 | 0% | A | 36 | 14 | ... | 1 | 1.672756e-03 | 5 | 31 | 11 | 3 | 4 | 21 | 0 | 0 |
| 2 | 1 | 3982436 | C | A | 43 | 2 | 4.44% | C | 78 | 21 | ... | 1 | 7.063081e-03 | 68 | 10 | 20 | 1 | 34 | 9 | 1 | 1 |
| 3 | 1 | 3983933 | G | A | 24 | 0 | 0% | G | 11 | 44 | ... | 1 | 3.673837e-12 | 2 | 9 | 2 | 42 | 10 | 14 | 0 | 0 |
| 4 | 1 | 3993862 | G | T | 111 | 3 | 2.63% | G | 121 | 38 | ... | 1 | 1.888714e-07 | 77 | 44 | 23 | 15 | 85 | 26 | 0 | 3 |

5 rows × 23 columns

In [18]:

```
df_dict['S30'].head()
```

Out[18]:

|  | chrom | position | ref | var | normal\_reads1 | normal\_reads2 | normal\_var\_freq | normal\_gt | tumor\_reads1 | tumor\_reads2 | ... | variant\_p\_value | somatic\_p\_value | tumor\_reads1\_plus | tumor\_reads1\_minus | tumor\_reads2\_plus | tumor\_reads2\_minus | normal\_reads1\_plus | normal\_reads1\_minus | normal\_reads2\_plus | normal\_reads2\_minus |
| --- | --- | --- | --- | --- | --- | --- | --- | --- | --- | --- | --- | --- | --- | --- | --- | --- | --- | --- | --- | --- | --- |
| 0 | 1 | 253804 | G | A | 11 | 0 | 0% | G | 1 | 8 | ... | 1 | 0.000071 | 0 | 1 | 6 | 2 | 6 | 5 | 0 | 0 |
| 1 | 1 | 428312 | C | T | 51 | 1 | 1.92% | C | 18 | 8 | ... | 1 | 0.000463 | 1 | 17 | 0 | 8 | 19 | 32 | 0 | 1 |
| 2 | 1 | 2699491 | T | C | 43 | 2 | 4.44% | T | 19 | 5 | ... | 1 | 0.044937 | 12 | 7 | 2 | 3 | 20 | 23 | 0 | 2 |
| 3 | 1 | 2742493 | A | G | 65 | 1 | 1.52% | A | 11 | 6 | ... | 1 | 0.000201 | 11 | 0 | 6 | 0 | 35 | 30 | 1 | 0 |
| 4 | 1 | 3963914 | G | T | 20 | 0 | 0% | G | 56 | 15 | ... | 1 | 0.016682 | 56 | 0 | 15 | 0 | 20 | 0 | 0 | 0 |

5 rows × 23 columns

In [19]:

```
df_dict['S12'].head()
```

Out[19]:

|  | chrom | position | ref | var | normal\_reads1 | normal\_reads2 | normal\_var\_freq | normal\_gt | tumor\_reads1 | tumor\_reads2 | ... | variant\_p\_value | somatic\_p\_value | tumor\_reads1\_plus | tumor\_reads1\_minus | tumor\_reads2\_plus | tumor\_reads2\_minus | normal\_reads1\_plus | normal\_reads1\_minus | normal\_reads2\_plus | normal\_reads2\_minus |
| --- | --- | --- | --- | --- | --- | --- | --- | --- | --- | --- | --- | --- | --- | --- | --- | --- | --- | --- | --- | --- | --- |
| 0 | 1 | 160242 | T | C | 16 | 0 | 0% | T | 17 | 6 | ... | 1 | 0.030940 | 8 | 9 | 3 | 3 | 7 | 9 | 0 | 0 |
| 1 | 1 | 3987951 | T | A | 25 | 1 | 3.85% | T | 21 | 10 | ... | 1 | 0.006709 | 9 | 12 | 1 | 9 | 9 | 16 | 0 | 1 |
| 2 | 1 | 5423627 | A | G | 23 | 0 | 0% | A | 16 | 5 | ... | 1 | 0.018737 | 8 | 8 | 2 | 3 | 8 | 15 | 0 | 0 |
| 3 | 1 | 8545137 | C | A | 24 | 0 | 0% | C | 9 | 4 | ... | 1 | 0.010826 | 4 | 5 | 1 | 3 | 11 | 13 | 0 | 0 |
| 4 | 1 | 11647336 | A | C | 11 | 0 | 0% | A | 7 | 7 | ... | 1 | 0.007140 | 3 | 4 | 3 | 4 | 3 | 8 | 0 | 0 |

5 rows × 23 columns

In [20]:

```
df_dict['S15'].head()
```

Out[20]:

|  | chrom | position | ref | var | normal\_reads1 | normal\_reads2 | normal\_var\_freq | normal\_gt | tumor\_reads1 | tumor\_reads2 | ... | variant\_p\_value | somatic\_p\_value | tumor\_reads1\_plus | tumor\_reads1\_minus | tumor\_reads2\_plus | tumor\_reads2\_minus | normal\_reads1\_plus | normal\_reads1\_minus | normal\_reads2\_plus | normal\_reads2\_minus |
| --- | --- | --- | --- | --- | --- | --- | --- | --- | --- | --- | --- | --- | --- | --- | --- | --- | --- | --- | --- | --- | --- |
| 0 | 1 | 1113695 | A | G | 21 | 1 | 4.55% | A | 20 | 12 | ... | 1 | 0.004796 | 10 | 10 | 8 | 4 | 12 | 9 | 0 | 1 |
| 1 | 1 | 2737118 | C | T | 21 | 0 | 0% | C | 17 | 5 | ... | 1 | 0.027357 | 0 | 17 | 0 | 5 | 0 | 21 | 0 | 0 |
| 2 | 1 | 2742610 | C | G | 35 | 1 | 2.78% | C | 24 | 6 | ... | 1 | 0.030062 | 9 | 15 | 5 | 1 | 14 | 21 | 0 | 1 |
| 3 | 1 | 2745315 | C | T | 38 | 2 | 5% | C | 27 | 7 | ... | 1 | 0.045011 | 15 | 12 | 4 | 3 | 26 | 12 | 2 | 0 |
| 4 | 1 | 3979595 | C | T | 21 | 0 | 0% | C | 19 | 5 | ... | 1 | 0.034789 | 5 | 14 | 1 | 4 | 4 | 17 | 0 | 0 |

5 rows × 23 columns

---

## Plotting the number of false positive mutations for different somatic-p-value thresholds¶

In [21]:

```
pvals=dict()
for key,table in df_dict.iteritems():
    pvals[key]=np.sort(table['somatic_p_value'].values)
```

#### Log-lin scale¶

In [24]:

```
fig,ax=plt.subplots()
fig.set_size_inches(12,9)

for key,value in pvals.iteritems():
    ax.plot(value,np.arange(len(value)),lw=2,label=key)

ax.axvline(0.05,c='m',linestyle='dotted',lw=5,label='varscan default = 0.05')
ax.axvline(0.008,c='m',linestyle='dashed',lw=5,label='used by Rieber et al. = 0.008')

ax.set_xlabel(r'Somatic p-value threshold',fontsize=16)
ax.set_ylabel(r'False mutations found',fontsize=16)


ax.set_xlim(0.1,5e-5)
ax.set_ylim(0,1500)
ax.set_xscale('log')
ax.grid()
dump=ax.legend(loc='upper right',fancybox='true',fontsize=16)
```

#### Log-log scale¶

In [23]:

```
fig,ax=plt.subplots()
fig.set_size_inches(12,9)

for key,value in pvals.iteritems():
    ax.plot(value,np.arange(len(value)),lw=2,label=key)

ax.axvline(0.05,c='m',linestyle='dotted',lw=5,label='varscan default = 0.05')
ax.axvline(0.008,c='m',linestyle='dashed',lw=5,label='used by Rieber et al. = 0.008')

ax.set_xlabel(r'Somatic p-value threshold',fontsize=16)
ax.set_ylabel(r'False mutations found',fontsize=16)


ax.set_xlim(0.1,5e-20)
ax.set_ylim(1,1000)
ax.set_xscale('log')
ax.set_yscale('log')
ax.grid()
dump=ax.legend(loc='upper right',fancybox='true',fontsize=16)
```

---

# Conclusions:¶

#### With both the default VarScan settings, and another proposed p-value limit the number of FP mutations remains high.¶

- Default settings result in 400-1000 FP mutations per sample
- The proposed p-value limit results in 150-600 FP mutations per sample
- To decrease the number of false positives to a maximum of 10 per sample, the p-value would have to be decreased to 1e-18. As the p parameter is related to an actual probability, this threshold values is absurd and no real mutations in other samples could be found with it either.

#### This deficiency on our dataset is probably due to the fact that VarScan relies on other FP filtering methods which is available for human sequences, but not for our analyzed dataset:¶

- dbSNP
- Repeat masking

---

# References:¶

- Varscan 2: http://www.ncbi.nlm.nih.gov/pubmed/22300766
- best practice: http://www.ncbi.nlm.nih.gov/pmc/articles/PMC4278659/?tool=pmcentrez
- Somatic p-value theshold: http://journals.plos.org/plosone/article?id=10.1371/journal.pone.0066621
