# Supplementary material for: Fast and accurate mutation detection in whole genome sequences of multiple isogenic samples with IsoMut
Source: BMC Bioinformatics. 2017 Jan 31;18:73. doi: 10.1186/s12859-017-1492-4 (PMC5282906; doi:10.1186/s12859-017-1492-4)
Supplement: Additional file 9: — Running MuTect on our dataset with default settings. Computational details and results of running MuTect on the described dataset without the tuning of the LOD parameter. (HTML 675 kb) [file 12859_2017_1492_MOESM9_ESM.html]

MuTect\_pipeline\_tum\_norm\_pair\_and\_PoN\_WT\_KO


# Additional file 9 - Running MuTect on a dataset of 30 samples with the optimization of the LOD parameter¶

### Fast and accurate mutation detection in whole genome sequences of multiple isogenic samples with IsoMut¶

##### O. Pipek, D. Ribli, J. Molnár, Á. Póti, M. Krzystanek, A. Bodor, G. E. Tusnády, Z. Szallasi, I. Csabai, and D. Szüts¶

---

## MuTect somatic mutation calling pipeline with a tumor-normal pair and a Panel of Normal samples¶

- In depth discussion of the method used by MuTect can be found in the original article by Cibulskis et al. (http://www.nature.com/nbt/journal/v31/n3/full/nbt.2514.html).
- For a step-by-step description about the usage of the software, the guidelines of the following presentations were used: https://www.broadinstitute.org/gatk/guide/presentations?id=6007
- Other guides are also available here: https://www.broadinstitute.org/gatk/guide/

#### Details of the method:¶

- In the first step we used a tumor-normal pair and also many other normal samples (as the Panel of Normals) for SNV detection, as suggested by [https://www.broadinstitute.org/gatk/events/slides/1506/GATKwr8-S-3-Variant\_calling\_with\_MuTect.pdf].
- As the default settings did not give sufficient results, the LOD parameter (which is related the quality of a mutation call) was tuned to decrease false positive rates.

#### Assessing the performance of the method:¶

- An ideal method would **minimize false positives** (all mutations in the control samples), while still maintaining **high mutation rates in treated samples** (even though these are not necessarily true positives!).
- These two values can to some extent describe the performance of a unique mutation detection method and can be used to also compare these. Also, by using these two values as reference, tunable parameters of any method can be optimized.

---

## Preparation¶

---

#### Loading modules¶

In [1]:

```
#load modules

import os
import subprocess
import time

import matplotlib
import matplotlib.pyplot as plt
%matplotlib inline

import numpy as np
import pandas as pd

#go to working directory
work_dir='/nagyvinyok/adat84/sotejedlik/ribli/dt40/method/gatk/mutect_pair_pon_wt_ko'
subprocess.call(['mkdir',work_dir])
os.chdir(work_dir)
```

#### Setting directories and paths¶

In [2]:

```
#gallus reference
galref="/home/ribli/input/index/gallus/complete/Gallus_gallus.Galgal4.74.dna.toplevel.fa"
input_dir='/nagyvinyok/adat83/sotejedlik/orsi/bam_all_links_methodpaper/'
output_dir=work_dir
```

---

## Creating Panel of Normal samples (PoN)¶

---

#### Crating the file format necessary for running gatk¶

In [3]:

```
%%bash
java -jar /nagyvinyok/adat88/kozos/sotejedlik/usr/gatk/picard.jar \
    CreateSequenceDictionary \
    REFERENCE=/home/ribli/input/index/gallus/complete/Gallus_gallus.Galgal4.74.dna.toplevel.fa   \
    OUTPUT=/home/ribli/input/index/gallus/complete/Gallus_gallus.Galgal4.74.dna.toplevel.dict
```

#### Creating vcf files for each sample of the PoN¶

- Defining a function to run the generation of vcf-s needed for PoN
- Running the function

  - It runs for around 12 hours per sample

In [5]:

```
def run_mutect_artif_detect(sample,input_dir,output_dir,ref_genome):
    
    #input,output,log,scipt_file
    sample_bam=input_dir+sample+'_RMdup_picard_realign.bam '
    log=output_dir+'/'+sample+'.gatkVcf.log '
    script_fname=sample+'gatk1'+'.sh'

    #gatk command
    cmd='time java -Xmx2g -jar /nagyvinyok/adat88/kozos/sotejedlik/usr/gatk/mutect-1.1.7.jar '
    cmd+=' -T MuTect ' 
    cmd+=' -R '+ ref_genome
    cmd+=' -I:tumor '+ sample_bam
    cmd+=' --artifact_detection_mode '
    cmd+=' -vcf ' + sample + '.call_stats.vcf'
    cmd+=' --coverage_file '+sample+'.coverage.wig.txt'
    cmd+=' 2> '+ log
    print cmd,'\n'

    #write scriptfile
    with open(script_fname,'w') as f:
        f.write('#!/bin/bash\n')
        f.write(cmd+'\n')

    #submit script to sbatch
    print subprocess.check_output(['sbatch','--mem','3000',script_fname],
                                  stderr=subprocess.STDOUT),'\n'

    
    
#########################################################################   
samples=['S01','S02','S03','S04','S05','S06','S07','S08',
         'S09','S10','S11','S12','S13','S14','S15']
samples+=['S16','S17','S18','S19','S20','S21','S22','S23',
         'S24','S25','S26','S27','S28','S29','S30']

for sample in samples:
    run_mutect_artif_detect(sample,input_dir,output_dir,ref_genome=galref)
```

#### Checking for errors¶

In [14]:

```
%%bash
tail -n1 *Vcf.log
```

```
==> DS001.gatkVcf.log <==
INFO  22:08:22,602 MicroScheduler -   -> 646880 reads (0.35% of total) failing UnmappedReadFilter 

==> DS003.gatkVcf.log <==
INFO  21:39:14,096 MicroScheduler -   -> 988436 reads (0.62% of total) failing UnmappedReadFilter 

==> DS004.gatkVcf.log <==
INFO  22:03:26,734 MicroScheduler -   -> 1456490 reads (0.81% of total) failing UnmappedReadFilter 

==> DS006.gatkVcf.log <==
INFO  22:03:36,283 MicroScheduler -   -> 838120 reads (0.47% of total) failing UnmappedReadFilter 

==> DS014.gatkVcf.log <==
INFO  23:42:16,413 MicroScheduler -   -> 963247 reads (0.47% of total) failing UnmappedReadFilter 

==> DS015.gatkVcf.log <==
INFO  22:46:49,966 MicroScheduler -   -> 1567341 reads (0.94% of total) failing UnmappedReadFilter 

==> DS016.gatkVcf.log <==
INFO  22:27:41,533 MicroScheduler -   -> 932658 reads (0.57% of total) failing UnmappedReadFilter 

==> DS018.gatkVcf.log <==
INFO  22:41:04,016 MicroScheduler -   -> 824751 reads (0.49% of total) failing UnmappedReadFilter 

==> DS041.gatkVcf.log <==
INFO  21:38:48,257 MicroScheduler -   -> 802607 reads (0.42% of total) failing UnmappedReadFilter 

==> DS042.gatkVcf.log <==
INFO  22:47:36,185 MicroScheduler -   -> 1034327 reads (0.45% of total) failing UnmappedReadFilter 

==> DS043.gatkVcf.log <==
INFO  21:37:06,709 MicroScheduler -   -> 818829 reads (0.43% of total) failing UnmappedReadFilter 

==> DS044.gatkVcf.log <==
INFO  21:49:32,587 MicroScheduler -   -> 909005 reads (0.45% of total) failing UnmappedReadFilter 

==> DS045.gatkVcf.log <==
INFO  21:54:52,159 MicroScheduler -   -> 506513 reads (0.24% of total) failing UnmappedReadFilter 

==> DS046.gatkVcf.log <==
INFO  03:08:09,459 MicroScheduler -   -> 483485 reads (0.22% of total) failing UnmappedReadFilter 

==> DS047.gatkVcf.log <==
INFO  07:48:33,010 MicroScheduler -   -> 1282658 reads (0.42% of total) failing UnmappedReadFilter 

==> DS048.gatkVcf.log <==
INFO  22:30:41,111 MicroScheduler -   -> 583946 reads (0.26% of total) failing UnmappedReadFilter 

==> DS049.gatkVcf.log <==
INFO  22:10:49,754 MicroScheduler -   -> 811987 reads (0.37% of total) failing UnmappedReadFilter 

==> DS050.gatkVcf.log <==
INFO  22:43:43,708 MicroScheduler -   -> 757247 reads (0.32% of total) failing UnmappedReadFilter 

==> DS051.gatkVcf.log <==
INFO  22:50:57,744 MicroScheduler -   -> 748720 reads (0.38% of total) failing UnmappedReadFilter 

==> DS052.gatkVcf.log <==
INFO  23:21:31,137 MicroScheduler -   -> 815399 reads (0.39% of total) failing UnmappedReadFilter 

==> DS053.gatkVcf.log <==
INFO  17:29:54,936 MicroScheduler -   -> 471201 reads (0.24% of total) failing UnmappedReadFilter 

==> DS054.gatkVcf.log <==
INFO  18:49:27,806 MicroScheduler -   -> 526384 reads (0.22% of total) failing UnmappedReadFilter 

==> DS055.gatkVcf.log <==
INFO  18:42:12,069 MicroScheduler -   -> 542802 reads (0.24% of total) failing UnmappedReadFilter 

==> DS056.gatkVcf.log <==
INFO  17:44:21,214 MicroScheduler -   -> 458026 reads (0.23% of total) failing UnmappedReadFilter 

==> DS057.gatkVcf.log <==
INFO  23:41:28,322 MicroScheduler -   -> 1381482 reads (0.43% of total) failing UnmappedReadFilter 

==> DS058.gatkVcf.log <==
INFO  02:14:03,069 MicroScheduler -   -> 1607180 reads (0.41% of total) failing UnmappedReadFilter 

==> DS097.gatkVcf.log <==
INFO  23:08:02,701 MicroScheduler -   -> 464610 reads (0.19% of total) failing UnmappedReadFilter 

==> DS101.gatkVcf.log <==
INFO  19:15:40,977 MicroScheduler -   -> 357651 reads (0.14% of total) failing UnmappedReadFilter 

==> DS102.gatkVcf.log <==
INFO  19:05:28,549 MicroScheduler -   -> 413614 reads (0.17% of total) failing UnmappedReadFilter 

==> DS103.gatkVcf.log <==
INFO  22:13:19,378 MicroScheduler -   -> 417924 reads (0.12% of total) failing UnmappedReadFilter
```

## Combining vcf files into PoN¶

- a different PoN is used for the mutation detection of each sample
- running this in parallel on our system results in various errors:
  - java i/o error
  - malformed lines produced (that cause errors later when using them)

#### Defining function for the generation of PoN¶

- The guidelines used described the PoN generation without using the --genotypemergeoption UNIQUIFY option. However, this resulted in the following error message:

  - *Duplicate sample names were discovered but no genotypemergeoption was supplied. To combine samples without merging specify --genotypemergeoption UNIQUIFY. Merging duplicate samples without specified priority is unsupported, but can be achieved by specifying --genotypemergeoption UNSORTED.*
- To overcome the issue, the --genotypemergeoption UNIQUIFY option was invoked.

In [ ]:

```
def run_mutect_cobine_into_pon(sample,samples,input_dir,ref_genome):
    
    #input,output,log,scipt_file
    output=sample+'_PoN.vcf'
    log=sample+'.gatkCombine.log '
    script_fname=sample+'_PoN_combine.sh'

    #gatk command
    cmd='time java -Xmx3g -jar /nagyvinyok/adat88/kozos/sotejedlik/usr/gatk/GenomeAnalysisTK.jar '
    cmd+=' -T CombineVariants ' 
    cmd+=' -R '+ ref_genome
    for other_sample in samples:
        if (other_sample != sample):
            cmd+=' -V '+other_sample+'.call_stats.vcf'
    cmd+=' -minN 2 '
    cmd+=' --filteredrecordsmergetype KEEP_IF_ANY_UNFILTERED '
    cmd+=' --filteredAreUncalled '
    cmd+=' --genotypemergeoption UNIQUIFY '
    cmd+=' -o ' + output
    cmd+=' 2> '+ log
    print cmd,'\n'

    #write scriptfile
    with open(script_fname,'w') as f:
        f.write('#!/bin/bash\n')
        f.write(cmd+'\n')

    #submit script to sbatch
    print subprocess.check_output(['sbatch','-C','jimgray83','--mem','3000',script_fname],
                                  stderr=subprocess.STDOUT),'\n'

    
#########################################################################    
samples=['S01','S02','S03','S04','S05','S06','S07','S08',
         'S09','S10','S11','S12','S13','S14','S15']
samples+=['S16','S17','S18','S19','S20','S21','S22','S23',
         'S24','S25','S26','S27','S28','S29','S30']

for sample in samples:
    run_mutect_cobine_into_pon(sample,samples,input_dir,ref_genome=galref)
```

#### Checking for errors¶

In [5]:

```
%%bash
tail -n2 *Combine.log
```

```
==> DS001.gatkCombine.log <==
INFO  16:55:46,810 ProgressMeter - Total runtime 6281.28 secs, 104.69 min, 1.74 hours 
INFO  16:56:57,130 GATKRunReport - Uploaded run statistics report to AWS S3 

==> DS003.gatkCombine.log <==
INFO  16:49:14,217 ProgressMeter - Total runtime 5827.09 secs, 97.12 min, 1.62 hours 
INFO  16:49:34,176 GATKRunReport - Uploaded run statistics report to AWS S3 

==> DS004.gatkCombine.log <==
INFO  16:47:47,911 ProgressMeter - Total runtime 5738.95 secs, 95.65 min, 1.59 hours 
INFO  16:48:49,245 GATKRunReport - Uploaded run statistics report to AWS S3 

==> DS006.gatkCombine.log <==
INFO  16:47:48,817 ProgressMeter - Total runtime 5738.95 secs, 95.65 min, 1.59 hours 
INFO  16:48:46,904 GATKRunReport - Uploaded run statistics report to AWS S3 

==> DS014.gatkCombine.log <==
INFO  20:01:40,027 ProgressMeter - Total runtime 9106.35 secs, 151.77 min, 2.53 hours 
INFO  20:02:58,736 GATKRunReport - Uploaded run statistics report to AWS S3 

==> DS015.gatkCombine.log <==
INFO  21:01:40,622 ProgressMeter - Total runtime 6924.93 secs, 115.42 min, 1.92 hours 
INFO  21:02:02,256 GATKRunReport - Uploaded run statistics report to AWS S3 

==> DS016.gatkCombine.log <==
INFO  21:01:14,879 ProgressMeter - Total runtime 6899.79 secs, 115.00 min, 1.92 hours 
INFO  21:01:48,347 GATKRunReport - Uploaded run statistics report to AWS S3 

==> DS018.gatkCombine.log <==
INFO  21:01:09,006 ProgressMeter - Total runtime 6893.76 secs, 114.90 min, 1.91 hours 
INFO  21:01:49,395 GATKRunReport - Uploaded run statistics report to AWS S3 

==> DS041.gatkCombine.log <==
INFO  16:45:55,616 ProgressMeter - Total runtime 5565.53 secs, 92.76 min, 1.55 hours 
INFO  16:46:02,085 GATKRunReport - Uploaded run statistics report to AWS S3 

==> DS042.gatkCombine.log <==
INFO  16:47:46,983 ProgressMeter - Total runtime 5675.58 secs, 94.59 min, 1.58 hours 
INFO  16:48:43,937 GATKRunReport - Uploaded run statistics report to AWS S3 

==> DS043.gatkCombine.log <==
INFO  17:05:04,261 ProgressMeter - Total runtime 6203.05 secs, 103.38 min, 1.72 hours 
INFO  17:05:56,455 GATKRunReport - Uploaded run statistics report to AWS S3 

==> DS044.gatkCombine.log <==
INFO  17:28:18,685 ProgressMeter - Total runtime 6834.15 secs, 113.90 min, 1.90 hours 
INFO  17:28:47,839 GATKRunReport - Uploaded run statistics report to AWS S3 

==> DS045.gatkCombine.log <==
INFO  19:04:16,535 ProgressMeter - Total runtime 8230.15 secs, 137.17 min, 2.29 hours 
INFO  19:04:41,211 GATKRunReport - Uploaded run statistics report to AWS S3 

==> DS046.gatkCombine.log <==
INFO  19:04:19,288 ProgressMeter - Total runtime 8086.35 secs, 134.77 min, 2.25 hours 
INFO  19:04:34,758 GATKRunReport - Uploaded run statistics report to AWS S3 

==> DS047.gatkCombine.log <==
INFO  19:04:00,764 ProgressMeter - Total runtime 8071.24 secs, 134.52 min, 2.24 hours 
INFO  19:04:14,804 GATKRunReport - Uploaded run statistics report to AWS S3 

==> DS048.gatkCombine.log <==
INFO  19:04:15,027 ProgressMeter - Total runtime 8080.87 secs, 134.68 min, 2.24 hours 
INFO  19:04:35,629 GATKRunReport - Uploaded run statistics report to AWS S3 

==> DS049.gatkCombine.log <==
INFO  19:04:26,635 ProgressMeter - Total runtime 8036.89 secs, 133.95 min, 2.23 hours 
INFO  19:04:38,901 GATKRunReport - Uploaded run statistics report to AWS S3 

==> DS050.gatkCombine.log <==
INFO  19:31:25,203 ProgressMeter - Total runtime 9206.92 secs, 153.45 min, 2.56 hours 
INFO  19:32:46,116 GATKRunReport - Uploaded run statistics report to AWS S3 

==> DS051.gatkCombine.log <==
INFO  21:00:41,587 ProgressMeter - Total runtime 6866.82 secs, 114.45 min, 1.91 hours 
INFO  21:00:56,297 GATKRunReport - Uploaded run statistics report to AWS S3 

==> DS052.gatkCombine.log <==
INFO  21:00:41,601 ProgressMeter - Total runtime 6866.50 secs, 114.44 min, 1.91 hours 
INFO  21:00:56,338 GATKRunReport - Uploaded run statistics report to AWS S3 

==> DS053.gatkCombine.log <==
INFO  21:29:09,575 ProgressMeter - Total runtime 6863.61 secs, 114.39 min, 1.91 hours 
INFO  21:29:25,275 GATKRunReport - Uploaded run statistics report to AWS S3 

==> DS054.gatkCombine.log <==
INFO  21:27:32,505 ProgressMeter - Total runtime 6482.35 secs, 108.04 min, 1.80 hours 
INFO  21:27:56,873 GATKRunReport - Uploaded run statistics report to AWS S3 

==> DS055.gatkCombine.log <==
INFO  21:48:27,952 ProgressMeter - Total runtime 6273.55 secs, 104.56 min, 1.74 hours 
INFO  21:48:34,416 GATKRunReport - Uploaded run statistics report to AWS S3 

==> DS056.gatkCombine.log <==
INFO  22:20:54,027 ProgressMeter - Total runtime 4733.47 secs, 78.89 min, 1.31 hours 
INFO  22:21:15,576 GATKRunReport - Uploaded run statistics report to AWS S3 

==> DS057.gatkCombine.log <==
INFO  22:20:49,176 ProgressMeter - Total runtime 4728.62 secs, 78.81 min, 1.31 hours 
INFO  22:21:13,633 GATKRunReport - Uploaded run statistics report to AWS S3 

==> DS058.gatkCombine.log <==
INFO  22:20:37,247 ProgressMeter - Total runtime 4684.65 secs, 78.08 min, 1.30 hours 
INFO  22:21:10,428 GATKRunReport - Uploaded run statistics report to AWS S3 

==> DS097.gatkCombine.log <==
INFO  19:38:30,934 ProgressMeter - Total runtime 9069.66 secs, 151.16 min, 2.52 hours 
INFO  19:38:38,728 GATKRunReport - Uploaded run statistics report to AWS S3 

==> DS101.gatkCombine.log <==
INFO  22:20:36,000 ProgressMeter - Total runtime 4674.66 secs, 77.91 min, 1.30 hours 
INFO  22:21:10,428 GATKRunReport - Uploaded run statistics report to AWS S3 

==> DS102.gatkCombine.log <==
INFO  22:20:36,026 ProgressMeter - Total runtime 4674.36 secs, 77.91 min, 1.30 hours 
INFO  22:21:10,457 GATKRunReport - Uploaded run statistics report to AWS S3 

==> DS103.gatkCombine.log <==
INFO  22:33:34,053 ProgressMeter - Total runtime 3880.52 secs, 64.68 min, 1.08 hours 
INFO  22:33:39,364 GATKRunReport - Uploaded run statistics report to AWS S3
```

---

## Running MuTect with a tumor-normal pair and the PoN generated above¶

---

#### Defining function for the execution of MuTect¶

In [ ]:

```
def run_mutect_w_pair_and_pon(sample,normal_sample,input_dir,ref_genome):
    
    #input,output,log,scipt_file
    normal_sample_bam=input_dir+normal_sample+'_RMdup_picard_realign.bam '
    sample_bam=input_dir+sample+'_RMdup_picard_realign.bam '
    pon_file= sample+'_PoN.vcf'
    cov_file= sample+'_w_pair_and_PoN.coverage.wig.txt'
    output= sample+'_w_pair_and_PoN.call_stats.txt'
    log=sample+'.mutect_w_pair_and_PoN.log'
    script_fname=sample+'_w_pair_and_PoN.sh'

    #gatk command
    cmd='time java -Xmx3g -jar /nagyvinyok/adat88/kozos/sotejedlik/usr/gatk/mutect-1.1.7.jar '
    cmd+=' -T MuTect ' 
    cmd+=' -R '+ ref_genome
    cmd+=' -I:normal '+ normal_sample_bam
    cmd+=' -I:tumor '+ sample_bam
    cmd+=' --normal_panel '+ pon_file
    cmd+=' --coverage_file ' + cov_file
    cmd+=' -o ' + output
    cmd+=' 2> '+ log
    print cmd,'\n'

    #write scriptfile
    with open(script_fname,'w') as f:
        f.write('#!/bin/bash\n')
        f.write(cmd+'\n')

    #submit script to sbatch
    print subprocess.check_output(['sbatch','-C','jimgray83','--mem','3000',script_fname],
                                  stderr=subprocess.STDOUT),'\n'
    
    
#########################################################################    
samples=       ['S01','S02','S03','S04','S05','S06','S07',
         'S08','S09','S10','S11','S12','S13','S14','S15']
normal_samples=['S02','S01','S01','S01','S15','S15','S15',
         'S15','S15','S15','S15','S15','S15','S15','S12']

samples+=         ['S16','S17','S18','S19','S20','S21','S22','S23',
                  'S24','S25','S26','S27','S28','S29','S30']
normal_samples+=  ['S18','S16','S16','S16','S21','S20','S20','S20',
                  'S20','S20','S20','S30','S20','S20','S27']


for sample,normal_sample in zip(samples,normal_samples):
    run_mutect_w_pair_and_pon(sample,normal_sample,input_dir,ref_genome=galref)
```

#### Checking for errors¶

- For some samples, errors appeared in scaffolds, but as these parts of the genome are always excluded from our analysis, this does not present a real problem.

In [48]:

```
%%bash
tail -n2 *mutect_w_pair_and_PoN.log
```

```
==> DS001.mutect_w_pair_and_PoN.log <==
INFO  01:34:27,167 MicroScheduler -   -> 0 reads (0.00% of total) failing NotPrimaryAlignmentFilter 
INFO  01:34:27,167 MicroScheduler -   -> 1635316 reads (0.48% of total) failing UnmappedReadFilter 

==> DS003.mutect_w_pair_and_PoN.log <==
INFO  01:36:58,507 MicroScheduler -   -> 0 reads (0.00% of total) failing NotPrimaryAlignmentFilter 
INFO  01:36:58,508 MicroScheduler -   -> 1635316 reads (0.48% of total) failing UnmappedReadFilter 

==> DS004.mutect_w_pair_and_PoN.log <==
INFO  02:07:05,054 MicroScheduler -   -> 0 reads (0.00% of total) failing NotPrimaryAlignmentFilter 
INFO  02:07:05,054 MicroScheduler -   -> 2103370 reads (0.58% of total) failing UnmappedReadFilter 

==> DS006.mutect_w_pair_and_PoN.log <==
INFO  02:07:19,683 MicroScheduler -   -> 0 reads (0.00% of total) failing NotPrimaryAlignmentFilter 
INFO  02:07:19,683 MicroScheduler -   -> 1485000 reads (0.41% of total) failing UnmappedReadFilter 

==> DS014.mutect_w_pair_and_PoN.log <==
INFO  18:18:36,384 MicroScheduler -   -> 0 reads (0.00% of total) failing NotPrimaryAlignmentFilter 
INFO  18:18:36,384 MicroScheduler -   -> 1895905 reads (0.52% of total) failing UnmappedReadFilter 

==> DS015.mutect_w_pair_and_PoN.log <==
INFO  02:32:11,543 MicroScheduler -   -> 0 reads (0.00% of total) failing NotPrimaryAlignmentFilter 
INFO  02:32:11,543 MicroScheduler -   -> 2530588 reads (0.68% of total) failing UnmappedReadFilter 

==> DS016.mutect_w_pair_and_PoN.log <==
INFO  02:10:20,745 MicroScheduler -   -> 0 reads (0.00% of total) failing NotPrimaryAlignmentFilter 
INFO  02:10:20,745 MicroScheduler -   -> 1895905 reads (0.52% of total) failing UnmappedReadFilter 

==> DS018.mutect_w_pair_and_PoN.log <==
INFO  02:19:47,608 MicroScheduler -   -> 0 reads (0.00% of total) failing NotPrimaryAlignmentFilter 
INFO  02:19:47,608 MicroScheduler -   -> 1787998 reads (0.48% of total) failing UnmappedReadFilter 

==> DS041.mutect_w_pair_and_PoN.log <==
INFO  02:30:43,290 MicroScheduler -   -> 0 reads (0.00% of total) failing NotPrimaryAlignmentFilter 
INFO  02:30:43,290 MicroScheduler -   -> 1559854 reads (0.36% of total) failing UnmappedReadFilter 

==> DS042.mutect_w_pair_and_PoN.log <==
INFO  03:38:13,849 MicroScheduler -   -> 0 reads (0.00% of total) failing NotPrimaryAlignmentFilter 
INFO  03:38:13,849 MicroScheduler -   -> 1791574 reads (0.39% of total) failing UnmappedReadFilter 

==> DS043.mutect_w_pair_and_PoN.log <==
INFO  02:21:26,706 MicroScheduler -   -> 0 reads (0.00% of total) failing NotPrimaryAlignmentFilter 
INFO  02:21:26,706 MicroScheduler -   -> 1576076 reads (0.37% of total) failing UnmappedReadFilter 

==> DS044.mutect_w_pair_and_PoN.log <==
INFO  02:50:43,658 MicroScheduler -   -> 0 reads (0.00% of total) failing NotPrimaryAlignmentFilter 
INFO  02:50:43,658 MicroScheduler -   -> 1666252 reads (0.38% of total) failing UnmappedReadFilter 

==> DS045.mutect_w_pair_and_PoN.log <==
INFO  16:29:34,313 MicroScheduler -   -> 0 reads (0.00% of total) failing NotPrimaryAlignmentFilter 
INFO  16:29:34,313 MicroScheduler -   -> 1263760 reads (0.28% of total) failing UnmappedReadFilter 

==> DS046.mutect_w_pair_and_PoN.log <==
INFO  16:53:23,085 MicroScheduler -   -> 0 reads (0.00% of total) failing NotPrimaryAlignmentFilter 
INFO  16:53:23,085 MicroScheduler -   -> 1240732 reads (0.27% of total) failing UnmappedReadFilter 

==> DS047.mutect_w_pair_and_PoN.log <==
INFO  07:43:41,420 MicroScheduler -   -> 0 reads (0.00% of total) failing NotPrimaryAlignmentFilter 
INFO  07:43:41,421 MicroScheduler -   -> 2039905 reads (0.38% of total) failing UnmappedReadFilter 

==> DS048.mutect_w_pair_and_PoN.log <==
INFO  17:42:29,149 MicroScheduler -   -> 0 reads (0.00% of total) failing NotPrimaryAlignmentFilter 
INFO  17:42:29,149 MicroScheduler -   -> 1341193 reads (0.29% of total) failing UnmappedReadFilter 

==> DS049.mutect_w_pair_and_PoN.log <==
INFO  17:32:27,367 MicroScheduler -   -> 0 reads (0.00% of total) failing NotPrimaryAlignmentFilter 
INFO  17:32:27,367 MicroScheduler -   -> 1569234 reads (0.35% of total) failing UnmappedReadFilter 

==> DS050.mutect_w_pair_and_PoN.log <==
INFO  17:11:13,683 MicroScheduler -   -> 0 reads (0.00% of total) failing NotPrimaryAlignmentFilter 
INFO  17:11:13,684 MicroScheduler -   -> 1559854 reads (0.36% of total) failing UnmappedReadFilter 

==> DS051.mutect_w_pair_and_PoN.log <==
INFO  01:39:39,630 MicroScheduler -   -> 0 reads (0.00% of total) failing NotPrimaryAlignmentFilter 
INFO  01:39:39,631 MicroScheduler -   -> 1564119 reads (0.39% of total) failing UnmappedReadFilter 

==> DS052.mutect_w_pair_and_PoN.log <==
INFO  01:49:30,336 MicroScheduler -   -> 0 reads (0.00% of total) failing NotPrimaryAlignmentFilter 
INFO  01:49:30,336 MicroScheduler -   -> 1564119 reads (0.39% of total) failing UnmappedReadFilter 

==> DS053.mutect_w_pair_and_PoN.log <==
INFO  01:37:16,487 MicroScheduler -   -> 0 reads (0.00% of total) failing NotPrimaryAlignmentFilter 
INFO  01:37:16,487 MicroScheduler -   -> 1219921 reads (0.31% of total) failing UnmappedReadFilter 

==> DS054.mutect_w_pair_and_PoN.log <==
INFO  02:23:13,858 MicroScheduler -   -> 0 reads (0.00% of total) failing NotPrimaryAlignmentFilter 
INFO  02:23:13,858 MicroScheduler -   -> 1275104 reads (0.30% of total) failing UnmappedReadFilter 

==> DS055.mutect_w_pair_and_PoN.log <==
INFO  16:01:52,692 MicroScheduler -   -> 0 reads (0.00% of total) failing NotPrimaryAlignmentFilter 
INFO  16:01:52,692 MicroScheduler -   -> 1291522 reads (0.31% of total) failing UnmappedReadFilter 

==> DS056.mutect_w_pair_and_PoN.log <==
INFO  15:20:02,990 MicroScheduler -   -> 0 reads (0.00% of total) failing NotPrimaryAlignmentFilter 
INFO  15:20:02,990 MicroScheduler -   -> 1206746 reads (0.31% of total) failing UnmappedReadFilter 

==> DS057.mutect_w_pair_and_PoN.log <==
INFO  21:01:16,657 MicroScheduler -   -> 0 reads (0.00% of total) failing NotPrimaryAlignmentFilter 
INFO  21:01:16,657 MicroScheduler -   -> 2130202 reads (0.41% of total) failing UnmappedReadFilter 

==> DS058.mutect_w_pair_and_PoN.log <==
##### ERROR MESSAGE: Invalid sequence number 15932 in index file /nagyvinyok/adat83/sotejedlik/ribli/dt40/bam_links_2/DS058_RMdup_picard_realign.bam.bai
##### ERROR ------------------------------------------------------------------------------------------

==> DS097.mutect_w_pair_and_PoN.log <==
##### ERROR MESSAGE: Invalid sequence number 15932 in index file /nagyvinyok/adat83/sotejedlik/ribli/dt40/bam_links_2/DS050_RMdup_picard_realign.bam.bai
##### ERROR ------------------------------------------------------------------------------------------

==> DS101.mutect_w_pair_and_PoN.log <==
##### ERROR MESSAGE: Invalid sequence number 15932 in index file /nagyvinyok/adat83/sotejedlik/ribli/dt40/bam_links_2/DS051_RMdup_picard_realign.bam.bai
##### ERROR ------------------------------------------------------------------------------------------

==> DS102.mutect_w_pair_and_PoN.log <==
##### ERROR MESSAGE: Invalid sequence number 15932 in index file /nagyvinyok/adat83/sotejedlik/ribli/dt40/bam_links_2/DS051_RMdup_picard_realign.bam.bai
##### ERROR ------------------------------------------------------------------------------------------

==> DS103.mutect_w_pair_and_PoN.log <==
##### ERROR MESSAGE: Invalid sequence number 15932 in index file /nagyvinyok/adat83/sotejedlik/ribli/dt40/bam_links_2/DS058_RMdup_picard_realign.bam.bai
##### ERROR ------------------------------------------------------------------------------------------
```

---

## Result:¶

---

#### Understanding the main statistics / fields¶

- Using the description here : http://gatkforums.broadinstitute.org/discussion/4231/what-is-the-output-of-mutect-and-how-should-i-interpret-it

##### Here are the definitions of some of the most prominent outputs in the call-stats file:¶

- contig: the contig location of this candidate
- position: the 1-based position of this candidate on the given contig
- ref\_allele: the reference allele for this candidate
- alt\_allele: the mutant (alternate) allele for this candidate
- tumor\_name: name of the tumor as given on the command line, or extracted from the BAM
- normal\_name: name of the normal as given on the command line, or extracted from the BAM
- score: for future development
- dbsnp\_site: is this a dbsnp site as defined by the dbsnp bitmask supplied to the caller
- covered: was the site powered to detect a mutation (80% power for a 0.3 allelic fraction mutation)
- power: tumor\_power \* normal\_power
- tumor\_power: given the tumor sequencing depth, what is the power to detect a mutation at 0.3 allelic fraction
- normal\_power: given the normal sequencing depth, what power did we have to detect (and reject) this as a germline variant
- total\_pairs: total tumor and normal read depth which come from paired reads
- improper\_pairs: number of reads which have abnormal pairing (orientation and distance)
- map\_Q0\_reads: total number of mapping quality zero reads in the tumor and normal at this locus
- init\_t\_lod: deprecated
- t\_lod\_fstar: CORE STATISTIC: Log of (likelihood tumor event is real / likelihood event is sequencing error )
- tumor\_f: allelic fraction of this candidated based on read counts
- contaminant\_fraction: estimate of contamination fraction used (supplied or defaulted)
- contaminant\_lod: log likelihood of ( event is contamination / event is sequencing error )
- t\_ref\_count: count of reference alleles in tumor
- t\_alt\_count: count of alternate alleles in tumor
- t\_ref\_sum: sum of quality scores of reference alleles in tumor
- t\_alt\_sum: sum of quality scores of alternate alleles in tumor
- t\_ins\_count: count of insertion events at this locus in tumor
- t\_del\_count: count of deletion events at this locus in tumor
- normal\_best\_gt: most likely genotype in the normal
- init\_n\_lod: log likelihood of ( normal being reference / normal being altered )
- n\_ref\_count: count of reference alleles in normal
- n\_alt\_count: count of alternate alleles in normal
- n\_ref\_sum: sum of quality scores of reference alleles in normal
- n\_alt\_sum: sum of quality scores of alternate alleles in normal
- judgement: final judgement of site KEEP or REJECT (not enough evidence or artifact)

---

#### Loading results for further analysis¶

In [3]:

```
samples=['S01','S02','S03','S04','S05','S06','S07',
     'S08','S09','S10','S11','S12','S13','S14','S15',
     'S16','S17','S18','S19','S20','S21','S22','S23',
     'S24','S25','S26','S27','S28','S29','S30']

for sample in samples:
    subprocess.check_output('grep -v REJECT '+ sample+'_w_pair_and_PoN.call_stats.txt > ' +
                            sample+'_w_pair_and_PoN_no_REJECT.call_stats.txt',shell=True)
```

#### Filtering out scaffolds¶

In [5]:

```
chroms=set(map(str,range(1,28)+[32]) + ['W','Z'])

mut_outputs=[]
for sample in samples:
    mut_outputs.append(pd.read_csv(+sample+'_w_pair_and_PoN_no_REJECT.call_stats.txt',sep='\t',header=1))
    filt_idx= np.array([x in chroms for x in map(str,mut_outputs[-1]['contig']) ])
    mut_outputs[-1]=mut_outputs[-1][filt_idx]
```

#### Plotting results with the default MuTect settings¶

- It can be seen that using the default settings, false positive mutations are present with very high numbers in control samples. Having one of the controls with the highest number of mutations in the dataset makes the results obtained insufficient to draw further biological conclusions.
- To improve the performance of MuTect, the LOD parameter was optimized below.

In [8]:

```
##define sample groups
control_idx=[0,4,13]+ [15,26,29]
weak_idx=[5,6,7]+[19,20,21]
strong_idx=[1,2,3,8,9,10,11,12,14]+[16,17,18,22,23,24,25,27,28]

def plot_muts(muts):
    fig,ax=plt.subplots()
    fig.set_size_inches(12,9)

    #starting clones and controls
    ax.bar(control_idx,muts[control_idx],
           facecolor='dodgerblue',edgecolor='none',label='starting clone and controls')
    #weak treatment
    ax.bar(weak_idx,muts[weak_idx],
           facecolor='salmon',edgecolor='none',label='weak mutagenic treatment')
    #strong treatment
    ax.bar(strong_idx,muts[strong_idx],
           facecolor='lightgreen',edgecolor='none',label='strong mutagenic treatment')

    #samples labels
    ax.set_xticks(0.4+np.arange(len(samples)))
    ax.set_xticklabels(samples,rotation='vertical',fontsize=14)
    #axis, and legend
    ax.set_xlabel(r'samples',fontsize=18)
    ax.set_ylabel(r'Mutations detected',fontsize=18)
    dump=ax.legend(loc='best',fancybox='true',fontsize=16)
```

In [9]:

```
muts=[]
for output in mut_outputs:
    filt_idx= np.array([x in chroms for x in map(str,output['contig']) ])
    muts.append(len(output[filt_idx]))
muts=np.array(muts)

plot_muts(muts)
```

### Plotting The LOD tuning curves¶

In [12]:

```
#set cols
cols=['lightgreen' for i in xrange(30)]
for i in control_idx:
    cols[i]='dodgerblue' 
for i in weak_idx:
    cols[i]='salmon' 

#linscale
fig,ax=plt.subplots()
fig.set_size_inches(12,9)
for output,sample,col in zip(mut_outputs,samples,cols):
    lod_vals=output.sort([u't_lod_fstar'])[u't_lod_fstar']
    ax.plot(lod_vals,len(lod_vals)-np.arange(len(lod_vals)),c=col,lw=4,label='')
ax.set_xlabel(r'LOD-value threshold',fontsize=16)
ax.set_ylabel(r'Mutations found',fontsize=16)
ax.set_xlim(5,60)
ax.set_ylim(1,5e3)
ax.grid()
#dump=ax.legend(loc='upper right',fancybox='true',fontsize=16)

#logscale
fig,ax=plt.subplots()
fig.set_size_inches(12,9)
for output,sample,col in zip(mut_outputs,samples,cols):
    lod_vals=output.sort([u't_lod_fstar'])[u't_lod_fstar']
    ax.plot(lod_vals,len(lod_vals)-np.arange(len(lod_vals)),c=col,lw=4,label='')
ax.set_xlabel(r'LOD-value threshold',fontsize=16)
ax.set_ylabel(r'Mutations found',fontsize=16)
ax.set_xlim(5,60)
ax.set_ylim(1,5e3)
ax.set_yscale('log')
ax.grid()
#dump=ax.legend(loc='upper right',fancybox='true',fontsize=16)
```

### Selecting an LOD threshold value that sufficiently decreases false positive rate, recalculating SNV counts¶

- According to the above tuning curves, an LOD threshold of around 20 would decrease false positive rates so that most control samples have fewer than 10 mutations, while treated samples have around 100-1000.
- The results filtered with this threshold can be seen below, which seem to satisfyingly separate samples based on their treatment (control / weak / strong).

In [13]:

```
muts=[]
for output in mut_outputs:
    filt_idx= np.array([x in chroms for x in map(str,output['contig']) ])
    filt_idx= filt_idx & ((output['t_lod_fstar']>20))
    muts.append(len(output[filt_idx]))
muts=np.array(muts)

plot_muts(muts)
```
