# Supplementary material for: Fast and accurate mutation detection in whole genome sequences of multiple isogenic samples with IsoMut
Source: BMC Bioinformatics. 2017 Jan 31;18:73. doi: 10.1186/s12859-017-1492-4 (PMC5282906; doi:10.1186/s12859-017-1492-4)
Supplement: Additional file 10: — Comparison of IsoMut and MuTect. Comparison of false positives rates when running IsoMut versus running MuTect with a finely tuned LOD parameter. (HTML 373 kb) [file 12859_2017_1492_MOESM10_ESM.html]

compare\_mutect\_isomut


# Additional file 10 - Comparing MuTect and IsoMut¶

### Fast and accurate mutation detection in whole genome sequences of multiple isogenic samples with IsoMut¶

##### O. Pipek, D. Ribli, J. Molnár, Á. Póti, M. Krzystanek, A. Bodor, G. E. Tusnády, Z. Szallasi, I. Csabai, and D. Szüts¶

---

## Assessing the performance of a method:¶

- An ideal method would minimize false positives (all mutations in the control samples), while still maintaining high mutation rates in treated samples (even though these are not necessarily true positives!).
- These two values can to some extent describe the performance of a unique mutation detection method and can be used to also compare these. Also, by using these two values as reference, tunable parameters of any method can be optimized.
- Quasi-ROC curves based on these two values are plotted below.

---

## Results, Conclusions:¶

#### Using MuTect with default settings:¶

- MuTect needs to be tuned heavily, as the default parameters result in overwhelming numbers of false positive mutations. In the lack of control samples, this optimizing process is not feasible, thus great care should be taken when analyzing results.

#### Maximum performance:¶

- After tuning the LOD threshold, MuTect performs sufficiently, similarly to IsoMut, resulting in even higher mutation rates in untreated samples at some false mutation rates, but a little lower at others.

#### Number of samples used:¶

- IsoMut performs better on the 30 samples analysed
- IsoMut scales better for lower sample number in case on the 15 WT samples, and it scales much better in the case of the 15 Mutant 1 samples!

#### Speed and resources:¶

- MuTect takes a rather long time to run (~ 2 days/sample), and is somewhat complicated to parallelize on our system. Per sample parallelization resulted in corrupted outputs, or java i/o errors, thus running it on multiple nodes almost always failed. Due to these reasons, the whole analysis was run on one node only, where around 7-8 instances of it could be run in parallel. For 30 samples, the whole computation time was around a week.
- On the other hand, IsoMut finishes with the same amount of data on the same single node in 2-3 hours! This translates to a performance gain of more than 50X.

#### Indels:¶

- MuTect does not detect indels, while our tools does.

---

#### Importing necessary python modules¶

In [2]:

```
import pandas as pd
import numpy as np

import matplotlib.pyplot as plt
%matplotlib inline

import seaborn as sns

import matplotlib
matplotlib.rc('font', size=18)
matplotlib.rcParams['xtick.labelsize'] = 14
matplotlib.rcParams['ytick.labelsize'] = 14
```

```
:0: FutureWarning: IPython widgets are experimental and may change in the future.
```

#### Defining plotting functions for quasi-ROC curves¶

In [5]:

```
def plot_quasy_roc(our_output,control_idx,not_control_idx,ax,label,color,linestyle,
                   xmin=-1e-3,xmax=15e-3,ymin=0,ymax=1.3):
    scan_vals=np.linspace(0,10,200)
    fp, tp = [0  for i in scan_vals ],[0  for i in scan_vals ]
    for score_lim,j in zip(scan_vals,xrange(len(scan_vals))):
        muts=[]
        for i in xrange(len(control_idx+not_control_idx)):
            try:
                filt_idx =  (our_output['#sample'] ==  i) 
            except:
                filt_idx =  (our_output['#sample_idx'] ==  i)
            filt_idx = filt_idx & ((our_output['score']>score_lim))
            muts.append(len(our_output[filt_idx]))
        muts=np.array(muts)
        fp[j] ,tp[j]=1e-3*np.mean(muts[control_idx]),1e-3*np.mean(muts[not_control_idx])
    ax.plot(fp,tp,c=color,lw=4,label=label,linestyle=linestyle)

    ax.legend(fancybox=True,loc='center left', bbox_to_anchor=(1, 0.5),fontsize=16)
    ax.set_xlim(xmin,xmax)
    ax.set_ylim(ymin,ymax)
    ax.set_xlabel('false positive mutations 1/Mbp',fontsize=18)
    dump=ax.set_ylabel('mutations detected 1/Mbp',fontsize=18)
    
    
def plot_mutect(mutect_outputs,control_idx,not_control_idx,ax,color,linestyle):
    scan_vals=range(0,200)
    fp, tp = [0  for i in scan_vals ],[0  for i in scan_vals ]
    for lod_lim,j in zip(scan_vals,xrange(len(scan_vals))):
        muts=[]
        for output in mut_outputs:
            filt_idx= np.array([x in chroms for x in map(str,output['contig']) ])
            filt_idx=filt_idx & ((output['t_lod_fstar']>lod_lim))
            muts.append(len(output[filt_idx]))
        muts=np.array(muts)
        fp[j] ,tp[j]=1e-3*np.mean(muts[control_idx]),1e-3*np.mean(muts[not_control_idx])
    ax.plot(fp,tp,c=color,lw=4,linestyle=linestyle,label='MuTect, with pair and PoN')
    
    ax.legend(fancybox=True,loc='center left', bbox_to_anchor=(1, 0.5),fontsize=16)
    ax.set_xlim(-1e-3,15e-3)
    ax.set_ylim(0,1.3)
    ax.set_xlabel('false positive mutations 1/Mbp',fontsize=18)
    dump=ax.set_ylabel('mutations detected 1/Mbp',fontsize=18)
```

## 1. Running the two methods on all 30 samples of the dataset¶

#### Defining investigated chromosomes and sample types¶

In [6]:

```
chroms=set(map(str,range(1,28)+[32]) + ['W','Z'])
cols = sns.color_palette("husl", 2)
```

In [7]:

```
samples= ['S01','S02','S03','S04','S05','S06','S07','S08','S09','S10','S11','S12','S13','S14','S15', 'S16', 'S17', 'S18', 'S19', 'S20', 'S21', 'S22', 'S23', 'S24', 'S25', 'S26', 'S27', 'S28', 'S29', 'S30']

control_idx=[0,11,14]+ [15,26,29]
not_control_idx=[1,2,3,4,5,6,7,8,9,10,12,13]+[16,17,18,19,20,21,22,23,24,25,27,28]
```

#### Loading the results of the two methods¶

- For details on usage see

  - Supplementary file 9 for MuTect
  - Supplementary file 6 for IsoMut
- (When run individually, please adjust input file names and directories.)

In [6]:

```
output_30=pd.read_csv('../post_proc_weak_sample_strong_noise/isomut/output/all_SNVs.isomut',sep='\t',header=0)

mut_outputs=[]
mut_input_dir='/nagyvinyok/adat84/sotejedlik/ribli/dt40/method/gatk/mutect_pair_pon_wt_ko/'
for sample in samples:
    mut_outputs.append(pd.read_csv(mut_input_dir+sample+'_w_pair_and_PoN_no_REJECT.call_stats.txt',sep='\t',header=1))
    filt_idx= np.array([x in chroms for x in map(str,mut_outputs[-1]['contig']) ])
    mut_outputs[-1]=mut_outputs[-1][filt_idx]
```

#### Plotting the quasi-ROC curves¶

In [8]:

```
fig,ax=plt.subplots()
fig.set_size_inches(15,9)
box=ax.get_position()
ax.set_position([box.x0, box.y0, box.width * 0.8, box.height])

plot_mutect(mut_outputs,control_idx,not_control_idx,ax,cols[0],'dashed')

plot_quasy_roc(output_30,control_idx,not_control_idx,ax,
               'IsoMut',cols[1],'solid',xmax=5e-2)
```

## 2. Running the two methods on 15 WT samples¶

#### Defining sample names and types¶

In [29]:

```
samples= ['S01','S02','S03','S04','S05','S06','S07','S08','S09','S10','S11','S12','S13','S14','S15']

control_idx=[0,11,14]
not_control_idx=[1,2,3,4,5,6,7,8,9,10,12,13]
```

#### Loading the results of the two methods¶

- (When run individually, please adjust input file names and directories.)

In [30]:

```
output_15WT=pd.read_csv('../wt_test/isomut/isomut/output/all_SNVs.isomut',sep='\t',header=0)

mut_outputs=[]
mut_input_dir='/nagyvinyok/adat84/sotejedlik/ribli/dt40/method/gatk/mutect_pair_pon_wt/'
for sample in samples:
    mut_outputs.append(pd.read_csv(mut_input_dir+sample+'_w_pair_and_PoN_no_REJECT.call_stats.txt',sep='\t',header=1))
    filt_idx= np.array([x in chroms for x in map(str,mut_outputs[-1]['contig']) ])
    mut_outputs[-1]=mut_outputs[-1][filt_idx]
```

#### Plotting the quasi-ROC curves¶

In [31]:

```
fig,ax=plt.subplots()
fig.set_size_inches(15,9)
box=ax.get_position()
ax.set_position([box.x0, box.y0, box.width * 0.8, box.height])

plot_mutect(mut_outputs,control_idx,not_control_idx,ax,cols[0],'dashed')

plot_quasy_roc(output_15WT,control_idx,not_control_idx,ax,
               'IsoMut',cols[1],'solid',xmax=5e-2,ymax=1.0)
```

## 3. Running the two methods on 15 Mutant 1 samples¶

#### Defining sample names and types¶

In [32]:

```
samples= ['S16', 'S17', 'S18', 'S19', 'S20', 'S21', 'S22', 'S23', 'S24', 'S25', 'S26', 'S27', 'S28', 'S29', 'S30']

control_idx=[0,11,14]
not_control_idx=[1,2,3,4,5,6,7,8,9,10,12,13]
```

#### Loading the results of the two methods¶

- (When run individually, please adjust input file names and directories.)

In [33]:

```
output_15KO=pd.read_csv('../ko_test/isomut/isomut/output/all_SNVs.isomut',sep='\t',header=0)

mut_outputs=[]
mut_input_dir='/nagyvinyok/adat84/sotejedlik/ribli/dt40/method/gatk/mutect_pair_pon/'
for sample in samples:
    mut_outputs.append(pd.read_csv(mut_input_dir+sample+'_w_pair_and_PoN_no_REJECT.call_stats.txt',sep='\t',header=1))
    filt_idx= np.array([x in chroms for x in map(str,mut_outputs[-1]['contig']) ])
    mut_outputs[-1]=mut_outputs[-1][filt_idx]
```

#### Plotting the quasi-ROC curves¶

In [34]:

```
fig,ax=plt.subplots()
fig.set_size_inches(15,9)
box=ax.get_position()
ax.set_position([box.x0, box.y0, box.width * 0.8, box.height])

plot_mutect(mut_outputs,control_idx,not_control_idx,ax,cols[0],'dashed')

plot_quasy_roc(output_15KO,control_idx,not_control_idx,ax,
               'IsoMut',cols[1],'solid',xmax=5e-2,ymax=2.0)
```
