# Supplementary material for: Fast and accurate mutation detection in whole genome sequences of multiple isogenic samples with IsoMut
Source: BMC Bioinformatics. 2017 Jan 31;18:73. doi: 10.1186/s12859-017-1492-4 (PMC5282906; doi:10.1186/s12859-017-1492-4)
Supplement: Additional file 11: — Runtime comparison of standard tools and IsoMut. A list of scripts and functions used to test the speed of standard mutation detection tools and IsoMut, using all resources of the available computer and using a single core only. (HTML 311 kb) [file 12859_2017_1492_MOESM11_ESM.html]

speed\_test


# Additional file 11 - Speed tests of different tools¶

### Fast and accurate mutation detection in whole genome sequences of multiple isogenic samples with IsoMut¶

#### *O. Pipek, D. Ribli, J. Molnár, Á. Póti, M. Krzystanek, A. Bodor, G. E. Tusnády, Z. Szallasi, I. Csabai, and D. Szüts*¶

---

### Technical details:¶

- The computer: 23 Gb memory, 12 cores (24 logical)
- Tests were run on chicken chromosome 28 (4.735 Mbp)
- Tested tools: IsoMut, VarScan 2, MuTect, MuTect2

---

### Using all resources of the computer:¶

With the exception of IsoMut, all other tools are limited by the finite memory of the used computer, as they need several virtual java machines to run in parallel.

- 4-5 Mutect2 processes can be run in parallel
- 6-7 Mutect1 processes can be run in parallel
- 5-6 VarScan2 processes can be run in parallel

IsoMut in only limited by the CPU in this case, thus 12 Isomut processes can be run in parallel.

**Result of using all resources:**

| tool | time | in mins | in secs | relative to IsoMut |
| --- | --- | --- | --- | --- |
| Mutect2 | 4 h | 240 | 14400 | **171x** |
| Mutect | 1h 7 min | 67 | 4020 | **48x** |
| VarScan 2 | 16 min | 16 | 960 | **11x** |
| IsoMut | 1 min 24 sec | 1.5 | 84 | **1x** |

IsoMut presents a great adventage when run on a computer with modest resources, as it is mainly I/O bound. A large computer cluster would decrease the necessary runtime of the other tools as well, making it feasible to run them on larger amounts of data. However, these high-performace computers have limited availability and in most cases mutation detection is performed on a personal computer, making it desirable to use a relatively fast tool. In these cases, IsoMut is the optimal option of the above tested tools.

---

### Using only one process:¶

Using a single core to run the above tools is not realistic, but for a more straightforward interpretation of the results, we include a test using a single core only.

- Altough some of the tools use pipe-based small parallelisation, but none of them use it heavily (more than 130%).
  - Both MuTect and MuTect2 are running on one core.
  - For VarScan2 the bottleneck is the java app, and samtools is barely running to keep it fed, thus it is basically using one core as well.
  - For IsoMut the bottleneck is samtools and IsoMut is barely running on its output, thus it is basically using one core only as well.

**Result of using one core only:**

| tool | time | in mins | in secs | relative to IsoMut |
| --- | --- | --- | --- | --- |
| Mutect2 | 21 h 6 min | 1266 | 74600 | **178x** |
| Mutect | 4 h 55 min | 295 | 17700 | **42x** |
| Varscan 2 | 1 h 20 min | 80 | 4800 | **11x** |
| IsoMut | 7 min | 7 | 420 | **1x** |

---

### Running the tests on different tools¶

#### Importing modules, defining general variables¶

In [ ]:

```
# loading modules
import os,subprocess

# going to working directory
work_dir='work_dir_path'
os.chdir(work_dir)

#inputs
input_dir='input_dir_path'
ref_genome='ref_genome_path'

#sample ids   
samples=['S01','S02','S03','S04','S12','S05','S06','S07',
         'S08','S09','S10','S11','S13','S15','S14']
samples+=['S16','S17','S18','S19','S20','S21','S22','S23',
         'S24','S25','S26','S27','S28','S29','S30']

#matched 'normal' sample ids
normal_samples = ['S02','S01','S01','S01','S15','S15','S15','S15',
                  'S15','S15','S15','S15','S15','S12','S15']
normal_samples+= ['S18','S16','S16','S16','S21','S20','S20','S20',
                  'S20','S20','S20','S30','S20','S20','S27']

#bam name conventions
bams=[sample+'_RMdup_picard_realign.bam' for sample in samples]
normal_bams=[sample+'_RMdup_picard_realign.bam' for sample in normal_samples]

#chrom
interval='28'
```

---

#### Testing with all resources¶

- **Running MuTect2**

In [ ]:

```
output_dir='mutect2'
run_mutect2_whole_pipeline(input_dir,samples,bams,normal_bams,ref_genome,output_dir,interval,
                           executor_fun_args=['--mem','4000','-C','jimgray83'])
```

- **Running MuTect**

In [ ]:

```
output_dir='mutect1'
run_mutect1_whole_pipeline(input_dir,samples,bams,normal_bams,ref_genome,output_dir,interval,
                           executor_fun_args=['--mem','4000','-C','jimgray83'])
```

- **Running VarScan 2**

In [ ]:

```
output_dir='varscan2'
run_varscan_pipeline(samples,normal_samples,bams,normal_bams,input_dir,
                     output_dir,ref_genome,interval,
                     executor_fun_args=['-c','3','--mem','2500','-C','jimgray83'])
```

- **Running IsoMut**

In [ ]:

```
%%writefile isomut/master.sh
#!/bin/bash
time python isomut_example_script.py 12
```

In [ ]:

```
%%bash
cd isomut/
sbatch -C jimgray83 -c 24 master.sh
```

---

#### Testing on one core¶

- **Running MuTect2**

In [ ]:

```
output_dir='mutect2'
run_mutect2_whole_pipeline(input_dir,samples,bams,normal_bams,ref_genome,output_dir,interval,
                           executor_fun_args=['-c','24','--mem','4000','-C','jimgray83'])
```

- **Running MuTect**

In [ ]:

```
output_dir='mutect1'
run_mutect1_whole_pipeline(input_dir,samples,bams,normal_bams,ref_genome,output_dir,interval,
                           executor_fun_args=['-c','24','--mem','4000','-C','jimgray83'])
```

- **Running VarScan 2**

In [ ]:

```
run_varscan_pipeline(samples,normal_samples,bams,normal_bams,input_dir,
                     output_dir,ref_genome,interval,
                     executor_fun_args=['-c','24','--mem','2500','-C','jimgray84'])
```

- **Running IsoMut**

In [ ]:

```
%%writefile isomut/master.sh
#!/bin/bash
time python isomut_example_script.py 1
```

In [ ]:

```
%%bash
cd isomut/
sbatch -C jimgray83 -c 24 master.sh
```

---

### Defining functions used above¶

#### Basic functions¶

In [ ]:

```
def slurm_exec_cmd(cmd,job_name,arg_list,dep_jobs=[]):
    """Execute command in slurm."""
    #write scriptfile
    with open(job_name+'.sh','w') as f:
        f.write('#!/bin/bash \n' + cmd + '\n')

    #add dependencies
    new_arg_list=arg_list+['--dependency=afterok:'+jobid for jobid in dep_jobs]
    
    #submit script to slurm
    output=subprocess.check_output(['sbatch']+new_arg_list+[job_name+'.sh'],
                                   stderr=subprocess.STDOUT)
    #return the job id
    job_id = output.split()[-1]
    return job_id
```

#### MuTect2 functions¶

Command descriptions can be found here:

- https://software.broadinstitute.org/gatk/gatkdocs/org\_broadinstitute\_gatk\_tools\_walkers\_cancer\_m2\_MuTect2.php

In [ ]:

```
def run_mutect2_whole_pipeline(input_dir,samples,bams,normal_bams,
                               ref_genome,output_dir,interval,
                               executor_fun=slurm_exec_cmd,
                               executor_fun_args=['--mem','4000','-C','jimgray83']):
    """Run the whole mutect2 pipeline for unique mutation detection."""
    #artifact detection mode
    art_jobs=[]
    for sample,bam in zip(samples,bams):
        art_jobs.append(run_mutect2_artif_detect(sample,bam,input_dir,output_dir,ref_genome,interval,
                                                 executor_fun,executor_fun_args))
    
    #combining arts into pons
    combine_jobs=[]
    for sample in samples:
        combine_jobs.append(run_mutect2_cobine_into_pon(sample,samples,output_dir,ref_genome,
                                                        executor_fun,executor_fun_args,art_jobs))
    
    
    #running mutect2 with pair and pon
    for sample,bam,normal_bam in zip(samples,bams,normal_bams):
        run_mutect2_w_pair_and_pon(sample,bam,normal_bam,
                                   input_dir,output_dir,ref_genome,interval,
                                   executor_fun,executor_fun_args,combine_jobs)
    
    return

def run_mutect2_artif_detect(sample,bam,input_dir,output_dir,ref_genome,interval,
                             executor_fun,executor_fun_args):
    """Run mutect2 in artifact detection mode."""
    #output,log files
    output=output_dir+'/'+sample + '_art.vcf'
    log=output_dir+'/'+sample+'_art.log '

    #gatk command
    cmd='time $JAVA_HOME/bin/java -Xmx4g -jar $GATK_3_6_PATH/GenomeAnalysisTK.jar '
    cmd+=' -T MuTect2 ' 
    cmd+=' -R '+ ref_genome
    cmd+=' -I:tumor '+ input_dir+'/'+bam
    cmd+=' --artifact_detection_mode '
    cmd+=' -L ' + interval
    cmd+=' -o ' + output
    cmd+=' 2> '+ log
    
    #send to executor function
    job_name='art2_'+sample
    return executor_fun(cmd,job_name,executor_fun_args)


def run_mutect2_cobine_into_pon(sample,samples,output_dir,ref_genome,
                                executor_fun,executor_fun_args,dep_jobs):
    """
    Combine mutect2 artifact detection vcf-s into a panel of normals.
    
    Create 1 panel of normal for every sample, including all the other samples.
    """
    #output,log
    output=output_dir+'/'+sample+'_pon.vcf'
    log=output_dir+"/"+sample+'_poncombine.log '

    #gatk command
    cmd='time $JAVA_HOME/bin/java -Xmx4g -jar $GATK_3_6_PATH/GenomeAnalysisTK.jar '
    cmd+=' -T CombineVariants ' 
    cmd+=' -R '+ ref_genome
    #include all the other samples
    for other_sample in samples:
        if (other_sample != sample):
            cmd+=' -V '+ output_dir+'/'+other_sample+'_art.vcf'
    cmd+=' -minN 2 '
    cmd+=' --setKey "null" '
    cmd+=' --filteredAreUncalled '
    cmd+=' --filteredrecordsmergetype KEEP_IF_ANY_UNFILTERED '
    cmd+=' -o ' + output
    cmd+=' 2> '+ log
    
    #send to executor function
    job_name='pon2_'+sample
    return executor_fun(cmd,job_name,executor_fun_args,dep_jobs)


def run_mutect2_w_pair_and_pon(sample,bam,normal_bam,
                               input_dir,output_dir,ref_genome,interval,
                               executor_fun,executor_fun_args,dep_jobs):
    """Run mutect2 with a normal pair and a panel of normals."""
    #output,log
    output = output_dir+"/"+sample+'_w_pair_and_pon.vcf'
    log = output_dir+"/"+sample+'_w_pair_and_pon.log'

    #gatk command
    cmd='time $JAVA_HOME/bin/java -Xmx4g -jar $GATK_3_6_PATH/GenomeAnalysisTK.jar  '
    cmd+=' -T MuTect2 ' 
    cmd+=' -R '+ ref_genome
    cmd+=' -I:normal '+ input_dir+'/'+normal_bam
    cmd+=' -I:tumor '+ input_dir+'/'+bam
    cmd+=' --normal_panel '+ output_dir+"/"+sample+'_pon.vcf'
    cmd+=' -L ' + interval
    cmd+=' -o ' + output
    cmd+=' 2> '+ log

    #send to executor function
    job_name='mut2_'+sample
    return executor_fun(cmd,job_name,executor_fun_args,dep_jobs)
```

#### MuTect functions¶

In [ ]:

```
def run_mutect1_whole_pipeline(input_dir,samples,bams,normal_samples,ref_genome,output_dir,interval,
                               executor_fun=slurm_exec_cmd,
                               executor_fun_args=['--mem','4000','-C','jimgray83']):
    """Run the whole mutect2 pipeline for unique mutation detection."""
    #artifact detection mode
    art_jobs=[]
    for sample,bam in zip(samples,bams):
        art_jobs.append(run_mutect1_artif_detect(sample,bam,input_dir,output_dir,ref_genome,interval,
                                                 executor_fun,executor_fun_args))
    
    #combining arts into pons
    combine_jobs=[]
    for sample in samples:
        combine_jobs.append(run_mutect1_cobine_into_pon(sample,samples,output_dir,ref_genome,
                                                        executor_fun,executor_fun_args,art_jobs))
    
    #running mutect2 with pair and pon
    for sample,bam,normal_bam in zip(samples,bams,normal_bams):
        run_mutect1_w_pair_and_pon(sample,bam,normal_bam,
                                   input_dir,output_dir,ref_genome,interval,
                                   executor_fun,executor_fun_args,combine_jobs)
    
    return

def run_mutect1_artif_detect(sample,bam,input_dir,output_dir,ref_genome,interval,
                             executor_fun,executor_fun_args):
    """Run mutect2 in artifact detection mode."""
    #log,outputs
    log=output_dir+'/'+sample+'_art.log '
    call_stats=output_dir+"/" + sample + '_art.vcf'
    coverage_file=output_dir+"/" + sample+'_coverage_wig.txt'

    #gatk command
    cmd='time java -Xmx2g -jar $GATK_PATH/mutect-1.1.7.jar '
    cmd+=' -T MuTect ' 
    cmd+=' -R '+ ref_genome
    cmd+=' -I:tumor '+ input_dir+'/'+ bam
    cmd+=' --artifact_detection_mode '
    cmd+=' -L ' + interval
    cmd+=' -vcf ' + call_stats
    cmd+=' --coverage_file '+ coverage_file
    cmd+=' 2> '+ log
    
    #send to executor function
    job_name='art1_'+sample
    return executor_fun(cmd,job_name,executor_fun_args)


def run_mutect1_cobine_into_pon(sample,samples,output_dir,ref_genome,
                                executor_fun,executor_fun_args,dep_jobs):
    """
    Combine mutect2 artifact detection vcf-s into a panel of normals.
    
    Create 1 panel of normal for every sample, including all the other samples.
    """
    #input,output,log,scipt_file
    output=output_dir+'/'+sample+'_pon.vcf'
    log=output_dir+"/"+sample+'_poncombine.log '

    #gatk command
    cmd='time java -Xmx4g -jar $GATK_PATH/GenomeAnalysisTK.jar '
    cmd+=' -T CombineVariants ' 
    cmd+=' -R '+ ref_genome
    #include all the other samples
    for other_sample in samples:
        if (other_sample != sample):
            cmd+=' -V '+ output_dir+'/'+other_sample+'_art.vcf'
    cmd+=' -minN 2 '
    cmd+=' --filteredAreUncalled '
    cmd+=' --filteredrecordsmergetype KEEP_IF_ANY_UNFILTERED '
    cmd+=' --genotypemergeoption UNIQUIFY '
    
    cmd+=' -o ' + output
    cmd+=' 2> '+ log
    
    #send to executor function
    job_name='pon1_'+sample
    return executor_fun(cmd,job_name,executor_fun_args,dep_jobs)


def run_mutect1_w_pair_and_pon(sample,bam,normal_bam,input_dir,output_dir,ref_genome,interval,
                               executor_fun,executor_fun_args,dep_jobs):
    """Run mutect2 with a normal pair and a panel of normals."""
    #inputs,output,pon,log,script_file
    output = output_dir+"/"+sample+'_w_pair_and_pon.vcf'
    log = output_dir+"/"+sample+'_w_pair_and_pon.log'

    #gatk command
    cmd='time java -Xmx2g -jar $GATK_PATH//mutect-1.1.7.jar  '
    cmd+=' -T MuTect ' 
    cmd+=' -R '+ ref_genome
    cmd+=' -I:normal '+ input_dir+'/'+normal_bam
    cmd+=' -I:tumor '+ input_dir+'/'+bam
    cmd+=' --normal_panel '+ output_dir+"/"+sample+'_pon.vcf'
    cmd+=' -L ' + interval
    cmd+=' -o ' + output
    cmd+=' 2> '+ log

    #send to executor function
    job_name='mut1_'+sample
    return executor_fun(cmd,job_name,executor_fun_args,dep_jobs)
```

#### VarScan 2 functions¶

In [ ]:

```
def run_varscan_pipeline(tum_samples,norm_samples,tum_bams,norm_bams,
                         input_dir,output_dir,ref_genome,region,
                         executor_fun=slurm_exec_cmd,
                         executor_fun_args=['-c','3','--mem','2500','-C','jimgray83'],
                         **kwargs):
    """Run varscan on all the files."""
    for sample,normal_sample,bam,normal_bam in zip(tum_samples,norm_samples,tum_bams,norm_bams):
        run_varscan_som(sample,normal_sample,bam,normal_bam,input_dir,output_dir,ref_genome,region,
                        executor_fun,executor_fun_args,**kwargs)
    return
    
def run_varscan_som(tum_sample,norm_sample,tum_bam,norm_bam,
                    input_dir,output_dir,ref_genome,region,
                    executor_fun,executor_fun_args,
                    min_cov=10,min_var_freq=0.08,somatic_p_value=0.05):
    """Run VarScan somatic on a tumor normal pair."""
    #input files with full path
    full_norm_bam=input_dir+norm_bam
    full_tum_bam=input_dir+tum_bam
    
    #output file
    output=output_dir+'/'+tum_sample+'_'+norm_sample+'.vsc'

    #create pileup commands
    cmd_mpileup =' <(samtools mpileup -B -q 1 -r '+region+' -f '+ ref_genome +  ' ' + full_norm_bam+')'
    cmd_mpileup+=' <(samtools mpileup -B -q 1 -r '+region+' -f '+ ref_genome +  ' ' + full_tum_bam +')'

    #varscan command
    cmd='time java -Xmx2g -jar $VARSCAN_PATH/VarScan.v2.3.7.jar somatic '+ cmd_mpileup + ' '+ output
    cmd+=' --min-coverage ' +str(min_cov)
    cmd+=' --min-var-freq ' + str(min_var_freq)
    cmd+=' --somatic-p-value '+str(somatic_p_value)
    
    #send to executor function
    job_name='vs'+tum_sample
    return executor_fun(cmd,job_name,executor_fun_args)
```

#### IsoMut script¶

In [ ]:

```
%%writefile isomut/isomut_example_script.py
#!/usr/bin/env python

#################################################
# importing the wrapper
#################################################
import sys,os
#load the parallel wrapper function
from isomut.isomut_wrappers import run_isomut

#set the number iof concurrent processes to run
n_p=int(sys.argv[1])


#################################################
# defining administrative parameters
#################################################
#using parameter dictionary, beacause there are awful lot of parameters
params=dict()
#minimum number of blocks to run
# usually there will be 10-20 more blocks
params['n_min_block']=200
#number of concurrent processes to run
params['n_conc_blocks']=n_p
#genome
params['ref_fasta']='ref_genome_path'
#input dir output dir
params['input_dir']='input_dir_path'
params['output_dir']='isomut_test_output/'
#the bam files used
samples = ['S01','S02','S03','S04','S05','S06','S07','S08',
                         'S09','S10','S11','S12','S13','S14','S15',
                         'S16','S17','S18','S19','S20','S21','S22','S23',
                         'S24','S25','S26','S27','S28','S29','S30']
params['bam_filenames']=[sample + '_RMdup_picard_realign.bam' for sample in samples]

#limit chromosomes
params['chromosomes']=['28']

#################################################
# defining mutation calling parameters
#    default values here ...
#################################################
params['min_sample_freq']=0.21
params['min_other_ref_freq']=0.93
params['cov_limit']=5
params['base_quality_limit']=30
params['min_gap_dist_snv']=0
params['min_gap_dist_indel']=20

#################################################
# and finally run it
#################################################
run_isomut(params)
```
